# Generation and characterization of stable pig pregastrulation epiblast stem cell lines

**DOI:** 10.1038/s41422-021-00592-9

**Published:** 2021-11-30

**Authors:** Minglei Zhi, Jinying Zhang, Qianzi Tang, Dawei Yu, Shuai Gao, Dengfeng Gao, Pengliang Liu, Jianxiong Guo, Tang Hai, Jie Gao, Suying Cao, Zimo Zhao, Chongyang Li, Xiaogang Weng, Mengnan He, Tianzhi Chen, Yingjie Wang, Keren Long, Deling Jiao, Guanglei Li, Jiaman Zhang, Yan Liu, Yu Lin, Daxin Pang, Qianqian Zhu, Naixin Chen, Jingjing Huang, Xinze Chen, Yixuan Yao, Jingcang Yang, Zicong Xie, Xianya Huang, Mengxin Liu, Ran Zhang, Qiuyan Li, Yiliang Miao, Jianhui Tian, Xingxu Huang, Hongsheng Ouyang, Bofeng Liu, Wei Xie, Qi Zhou, Hongjiang Wei, Zhonghua Liu, Caihong Zheng, Mingzhou Li, Jianyong Han

**Affiliations:** 1 State Key Laboratory of Agrobiotechnology, College of Biological Sciences, China Agricultural University, Beijing, China; 2 Institute of Animal Genetics and Breeding, College of Animal Science and Technology, Sichuan Agricultural University, Chengdu, Sichuan China; 3 State Key Laboratory of Stem Cell and Reproductive Biology, Institute of Zoology, Chinese Academy of Sciences, Beijing, China; 4 Institute for Stem Cell and Regenerative Medicine, Chinese Academy of Sciences, Beijing, China; 5 Beijing Institute for Stem Cell and Regenerative Medicine, Beijing, China; 6 Key Laboratory of Animal Genetics, College of Animal Science and Technology, China Agricultural University, Beijing, China; 7 State Key Laboratory for Conservation and Utilization of Bio-Resources in Yunnan, Yunnan Agricultural University, Kunming, Yunnan China; 8 Animal Science and Technology College, Beijing University of Agriculture, Beijing, China; 9 Key Laboratory of Animal Cellular and Genetics Engineering of Heilongjiang Province, College of Life Science, Northeast Agricultural University, Harbin, Heilongjiang China; 10 School of Life Science and Technology, ShanghaiTech University, Shanghai, China; 11 Jilin Provincial Key Laboratory of Animal Embryo Engineering, College of Animal Sciences, Jilin University, Changchun, Jilin China; 12 Institute of Stem Cell and Regenerative Biology, College of Animal Science and Veterinary Medicine, Huazhong Agricultural University, Wuhan, Hubei China; 13 Center for Stem Cell Biology and Regenerative Medicine, MOE Key Laboratory of Bioinformatics, THU-PKU Center for Life Sciences, School of Life Sciences, Tsinghua University, Beijing, China; 14 Key Laboratory of Genomic and Precision Medicine, Beijing Institute of Genomics, Chinese Academy of Sciences, and China National Center for Bioinformation, Beijing, China

**Keywords:** Pluripotent stem cells, Developmental biology

## Abstract

Pig epiblast-derived pluripotent stem cells are considered to have great potential and broad prospects for human therapeutic model development and livestock breeding. Despite ongoing attempts since the 1990s, no stably defined pig epiblast-derived stem cell line has been established. Here, guided by insights from a large-scale single-cell transcriptome analysis of pig embryos from embryonic day (E) 0 to E14, specifically, the tracing of pluripotency changes during epiblast development, we developed an in vitro culture medium for establishing and maintaining stable pluripotent stem cell lines from pig E10 pregastrulation epiblasts (pgEpiSCs). Enabled by chemical inhibition of WNT-related signaling in combination with growth factors in the FGF/ERK, JAK/STAT3, and Activin/Nodal pathways, pgEpiSCs maintain their pluripotency transcriptome features, similar to those of E10 epiblast cells, and normal karyotypes after more than 240 passages and have the potential to differentiate into three germ layers. Strikingly, ultradeep in situ Hi-C analysis revealed functional impacts of chromatin 3D-spatial associations on the transcriptional regulation of pluripotency marker genes in pgEpiSCs. In practice, we confirmed that pgEpiSCs readily tolerate at least three rounds of successive gene editing and generated cloned gene-edited live piglets. Our findings deliver on the long-anticipated promise of pig pluripotent stem cells and open new avenues for biological research, animal husbandry, and regenerative biomedicine.

## Introduction

During development, the epiblast is specified from the inner cell mass (ICM) of the blastocyst and generates all of the somatic and germ cell lineages, giving rise to the embryo proper.^1,2^ In pluripotency, epiblast cells are an important source of pluripotent stem cells (PSCs), including mouse embryonic stem cells (ESCs), which are derived from naïve epiblasts of blastocysts,^3–6^ and epiblast stem cells (EpiSCs), which are derived from epiblasts at later developmental stages.^7–9^ Recently, formative (or “intermediate”) pluripotent stem cells were successfully derived from mouse pregastrulation epiblasts.^10–12^ Human conventional ESCs are derived from the ICM of the blastocyst and show primed pluripotency features similar to those of mouse EpiSCs,^8,13^ and human PSCs with naïve or formative pluripotency states can also be obtained.^10,14,15^

Compared to many other animal models, pigs are more similar to humans in terms of embryo development,^16^ anatomy,^17,18^ and physiology,^19^ so it follows that stable pig PSCs derived from epiblasts should be excellent models for gaining insights into the properties of human PSCs, potentially enabling informative applications for human developmental modeling.^19^ It has been anticipated that the combination of stable pig PSCs and accurate multiple gene-editing technology will have large impacts on both biomedical research and animal breeding for agriculture.^20,21^

Surprisingly, despite extensive and ongoing attempts since the 1990s, no long-term passaged stable pig PSC lines have yet been derived from ICMs or epiblasts at different stages.^22–28^ It has been proposed that interspecies differences during embryogenesis and heterogeneity in the signaling pathways that regulate pluripotency during early embryonic development likely explain the decades-long failure to adapt cell culture techniques from humans and mice for use in pigs.^29,30^

Regarding the molecular basis of embryogenesis and ESC establishment, studies in pigs lag considerably behind the now very-high-resolution profiling atlases available for mice, humans, and nonhuman primates. Whereas there have been replicated large-scale studies based on single-cell RNA sequencing (scRNA-seq) technologies to profile early embryos and to trace the lineage trajectories and embryo-to-stem-cell transition in mice, humans, and monkeys,^3,31–36^ the existing single-cell transcriptome studies on pig embryos do not provide an accurate and high-resolution transcriptome map for preimplantation embryos, likely owing to the difficulty of obtaining sufficient numbers of embryonic cells at different development stages.^29,30,37,38^

Here, we collected preimplantation pig embryos and conducted scRNA-seq of all stages (each day during E0–E14) to comprehensively profile the molecular basis of early pig embryonic development and pluripotency changes. Subsequently, building on mechanistic insights into WNT signaling from our profiling work, we developed a culture medium (termed “3i/LAF”) that readily supports the establishment of stable pig epiblast stem cell lines from E8–E10 pregastrulation epiblasts (designated pgEpiSCs). Extensive characterization revealed that these pgEpiSCs display the pluripotency and the molecular properties of pregastrulation epiblasts, show domed morphology, express pluripotency markers, maintain stability over 240 passages, and have capacities for both high-efficiency teratoma formation and differentiation into diverse cell types. We achieved multiple successive rounds of gene editing with pgEpiSCs and then used gene-edited donor cells for cell nuclear transfer to successfully generate homozygous edited piglets.

## Results

### scRNA-seq reveals lineage segregation during pig embryonic development

**Fig. 1:**
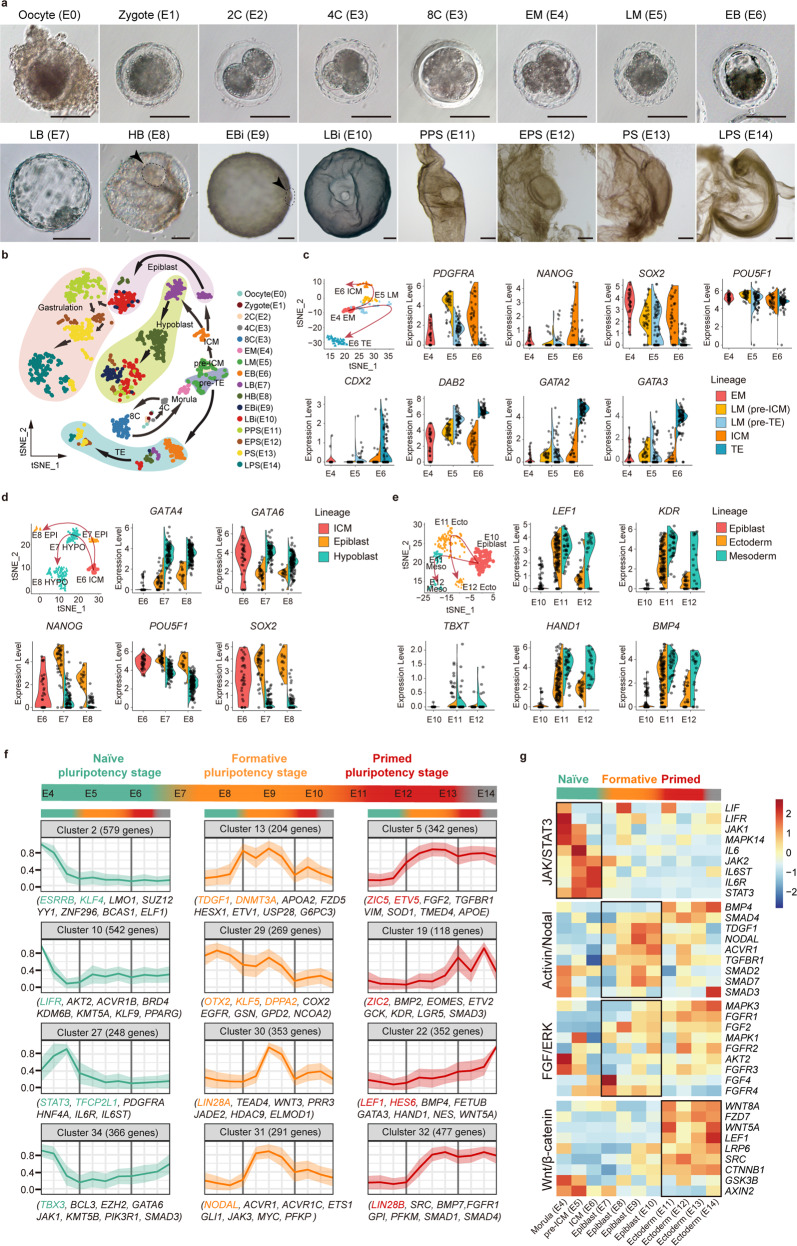
Lineage segregation identification and tracing of pluripotency changes during embryonic development. **a** Morphology of pig embryos collected from embryonic day (E) 0 to E14 for scRNA-seq analysis. A total of 16 developmental stages were included, i.e., oocyte (E0), zygote (E1), 2C (2-cell stage embryo, E2), 4C (4-cell stage embryo, E3), 8C (8-cell stage embryo, E3), EM (early morula, E4), LM (late morula, E5), EB (early blastula, E6), LB (late blastula, E7), HB (hatched blastula, E8), EBi (early bilaminar embryo, E9), LBi (late bilaminar embryo, E10), PPS (pre-primitive streak embryo, E11), EPS (early primitive streak embryo, E12), PS (primitive streak embryo, E13), and LPS (late primitive streak embryo, E14). Scale bar for E0–E8, 100 μm; scale bar for E9–E14, 500 μm. Arrows indicate the position of E8 and E9 epiblasts. **b** t-SNE plot showing the transcriptional similarity of all pig embryonic cells; different colored dots represent the indicated embryonic days and developmental stages; background colors represent the indicated lineages; arrows represent known developmental trajectories. **c** t-SNE plot of E4 EM, E5 LM, and E6 EB stage embryonic cells; arrow tracks indicate inner cell mass (ICM)/trophectoderm (TE) lineage separation; main lineage separation marker genes are shown in violin plots. **d** as in **c**, but for E6 EB, E7 LB, and E8 HB stage embryonic cells; arrow tracks indicate epiblast (EPI)/hypoblast (HYPO) lineage separation. **e** as in **c**, but for E10 LBi, E11 PPS, and E12 EPS stage embryonic cells; arrow tracks indicate ectoderm (Ecto)/mesoderm (Meso) lineage separation. **f** Representative clusters of genes with similar expression trends showing the changes in naïve, formative, and primed pluripotency genes in morula (EM and pre-ICM), ICM, epiblast, and ectoderm cells during E4–E14. Genes shown in green, yellow, and red represent possible naïve, formative, and primed pluripotency genes, respectively;^10,11^ the remaining genes (in black) were predicted from the clusters. **g** Expression changes in JAK/STAT3, Activin/Nodal, FGF/ERK, and Wnt/β-catenin signaling pathway-associated genes in morula (EM and pre-ICM), ICM, epiblast, and ectoderm cells from E4 to E14. The gradient from blue to red on the right side of the heatmap indicates low to high expression of genes. The gradient from green to red at the top of the heatmap indicates naïve, formative, and primed pluripotency state changes. See also Supplementary information, Figs. S1 and S2.

To profile the molecular basis of lineage segregation and pluripotency changes during pig embryonic development, we performed scRNA-seq using a modified single-cell tagged reverse transcription sequencing (STRT-seq) protocol (see Materials and methods)^39,40^ and, after stringent filtration, ultimately obtained data for 1458 retained single cells from pig oocytes and embryos sampled from E1 to E14 (Fig. 1a and Supplementary information, Table S1). We identified the cell types at different stages and characterized distinct lineage differentiation processes by a shared nearest neighbor (SNN) algorithm and t-distributed stochastic neighbor embedding (t-SNE).^41^ The cells were categorized into particular embryonic lineages at distinct times based on known marker genes of differentiation and pluripotency (Fig. 1b and Supplementary information, Data S3).^29,33,42^ The cells were also classified into clusters exhibiting different gene expression characteristics informed by functional enrichment of specifically expressed genes and a coexpressed gene network (Supplementary information, Fig. S1d and Table S2). The cell assignment was further supported by pseudotime and velocity analysis (Supplementary information, Fig. S1a–c).

The first lineage segregation that we detected in pig embryos was initiated at the E5 late morula stage (Fig. 1c). Two subsets of late morula stage cells, termed pre-ICM and pre-trophectoderm (pre-TE) cells, displayed differential expression of classic precursor marker genes for ICMs (e.g., *PDGFRA*) or TEs (e.g., *DAB2*)^34,38,43^ (Fig. 1c). At the E6 early blastula stage, the ICM and TE cells represented two populations, with upregulation of *PDGFRA, NANOG*, and *SOX2* in ICMs and *CDX2*, *DAB2, GATA2*, and *GATA3* in TEs (Fig. 1c). The heterogeneous expression of *GATA6* (a hypoblast marker) and *NANOG* (an epiblast marker) was detected in the ICMs on E6 (Fig. 1d), indicating the beginning of the segregation of the second lineage.^44,45^

At the E7 late blastula stage, *GATA6-* and *NANOG*-positive cells were divided into two populations (Fig. 1d), indicating the end of the second segregation, which established the hypoblast (*GATA4*^+^ and *GATA6*^+^) and epiblast (*NANOG*^+^ and *SOX2*^*+*^) lineages in the embryos. From E7 to E10, the number of epiblast and hypoblast cells increases rapidly, and hypoblasts spread inside the TE to form a complete cavity, called the gastrocoele;^46^ epiblasts expressed high levels of pluripotency marker genes including *NANOG*, *POU5F1*, and *SOX*2 (Fig. 1d) until the mesoderm began to form at E11 (marked by the upregulation of gastrulation marker genes such as *LEF1, KDR, TBXT, HAND1*, and *BMP4*) (Fig. 1e). The molecular genetic regulation trends we observed throughout development from E0–E14 are fully consistent with the well-characterized patterns described in previously reported histological and morphological evidence.^16,46^ In addition, based on the cell assignment, we defined the top specifically expressed genes for each embryonic lineage; these genes may serve as potential markers for lineage identification during pig embryo development (Supplementary information, Fig. S1e and Table S2). This congruence emphasizes both the representativeness and practical utility of this rich, single-cell-resolution transcriptomic resource that will support both applied biotechnological efforts and basic studies of embryonic regulation.

### Tracing pluripotency changes during pig epiblast development

To trace the pluripotency changes during pig epiblast development and to identify impacts on signaling pathways, we classified 11,113 differentially expressed genes (DEGs) by pairwise comparison in the cells from E4 early morulae, E5 pre-ICMs, E6 ICMs, E7–E10 epiblasts, and E11–E14 ectoderms into 36 clusters based on the expression trends across embryonic stages (Supplementary information, Table S3 and Data S3). Interestingly, the expression of representative naïve pluripotency genes (e.g., *ESRRB, KLF4, LIFR, STAT3, TFCP2L1*, and *TBX3*) was sharply reduced in cells from E4 early morulae to E7 epiblasts. In contrast, the expression of classical primed pluripotency genes (e.g., *ZIC5, ETV5, ZIC2, LEF1, BMP4*, and *LIN28B*) was elevated after E7 (Fig. 1f). Formative state marker genes (e.g., *TDGF1*, *DNMT3A*, *OTX2*, *KLF5*, *LIN28A*, and *NODAL*) exhibited relatively high expression in E7–E10 epiblasts but were subsequently downregulated in E11–E13 ectoderms (Fig. 1f). In addition, the annotation of the expression tendencies for the three pluripotency states was further supported by pluripotency regulatory network construction and functional term enrichment analyses^47^ (Supplementary information, Fig. S2a, b and Table S3). The rapid loss of naïve pluripotency that we observed before E7 epiblast formation — consistent with a previous report^29^ — may help explain the previous failures to derive naïve ES cells from the epiblasts of E7 late blastocysts in pigs. Furthermore, these data suggest that epiblasts maintain a steady formative state from E7 to E10 and may support the establishment of stable pluripotent stem cell lines.

We hypothesized that the generation of epiblast-derived stem cells requires stabilization of pluripotency-related signaling and inhibition of differentiation. Ultimately, we focused on four signaling pathways: JAK/STAT3, Activin/Nodal, FGF/ERK, and Wnt/β-catenin. We found that hub genes in the JAK/STAT3 signaling pathway were highly expressed in cells from E4 early morulae to E6 ICMs but declined sharply after E7 epiblast formation (Fig. 1g), consistent with the expression patterns of naïve pluripotency marker genes during pig epiblast development (Fig. 1f). The receptors of Activin A and FGF2 are highly expressed in epiblasts from E7 to E10 (Fig. 1g), suggesting that the proliferation and maintenance of pig epiblasts require the presence of Activin A and FGF2, which are also known to be required for self-renewal of mouse EpiSCs and human conventional ESCs.^48^ Interestingly, we noted significantly increased Wnt/β-catenin signaling activity during the epiblast-to-ectoderm developmental transition from E10 to E11 (Fig. 1g), suggesting that inhibition of Wnt/β-catenin signaling may be required for the derivation and maintenance of stable pig epiblast stem cells.

### Generation of stable pig pgEpiSC lines from E10 epiblasts

Our insights from scRNA-seq profiling indicate that pig embryo naïve pluripotency markers decline sharply during the first and second lineage differentiation, and that the formative pluripotent state can maintain a relatively stable stage in pregastrulation epiblasts. In addition, the establishment of pgEpiSCs should be facilitated by the use of small-molecule inhibitors related to WNT signaling to prevent gastrulation, as well as TGF-β superfamily and FGF family cytokines to promote epiblast self-renewal. Pursuing this with E10 epiblasts, we designed CI/LAF culture conditions (Supplementary information, Fig. S3a), which included the WNT inhibitor IWR-1-endo (I) to block canonical WNT signaling and the GSK3β inhibitor CHIR99021 (C), which has been reported to balance IWR-1-endo function and coordinate maintenance of the long-term self-renewal of mEpiSCs and conventional hESCs.^49^ We also used the cytokines Activin A (A) and FGF2 (F), which are essential for the maintenance of pEpiSC pluripotency and self-renewal,^28^ as well as LIF (L), which was reported to enforce the pluripotency of mEpiSCs^50^. Simultaneously, we designed the conditions lacking the cytokines and/or IWR-1-endo and tested the reported culture media for naïve hESCs^15,51^ and extended pluripotent stem cells (EPSCs)^52,53^ (Supplementary information, Fig. S3a). The results indicated that the cells in the CI/LAF culture condition showed dome-like colony morphology and more stable passage ability compared with cells grown under the other conditions (Supplementary information, Fig. S3a–c). Unexpectedly, immunostaining indicated that POU5F1 and GATA6 were heterogeneously expressed in the cell colonies (Supplementary information, Fig. S3d). This may be due to the concentration of CHIR99021 used, as this factor has a dual role in pluripotency maintenance in mouse and human PSCs, with low concentrations promoting self-renewal and high concentrations promoting differentiation.^49^ Therefore, we titrated the concentration of CHIR99021 and found that 0.5–1 μM CHIR99021 promoted the expression of *POU5F1* and *ESRRB* and did not cause an increase in *GATA6* expression (Supplementary information, Fig. S3e, f).

**Fig. 2:**
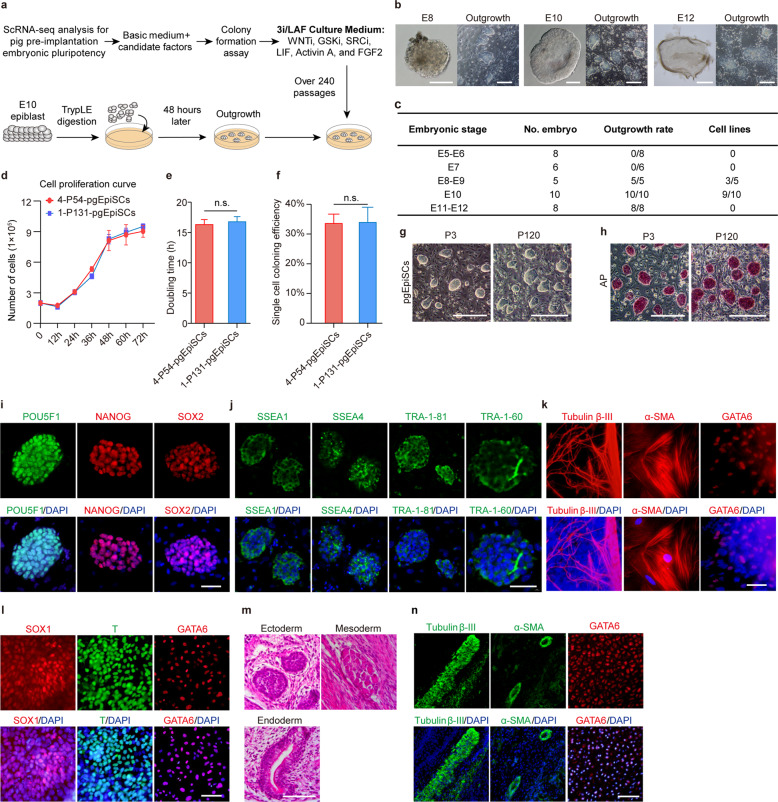
Generation and characteristics of pgEpiSCs. **a** Strategies for the establishment of stable pgEpiSCs. **b** Morphological comparison of embryonic epiblasts at different embryonic stages and primary outgrowths derived from embryonic epiblast cells. From left to right, E8, E10, and E12 embryonic discs (left) and outgrowth (right). The outgrowths of E8 and E10 epiblasts were domed, while the outgrowths of E12 epiblasts were flat and irregular. Scale bars for E8 and E10 embryonic discs, 100 μm; scale bars for the E12 embryonic disc, 400 μm; scale bars for all outgrowths, 200 μm. **c** Efficiency of outgrowths derived from epiblasts at different embryonic stages and cell lines established in 3i/LAF culture medium. **d** Cell proliferation curve of pgEpiSCs. The initial cell count was 2 × 10^5^. **e** Population doubling time of pgEpiSCs. **f** Single-cell cloning efficiency of pgEpiSCs. **g** Morphology of low- and high-passage pgEpiSC colonies. Scale bars, 200 μm. **h** Alkaline phosphatase (AP) staining assay for low and high passage numbers of pgEpiSC colonies. Scale bars, 200 μm. **i** Immunostaining of the pluripotency markers POU5F1, NANOG, and SOX2 in pgEpiSCs. DAPI was used to stain nuclei. Scale bar, 50 μm. **j** Immunostaining of pluripotency surface markers SSEA1, SSEA4, TRA-1-81, and TRA-1-60 in the pgEpiSCs. DAPI for staining of nuclei. Scale bar, 50 μm. **k** In vitro EB differentiation assay. Immunostaining for the ectodermal neuro-specific marker protein Tubulin β-III, mesodermal muscle-specific marker protein α-SMA, and endoderm-specific marker protein GATA6. DAPI was used for nucleus staining. Scale bar, 50 μm. **l** Immunostaining of pgEpiSCs after directional induced differentiation. SOX1 is a neural ectoderm marker, T is a mesoderm marker, GATA6 is an endoderm marker, and the nuclei are indicated by DAPI. Scale bar, 200 μm. **m** In vivo teratoma formation assay. Haematoxylin and eosin (H&E) staining of teratomas derived from pgEpiSCs. Scale bar, 100 μm. **n** Representative immunofluorescence images showing that the teratoma contains ecto/meso/endoderm. Scale bar, 100 μm. For **d**–**f**, the error bar indicates ± SD (*n* = 3, independent experiments); cell line 4 at passage 54 (4-P54-pgEpiSCs) and cell line 1 at passage 131 (1-P131-pgEpiSCs) were used. n.s., *P* ≥ 0.05. For **g**, **h**, P3 and P120 represent the pgEpiSCs at passage 3 and 120, respectively. For **g**–**n**, similar results were obtained in three independent experiments. See also Supplementary information, Figs. S3 and S4.

In view of the fact that the E10 epiblasts exist at the critical point during embryonic development just before gastrulation, these cells tend to undergo spontaneous epithelial–mesenchymal transition (EMT) unless acted upon by external factors (Fig. 1e). We detected the expression of EMT-related genes in cells derived from different conditions. As expected, all the cells cultured in the tested conditions expressed high levels of *SRC* and *WNT5A* (Supplementary information, Fig. S3g), indicating the occurrence of EMT.^54^ We therefore explored the use of the SRC inhibitor WH-4-023 in our culture medium to block the EMT process and ultimately developed a medium containing three inhibitors (CHIR99021, IWR-1-endo, and WH-4-023) and three cytokines (LIF, Activin A, and FGF2), termed “3i/LAF” culture medium (Fig. 2a and Supplementary information, Fig. S3). Under culture in 3i/LAF, we found that E10 epiblasts yielded more efficient establishment rates than epiblasts of other stages (Fig. 2b, c). The pgEpiSCs derived from E10 epiblasts could be passaged at the single-cell level through enzymatic dissociation every 2–3 days at passage ratios between 1:3 and 1:5. The doubling time for pgEpiSC proliferation was approximately 16 h (Fig. 2d, e; see Materials and methods), and the efficiency of single-cell colony formation was approximately 33.83% (Fig. 2f).

We found that the pgEpiSCs retained their dome-shaped colony morphology (Fig. 2g), positive AP staining (Fig. 2h), and normal karyotypes (Supplementary information, Fig. S4a) even after 120 passages. The pgEpiSCs expressed pluripotent stem cell markers such as POU5F1, NANOG, and SOX2 (Fig. 2i), as well as pluripotent cell surface markers including SSEA1, SSEA4, TRA-1-81, and TRA-1-60 (Fig. 2j), during long-term in vitro maintenance. Importantly, embryonic body (EB) differentiation assays showed that pgEpiSCs can differentiate into the three germ layers upon removal of the inhibitors and cytokines from the medium (Fig. 2k). Directional induced differentiation assays showed that the pgEpiSCs could also differentiate into the expected three layers upon exposure to conditioned media (Fig. 2l). Teratoma formation assays confirmed that pgEpiSCs developed into the expected three germ layers in vivo (Fig. 2m, n). Supporting the high genetic integrity of our pgEpiSCs over long-term culture, a comparison of pgEpiSC lines derived from two different donors (full sibs) showed that very few of the detected genetic mutations (single nucleotide variations (SNVs): 1.10%; Indels: 3.13%) occurred after multiple passages during our culture (Supplementary information, Fig. S4b–g; see Materials and methods). We then randomly selected two cell lines for testing long-term maintenance ability, ultimately passaging them over 240 times without any indications of differentiation.

These results demonstrate the successful establishment of stable pluripotent stem cell lines from pig pregastrulation epiblasts.

### Inhibitor and growth factor requirements for long-term pgEpiSC maintenance

**Fig. 3:**
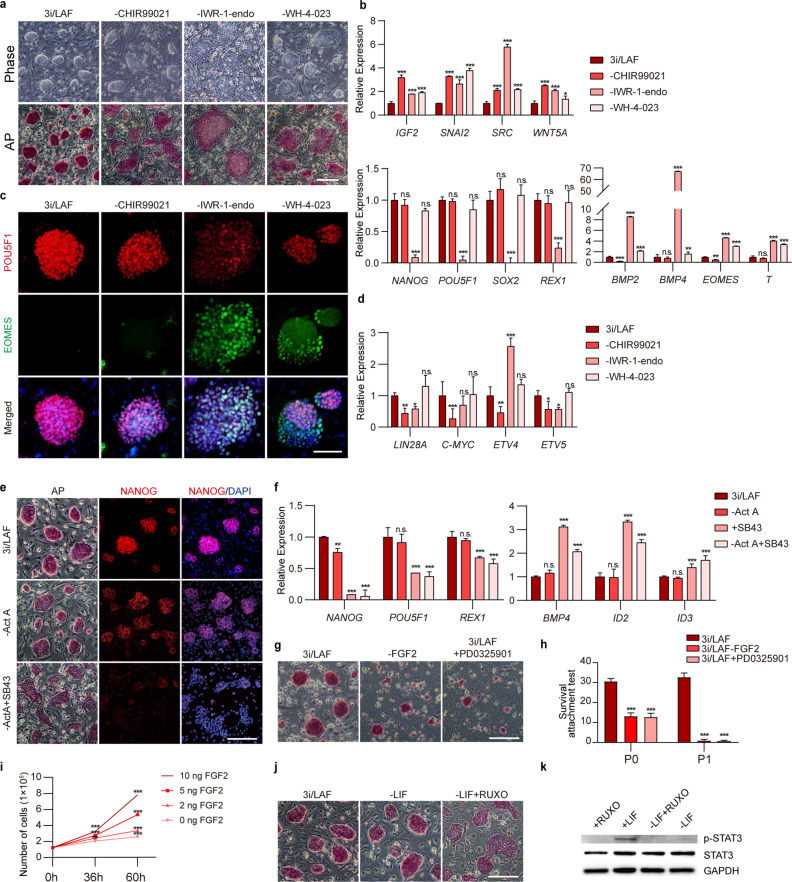
In vitro maintenance of pgEpiSCs requires 3i/LAF culture conditions. **a** Comparison of morphology and AP staining in pgEpiSCs cultured without CHIR99021, IWR-1-endo, and WH-4-023. Scale bar, 200 μm. **b** Quantification of mRNA expression of representative pluripotent marker genes involved in EMT (top), pluripotency (bottom left), and mesodermal differentiation (bottom right) by qRT-PCR. **c** Immunostaining for the pluripotency marker POU5F1 and mesoderm/endoderm progenitor marker EOMES. The nucleus is indicated by DAPI. Scale bar, 100 μm. **d** Quantification of mRNA expression of proliferation-associated genes by qRT-PCR. **e** AP staining and immunostaining of pgEpiSCs cultured without Activin A (Act A) or with added SB431542 (SB43) compared with control 3i/LAF-cultured pgEpiSCs. Scale bar, 200 μm. **f** Quantification of mRNA expression of pluripotent genes and BMP4 signaling-related genes in culture medium without Act A and/or with added SB43. **g** AP staining assay of pgEpiSCs cultured with or without FGF2 or with the ERK inhibitor PD0325901. Scale bar, 200 μm. **h** Cell survival and attachment test of pgEpiSCs in the presence of the indicated molecules. **i** Cell proliferation curve of pgEpiSCs treated with different concentrations of FGF2. **j** AP staining assay of pgEpiSCs cultured with or without LIF or with the JAK1/2 inhibitor ruxolitinib (RUXO). Scale bar, 200 μm. **k** Western blot analysis of LIF expression in pgEpiSCs during in vitro maintenance. For **b**, **d**, **f**, **h** and **i**, error bars indicate ± SD (*n* = 3, independent experiments); n.s., *P* ≥ 0.05; *P < 0.05, **P < 0.01, ***P < 0.001. For **a**, **c**, **e**, **g**, **j** and **k**, similar results were obtained in three independent experiments.

We tested the requirements of each factor in 3i/LAF culture medium for long-term in vitro maintenance of pgEpiSCs by removing the small-molecule inhibitors and cytokines individually from the culture medium. We found that removal of any of the three WNT signaling pathway-related inhibitors disrupted the desired domed colony morphology and weakened the alkaline phosphatase (AP) staining signal intensity (Fig. 3a); this also upregulated EMT-related genes, including *IGF2*, *SNAI2*, *SRC*, and *WNT5A* (Fig. 3b). In particular, the removal of IWR-1-endo led directly to the loss of clear boundaries of the pgEpiSC colonies and resulted in a significant downregulation of core pluripotency factors such as *NANOG*, *POU5F1*, *SOX2*, and *REX1* (Fig. 3b).

In addition, removal of IWR-1-endo or WH-4-023 resulted in mesodermal and endodermal differentiation of pgEpiSCs, marked by upregulation of gastrulation marker genes including *BMP2*, *BMP4*, *EOMES*, and *T* (Fig. 3b), and caused decreased or heterogeneous accumulation of the pluripotency factor POU5F1 while also promoting the expression of the mesoderm and endoderm progenitor marker EOMES^55^ (Fig. 3c). The removal of CHIR99021 downregulated the expression of cell proliferation-related genes such as *LIN28A, C-MYC, ETV4*, and *ETV5* (Fig. 3d), indicating impairment of pgEpiSC proliferation, which in turn suggests that the role of CHIR99021 is conserved in mouse, human, and pig PSCs.

Assays testing the removal of cytokines from 3i/LAF showed that removal of the TGF-β superfamily member Activin A led to reduced levels of the pluripotency marker NANOG (Fig. 3e, f). Further supporting the impact of Activin A in long-term culture of pgEpiSCs, addition of the general TGF-β inhibitor SB431542 into the culture medium (to avoid the impacts of factors secreted by feeder layer cells) led to irregular colony morphology and a strong reduction in the NANOG level (Fig. 3e, f). Notably, SB431542 addition also led to significant decreases in pluripotency markers (such as *POU5F1* and *REX1*) and significant increases in the levels of transcription factors (TFs) downstream of BMP4 and other BMPs; two such TFs are ID2 and ID3, which mediate the induction of the primitive streak during embryonic development^56^ (Fig. 3f).

When FGF2 was removed (and the ERK/MEK inhibitor PD0325901 was added), pgEpiSCs could not proliferate or be passaged normally (Fig. 3g, h). We also noted that a decreased FGF2 concentration significantly reduced the proliferation ability of the pgEpiSCs (Fig. 3i). Notably, we found that LIF was not essential for the maintenance of pgEpiSC colony morphology (Fig. 3j), but adding the JAK1/2 inhibitor ruxolitinib caused the colonies to flatten (Fig. 3j). Western blot analysis showed that phosphorylated STAT3 was detected only when LIF was present (Fig. 3k), indicating that pgEpiSCs can respond to the pluripotency-promoting effects^50^ of LIF stimulation.

### pgEpiSC transcriptome relatedness to pregastrulation epiblasts

**Fig. 4:**
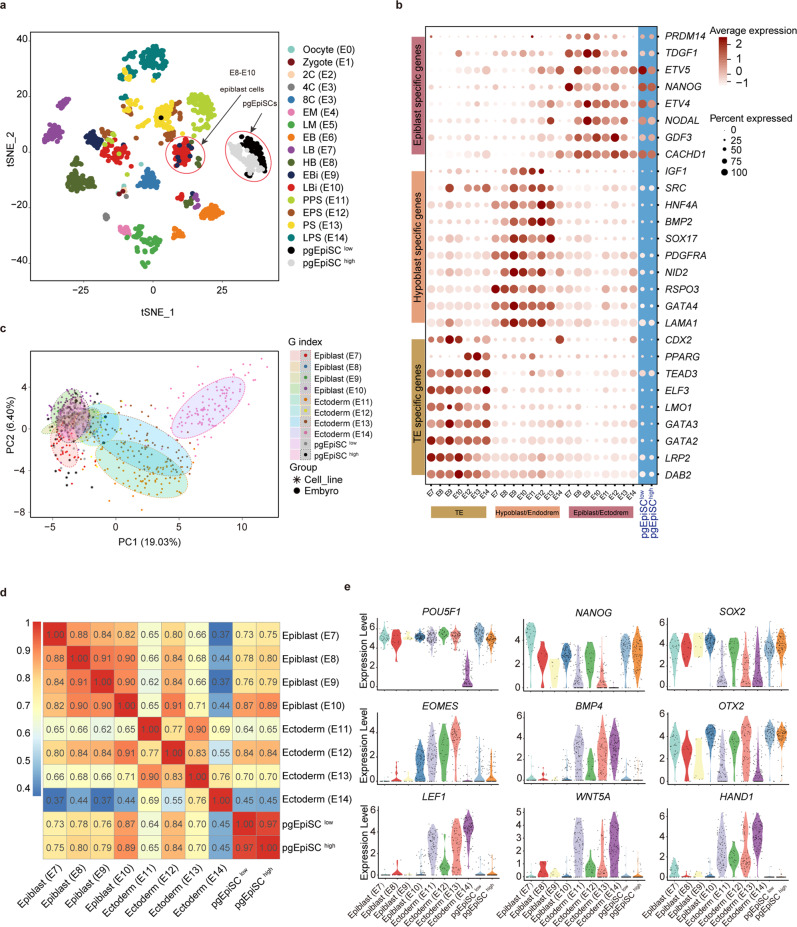
Characteristics of stable pgEpiSC lines. **a** t-SNE plot with scRNA-seq data from pig preimplantation embryonic cells (*n* = 1458) and pgEpiSCs (*n* = 196). Clusters are color-coded according to embryonic day and pgEpiSC passage number. The circled areas represent pregastrulation epiblasts and pgEpiSCs. **b** Dot plot for classical marker genes of TE, hypoblasts, and epiblasts during pig embryonic development. The color gradients represent average expression levels, and the sizes of the dots correspond to the percentages of cells that expressed the featured genes in TE, hypoblast, and epiblast cell populations. **c** PCA plot of pgEpiSCs and epiblast or ectoderm cells ranging from E7 to E14. Each dot represents a single cell in preimplantation embryonic cells, and the asterisks represent single cells in pgEpiSCs. Colors denote embryonic day and pgEpiSC passage number. **d** Spearman’s correlation coefficients based on the mean expression levels of uniquely expressed genes within each epiblast/ectoderm cell ranging from E7 to E14, which are related to pluripotency regulation and epithelial cell differentiation. **e** Violin plots showing the expression levels (log_2_(TPM/10 + 1)) of classical pluripotency genes in epiblasts/ectoderm from E7 to E14 and low- and high-generation pgEpiSCs based on scRNA-seq data. Each dot represents a single cell. See also Supplementary information, Fig. S5.

To investigate the transcriptomic features of the pgEpiSCs, we conducted scRNA-seq on pgEpiSCs sampled at passage 10 and passage 60 (referred to as low and high passages, respectively) and then compared the transcriptomes of pgEpiSCs to those of pig embryonic cells (from E0 to E14). t-SNE visualization showed that pgEpiSCs were clustered in a group independent of embryonic cells (from E0 to E14) (Fig. 4a). The expression levels of marker genes for lineage segregation indicated that epiblast-specific genes (*NANOG*, *TDGF1*, *ETV4*, *GDF3*, and *NODAL*) were highly and uniformly expressed in the pgEpiSCs (Fig. 4b and Supplementary information, Fig. S5a), suggesting that the pgEpiSCs maintain the transcriptomic properties of epiblast cells. Principal component analysis (PCA) of DEGs relevant with pluripotency and differentiation revealed that pgEpiSCs clustered closely to E10 epiblast cells (Fig. 4c and Supplementary information, Table S4a and Data S3). Pairwise correlation analysis demonstrated that the low- and high-passage pgEpiSCs exhibited remarkable consistency (*r* = 0.97, *P* < 2.2 × 10^–16^, Spearman’s rank correlation) and both displayed greater similarity to E10 epiblasts than to epiblast or ectoderm cells from other embryonic days (average *r* = 0.88, *P* < 2.2 × 10^–16^, Spearman’s rank correlation) (Fig. 4d). Furthermore, the expression levels of classical pluripotency and gastrulation markers in pgEpiSCs matched most closely with those of E10 epiblasts (Fig. 4e). All these results indicate that pgEpiSCs acquire transcriptome characteristics similar to those of their cells of origin, E10 epiblasts.

Next, we compared the bulk transcriptomes of pgEpiSCs generated here with previously reported transcriptomes from pig PSCs.^24–26,57–59^ The pgEpiSCs displayed greater similarity to pEPSCs^24^ and pESCs,^25^ although these three cell lines all exhibited distinct transcriptional alterations (Supplementary information, Fig. S5b, c). Further comparative analysis revealed that pgEpiSCs expressed high levels of canonical pluripotency markers (e.g., *NANOG, POU5F1, OTX2*) and maintained extremely low WNT pathway activity (Supplementary information, Fig. S5d, e). Functional enrichment analyses demonstrated that upregulated DEGs in our pgEpiSCs were enriched in terms such as translation, cell cycle, DNA replication, oxidative phosphorylation, etc. Compared with pgEpiSCs, DEGs upregulated in pEPSCs were enriched in terms such as Hedgehog signaling pathway, Wnt signaling pathway, and ECM-receptor interaction, and those in pESCs were enriched in the terms of Hippo signaling pathway, tube morphogenesis, epithelium and vasculature development (Supplementary information, Table S4d).

Furthermore, we carried out a comparative transcriptome analysis of the bulk RNA-seq data of pgEpiSCs and those of human naïve, conventional, and formative PSCs and mouse naïve, primed, and formative PSCs from previous reports.^10,60,61^ We found that pgEpiSCs were more similar to formative and primed (or conventional) PSCs than to naïve PSCs (Supplementary information, Fig. S5f). The pairwise correlation analysis recapitulated these findings (Supplementary information, Fig. S5g). Importantly, pgEpiSCs showed stronger expression of formative hPSC-specific genes than did conventional and naïve hPSCs (Supplementary information, Fig. S5h, i and Table S4b, c).

Taken together, these results support the successful establishment of pgEpiSC lines and suggest that pgEpiSCs show features of E10 pregastrulation epiblast cells and formative pluripotency.

### Dispersed chromatin architecture contributes to the pluripotency state of pgEpiSCs

**Fig. 5:**
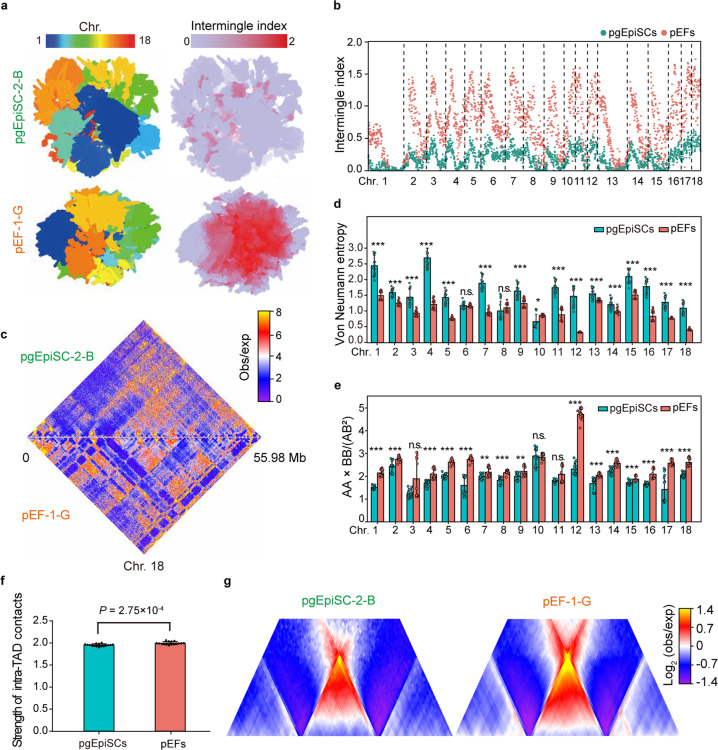
Comparison of nuclear architectures between pgEpiSCs and pEFs. **a** The 3D chromosome conformations were inferred for each Hi-C map at resolutions of 100 kb within a chromosome and 1 Mb between chromosomes. Example cross sections of pgEpiSCs-1-B and pEF-1-G nuclei, colored according to autosomes (left) or by the extent of the multichromosome intermingle index (reflecting the diversity of chromosomes as measured by Shannon’s index) (right).^65^ **b** Probability of extensive multichromosome intermingling (average of 16 Hi-C maps for each cell type) across 18 autosomes (smoothed by 1-Mb windows) in pgEpiSCs (green) and pEFs (red) at 100-kb resolution.^65^ **c** Example contact maps at 100-kb resolution for chromosome 18 in pgEpiSCs-1-B (upper half) and pEF-1-G (lower half). **d** The extent of disorder in chromatin structure (quantified by the Von Neumann Entropy (VNE))^64^ in each of 16 Hi-C maps for pgEpiSCs (green) and pEFs (red) at 100-kb resolution. **e** Compartmentalization strength (determined by the average contact enrichment within and between compartments (AA × BB/AB^2^)) in each of 16 Hi-C maps for pgEpiSCs (green) and pEFs (red) at 20-kb resolution.^66^ **f** The average strength of intra-TAD contacts (defined as the log_2_ ratio of intra- vs inter-TAD contacts)^66^ for each Hi-C map. **g** Examples of average TAD representation with intra- or inter-TAD contact in pgEpiSCs-1-B and pEF-1-G. For **d**, **e** and **f**, values are means ± SD. Statistical significance was calculated by the Wilcoxon rank-sum test (n.s., *P* ≥ 0.05; *P < 0.05, **P < 0.01, ***P < 0.001). See also Supplementary information, Fig. S6 and Data S1.

Pluripotency and self-renewal require that the PSC genome be in a highly plastic state, which supports entry into distinct differentiation trajectories.^62,63^ Using ultradeep in situ high-throughput chromatin conformation capture (Hi-C) sequencing, we reconstructed the three-dimensional (3D) structure of genomes for the pgEpiSCs and for pig embryonic fibroblasts (pEFs), ultimately obtaining maps with a maximum resolution of 300 bp after combining the data from 16 replicates (see Materials and methods; Supplementary information, Data S1-I and S3). Generally, we found that the pgEpiSCs had a higher extent of spatial fluctuations in their chromatin than the pEFs did (reflected by the reduced extent of chromosome intermingling in the pgEpiSCs compared to the pEFs: 0.18/0.71, *P* < 2.2 × 10^–16^, Wilcoxon rank-sum test) (Fig. 5a, b). The more disordered (and permissive) chromatin in the pgEpiSCs was also evident based on its high-entropy status (1.57/1.00, *P* = 5.63 × 10^–4^, Wilcoxon rank-sum test) (Fig. 5c, d), consistent with previous studies in humans and mice.^64,65^ Fundamentally, the characteristically loose regulatory architecture we detected in this ultradeep in situ Hi-C analysis helps explain the observed capacity of our pgEpiSCs to differentiate towards multiple cell identities.

Next, analysis at the subchromosome scale revealed that the compartmentalization in pgEpiSCs was much reduced compared to that in pEFs (1.89/2.45, *P* = 5.03 × 10^–4^, Wilcoxon rank-sum test) (Fig. 5e and Supplementary information, Data S1-I). This finding supports a trend reported in previous studies:^66,67^ compared to PSCs, specialized cell lineages typically exhibited global shifts in compartment dynamics and a concomitant increase in the number of interactions between inactive (and less accessible) heterochromatin regions (Supplementary information, Fig. S6a). Specifically, we defined the set of pgEpiSC-restricted compartment A regions (280.88 Mb) and subsequently found that the genes (2817) located within them tended to show increased expression compared to the same genes within compartment B regions in the pEFs (median fold change = 1.48, *P* < 2.20 × 10^–16^, Wilcoxon rank-sum test) (Supplementary information, Fig. S6b).

Intriguingly, the core pluripotency regulator genes *NANOG* and *SOX2* were among these differentially compartmentalized and expressed genes (Supplementary information, Fig. S6c), and functional enrichment analysis highlighted putative functions related to “cell fate commitment” and “regulation of stem cell population maintenance” (Supplementary information, Fig. S6d)^47^. In contrast, the 1164 genes located within the pgEpiSC-restricted compartment B regions (219.32 Mb) were generally downregulated compared to when they were in the compartment A regions of the pEFs (median fold change = 0.59, *P* < 2.20 × 10^–16^, Wilcoxon rank-sum test) (Supplementary information, Fig. S6b). *CHI3L1*, a known marker of fibroproliferative responses, was among this set of genes^68^ (Supplementary information, Fig. S6c), and functional enrichment analysis highlighted putative functions related to the terminal differentiation of the PEFs (e.g., “extracellular matrix organization” and “tissue morphogenesis”) (Supplementary information, Fig. S6d).

We further performed an ATAC-seq assay to measure the differences in local accessibility between the genomes of pgEpiSCs and pEFs. As expected, we observed that the pgEpiSC-specific peaks mainly occurred in their restricted compartment A regions (Supplementary information, Fig. S7a–h), which are enriched in motifs corresponding to common pluripotent TFs (typically POU5F1, SOX2, KLF5, and POU3F1) (Supplementary information, Fig. S7i).^69^ Thus, beyond offering evidence that epigenetic chromatin state-mediated compartment activation contributes to establishing and maintaining the pluripotent state, these results also directly implicate multiple loci with distinct compartmentalization and accessibility between PSCs and terminal lineage cells in cell identity.

At a finer scale, we partitioned the genome into topologically associating domains (TADs) at 20-kb resolution^70^ (Supplementary information, Fig. S6e). Although the TAD boundaries were mostly shared between the pgEpiSCs and the pEFs (96% common), the average strength of intra-TAD contacts was weaker in pgEpiSCs than in pEFs (1.95/2.00, *P* = 2.75 × 10^–4^, Wilcoxon rank-sum test) (Fig. 5f, g and Supplementary information, Fig. S6f–h). This finding further supports the hypothesis that genome organization is highly plastic (reduced self-interaction) in pluripotent pgEpiSCs compared to terminally differentiated pEFs.^66^ The comparison of publicly available Hi-C data between ESCs of humans^67,71,72^ and mice^66,73^ against specialized fibroblasts recapitulated these multiscale levels of evidence for more dispersed chromatin architecture in the nucleus of the pgEpiSCs (Supplementary information, Data S1-II, III).

### The spatial regulatory circuitry of transcription underpins pgEpiSC pluripotency

**Fig. 6:**
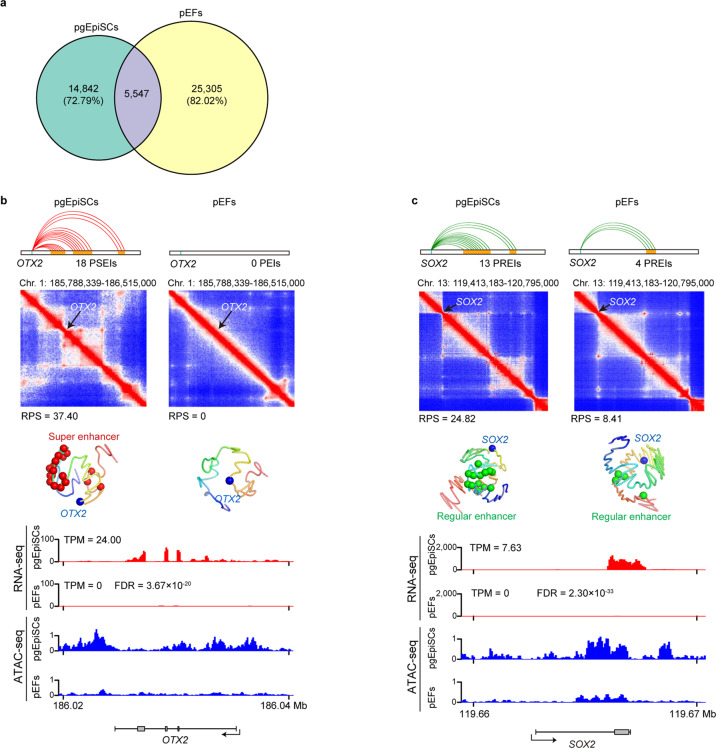
Comparison of spatial regulatory circuitry of the transcription between pgEpiSCs and pEFs. **a** Overlap of PEIs. Only 27.21% and 17.98% of PEIs in the pgEpiSCs and pEFs appeared in the pEFs and pgEpiSCs, respectively. **b**, **c** Schematic representation of the PEIs of *OTX2* (**b**) and *SOX2* (**c**). Top panel, Hi-C map of the regions around the center of the gene’s transcriptional start site (± 250 kb). Middle panel, 3D model of the promoter (blue sphere) and its enhancers (red and green spheres represent super- and regular enhancers, respectively). Bottom panel, gene track of RNA-seq and ATAC-seq profiles around the gene locus (± 5 kb). The Benjamini–Hochberg-adjusted FDRs were calculated. See also Supplementary information, Figs. S7, S8 and Data S2.

We investigated how the comparatively loose heterochromatin regulatory architecture of pgEpiSCs (Fig. 5) may affect the specification of active transcriptional programs for pluripotency. We found fewer promoter–enhancer interactions (PEIs) (20,389 enhancers assigned to 6498 promoters) in the pgEpiSCs than in the pEFs (30,852 enhancers assigned to 7823 promoters) (Supplementary information, Data S2-Ia–e and S3). To elucidate how this observed extensive rewiring of PEIs may contribute to the transcriptomic divergence between the pgEpiSCs and the pEFs (only 5547 PEIs are shared) (Fig. 6a), we calculated a regulatory potential score (RPS), a spatial proximity-based index representing the combined regulatory effects of multiple enhancers for a given gene, for each promoter (Supplementary information, Data S2-If, g and S3).^74–76^ We identified 875 genes with covariation between RPS and gene expression; genes having higher RPS values were generally upregulated in the pgEpiSCs compared to the pEFs (log_2_-fold change (FC) > 1, FDR < 0.05) (Supplementary information, Table S5). This set of genes was enriched for annotations related to “cell division” (Supplementary information, Fig. S8a).^47^ Moreover, 75 of these genes were strongly expressed in the pgEpiSCs (TPM > 5 compared to TPM < 0.5 in pEFs), and many of them are known to function in preserving pluripotency (typically, “signaling pathways regulating pluripotency of stem cells”) (Supplementary information, Fig. S8b). We detected that *OTX2* (as well as *LIN28A*, *NANOG*, *PRDM14*, *SALL4*, *UTF1*, and *ZFP42*) and showed specific enhancer interactions in the pgEpiSCs, while enhancer interactions were depleted in the pEFs (Fig. 6b and Supplementary information, Data S2-IIa–f), whereas *SOX2* (as well as *CDH1*, *DNMT3B*, and *LEFTY2*) had more and spatially closer enhancers in the pgEpiSCs than in the pEFs (Fig. 6c and Supplementary information, Data S2-IIg–i).

We next investigated interactions between promoters, which conceptually represent an additional layer of 3D genome organization with the potential to influence gene expression.^77^ Consistent with previous results in mice^77,78^ and humans,^79^ genes with relatively strong expression exhibit an elevated extent of shared contacts among themselves (i.e., this group of genes has more intrachromosomal promoter–promoter contacts) (Supplementary information, Fig. S8c and Data S3). Notably, an in silico analysis^80^ supported likely transcriptional consequences of these increased interactions:^62,78^ common pluripotency TFs (typically MYC, KLF5, POU5F1, and SOX2) were predicted to preferentially bind at the genomic loci in pgEpiSCs over pEFs for 1254 such strongly expressed genes (Supplementary information, Fig. S8c). Thus, our ultradeep in situ Hi-C sequencing data for the pgEpiSCs and the pEFs enabled informative, multiscale analyses of 3D genome organization and transcriptional regulation. Beyond indicating a clear impact of 3D-spatial associations on the transcription of genes known to function in maintaining pluripotency,^81^ our results implicated many pluripotency-related candidate regions and loci that can be pursued in future hypothesis-driven basic investigations of cell differentiation.

### pgEpiSCs show limitations in chimeric embryo development

To test whether pgEpiSCs can be incorporated into embryos to form chimeras, we injected GFP-labeled pgEpiSCs into the cavities of E5 early blastulae and monitored the presence of pgEpiSCs in the ICMs of the injected pig blastocysts (Supplementary information, Fig. S9a, b). GFP-positive cells were detected in 47.62% ± 15.17% of 268 blastocysts produced by the injection assay. Further immunostaining assays indicated that only 20% of the injected embryos showed incorporation of GFP-positive cells into the ICM (Supplementary information, Fig. S9c, d). To further test pgEpiSC development in chimeric embryos, we transplanted 1031 E6 chimeric embryos into six sow uteruses at the appropriate stage of the oestrus cycle. A total of 135 developed embryos at E10 and 26 embryos at E21–E23 were obtained for testing. Unfortunately, no GFP-positive cell signals were observed in the embryos (Supplementary information, Fig. S9e). We speculated that the pgEpiSC proliferation rate could not adapt to the rapid proliferation of epiblasts in E7–E10 blastocysts; this issue requires further study in the future.

### Piglets cloned from pgEpiSCs after multiple successive rounds of gene editing

**Fig. 7:**
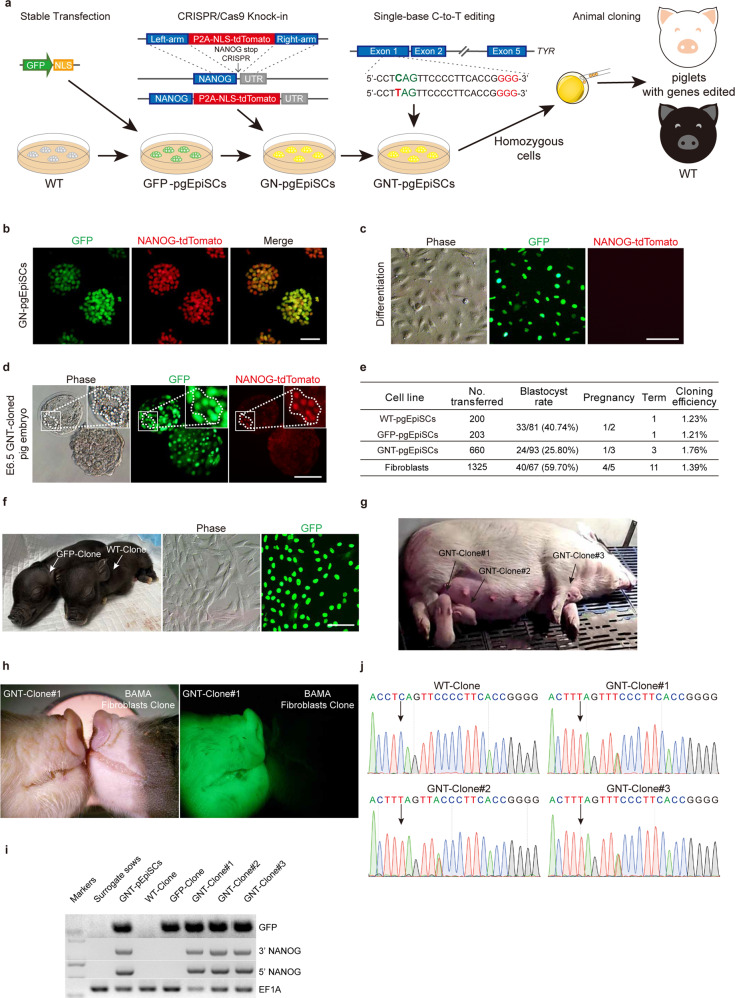
pgEpiSCs tolerate multiple successive rounds of gene editing and can generate cloned gene-edited live piglets. **a** Schematic diagram of the sequential editing strategy for multiple genes and generation of cloned piglets using pgEpiSCs as donors by cell nuclear transfer assay. **b** The expression of the NANOG-tdTomato knock-in reporter in GFP-labeled pgEpiSCs. Scale bar, 100 μm. **c** Loss of tdTomato expression after differentiation of GN-pgEpiSCs. Scale bar, 200 μm. **d** NANOG-tdTomato knock-in reporter indicates the localization of NANOG-positive cells in cloned embryos using GNT-pgEpiSCs as donor cells. Scale bar, 100 μm. **e** Summary of pgEpiSC nuclear transfer experiments. The blastocyst rate was calculated using the embryos reserved before transplantation. Cloning efficiency was obtained by calculating the number of born piglets/(number of embryos transferred × blastocyst rate). The fibroblasts were from Bama pigs. **f** Fibroblasts from the ears of WT pgEpiSC-cloned piglets and GFP-pgEpiSC-cloned piglets. **g** Three GNT-pgEpiSC-cloned piglets and their surrogate mother. **h** A representative GNT-pgEpiSC-cloned piglet shows GFP fluorescence, in contrast to a WT piglet cloned from Bama pig fibroblasts. **i** Gel photos of PCR tests for NANOG-tdTomato knock-in and GFP transfection, with *EF1A* as the control. **j** DNA sequence analysis of the *TYR* gene C-to-T mutation site for WT pgEpiSC and three GNT-pgEpiSC-cloned piglets. See also Supplementary information, Fig. S10.

Large animal models with complex polygenic modifications are considered important in biological research and biomedicine.^21^ One of the major limitations in the current use of pig somatic cell nuclear transfer is that somatic donor cells can typically only support a single round of genome editing.^19^ To test whether pgEpiSCs could tolerate successive rounds of genome editing, we conducted experiments investigating multiple forms of genomic manipulation (Fig. 7a).

First, we obtained pgEpiSCs that were stably transfected with a GFP-NLS reporter cassette, and flow cytometry indicated that the GFP-positive cell rate was 21.27% (Supplementary information, Fig. S10a, b). Second, using these GFP-positive cells, we performed CRISPR/Cas9-mediated knock-in, specifically inserting a tdTomato reporter cassette into the *NANOG* locus at a position immediately preceding the native stop codon, generating GFP-NANOG-tdTomato pgEpiSCs (GN-pgEpiSCs) (Supplementary information, Fig. S10c). GN-pgEpiSC colonies were selected based on NANOG-tdTomato fluorescence and then re-expanded in 3i/LAF medium (Fig. 7b). Consistent with the known status of NANOG (marker of pluripotency), no tdTomato reporter fluorescence was detected after experimentally initiating differentiation of the knock-in-edited pgEpiSCs (Fig. 7c). For the third and final examined genome modification, we performed C-to-T conversion with cytosine base editors (CBEs)^82^ at the stop codon of the *TYR* loci, introducing a mutation known to cause albinism in the gene responsible for pig coat color.^83,84^ Sequencing-based analysis of 99 colonies indicated that 24.24% (24/99) of them were heterozygous, and 3.03% (3/99) were homozygous for the C-to-T base edit at *TYR* in the GN-pgEpiSCs background (termed GNT-pgEpiSCs) (Supplementary information, Fig. S10d, e). These results indicated that pgEpiSCs can tolerate successive rounds of genome modification, including traditional transgenic insertion, precision knock-in with CRISPR/Cas9 and single-base conversion editing using CBEs.

We then performed cell nuclear transfer assays and examined the developmental potential of the cloned embryos, specifically, using wild-type (WT) pgEpiSCs, GFP-pgEpiSCs, and GNT-pgEpiSCs as nuclear donor cells. As expected, NANOG-tdTomato fluorescence was detected in only a small fraction of the ICM cells of the cloned blastocysts when GNT-pgEpiSCs were used as donors (Fig. 7d), which is consistent with the expression patterns of *NANOG* in natural embryos revealed by scRNA-seq data (Fig. 1d). We also assessed the in vivo developmental potential of the cloned embryos by mixed transfer of 200 and 203 cloned embryos from WT pgEpiSCs and GFP-pgEpiSCs donors into the uteri of two recipient females in the same oestrus and 660 cloned embryos from GNT-pgEpiSCs donors into the uteri of three recipient females (Fig. 7e). We ultimately obtained one cloned piglet from WT pgEpiSCs, one from GFP-pgEpiSCs, and three from GNT-pgEpiSCs (Fig. 7f, g). The cloning efficiencies of the gene-edited pgEpiSCs were similar to those of the WT pgEpiSCs and comparable to those of fibroblasts (Fig. 7e). Fluorescence, PCR and sequencing assays confirmed the cloned piglets’ origins (Fig. 7h–j). Importantly, GNT-pgEpiSC-cloned piglets showed the expected albino white coat color phenotype (Supplementary information, Fig. S10f). These results indicated that pgEpiSCs tolerate successive rounds of multiple gene editing and can be used successfully to generate complex pig models.

## Discussion

Here, based on a scRNA-seq analysis of pluripotency changes during the development of pig E0–E14 preimplantation embryos, we developed 3i/LAF culture medium, with which we efficiently generated stable pgEpiSCs from E10 pregastrulation epiblasts. These pgEpiSCs express pluripotency markers, can differentiate into three germ layers, have highly plastic chromatin architecture, and tolerate long-term passages while retaining normal karyotypes. In addition, we found that these cells can tolerate at least three rounds of successive gene editing, including genomic insertion of a GFP-NLS reporter cassette, CRISPR/Cas9-mediated knock-in for insertion of a tdTomato reporter, and genome modification of C-to-T conversion using CBEs. Finally, we used pgEpiSCs from multiple rounds of editing as donor cells for cell nuclear transfer and successfully produced live cloned piglets harboring multiple gene edits.

The establishment of stable pig PSCs has been much more difficult in pigs than in mice or humans. Pig PSCs have been derived from preimplantation embryo cells, including morulae, the ICMs of blastocysts, and epiblasts/ectoderms in E8–E12, based on the culture conditions for mouse and human stem cells.^23,26^ However, these cells have only partial features for meeting the criteria established to define suitable mouse PSCs.^28,85^ We hypothesized that the failure to establish stable pig embryonic stem cell lines may be related to a lack of understanding of some pig-specific regulatory mechanisms that function in early embryonic development. The available transcriptome datasets for pig embryonic cells are limited by the absence of some embryonic stages and insufficient cell numbers, so we performed scRNA-seq of all stages of preimplantation embryos (E0–E14) to generate a comprehensive molecular profile of early pig embryonic development and pluripotency changes. We accurately defined the time points of the three lineage differentiation events of pig preimplantation embryos and the corresponding embryo morphology (Fig. 1c, d), indicating that the concurrent establishment of epiblasts, hypoblasts, and TEs during the early blastocyst stage is conserved among humans, mice, and pigs.^1,34^ We identified and traced the changes in naïve, formative and primed pluripotency states during pig epiblast development and found that pig epiblasts lost naïve pluripotency features quickly and maintained a relatively long formative pluripotency state. These analyses enabled the generation of pgEpiSC lines from E8–E10 pregastrulation epiblasts.

We also compared the 3D structures of the genomes of the pgEpiSCs and specialized pEFs at multiple hierarchical scales, providing a rich resource for studying dynamic chromatin interactions involving different processes affecting, for example, cell fate and differentiation. Consistent with previous findings in mice and humans,^64–67,78^ we observed that dispersed chromatin architecture contributes to the pluripotent state of pgEpiSCs. Compared to pEFs (which are terminally differentiated), pgEpiSCs are characterized by a distinct higher-order global chromatin structure, with relatively more disordered chromatin, reduced compartmentalization, and reduced self-interaction within TADs. The highly plastic state of genome organization in pluripotent pgEpiSCs is likely a prerequisite for their entry into any differentiation pathway.^63,81^

Our ultradeep in situ Hi-C sequencing dataset allowed us to determine which active transcriptional programs underpin pgEpiSC pluripotency. We found that PEIs and spatially associating clusters of target genes induced by common TFs are highly dynamic and are often established concomitantly with gene expression,^77,79^ which contributes to the maintenance of pluripotency and the determination of differentiation properties. Our results also implicated many pluripotency-related regulatory DNA elements (e.g., enhancers) that can be studied further in future hypothesis-driven basic investigations of cell differentiation.^62,75,76^ Our ultradeep in situ Hi-C sequencing dataset can serve as a valuable tool for the research community to better understand the gene regulatory networks controlling pluripotency and differentiation of pigs and other mammalian pluripotent stem cells.

The pig is an attractive animal model with prospects for broad medical applications.^17,19^ Genetically modified pig models are traditionally produced using a combination of gene editing and somatic cell nuclear transfer. However, it is difficult to edit somatic cells multiple times owing to their limited proliferation ability and very low homologous recombination frequency.^86^ Therefore, producing multigene-edited cloned pigs often requires multiple rounds of re-cloning,^18^ which greatly increases the time and cost of such efforts. Thus, stable pig PSCs — combined with powerful new genome engineering tools — have the potential to contribute to the ongoing revolutions in regenerative biomedical research and animal breeding. To the best of our knowledge, our demonstration that pgEpiSCs harboring edits from three rounds of gene editing can be used as donor cells represents the first report showing the successful cloning of live multigene-edited piglets. This new capacity seems extremely likely to promote the development of complex model pigs with polygenic diseases or traits and to support biotechnological applications. Owing to their capacity for long-term expansion and differentiation potential, pgEpiSCs have further application prospects, for example, as seed cells for generating cultured meats.

## Materials and methods

### Animal treatment and ethics statements

All mouse experiments and pig procedures were approved in advance by the Institutional Animal Care and Use Committee of China Agricultural University, the Institutional Animal Care and Use Committee of Yunnan Agricultural University, the Institutional Animal Care and Use Committee of the Chinese Academy of Sciences, and the Institutional Animal Care and Use Committee of Northeast Agricultural University.

### Mice

CD-1^®^ (ICR) IGS and BALB/c nude mice were purchased from Beijing Vital River Laboratory Animal Technology Co., Ltd. (Beijing, China) and were used for isolation of mouse embryonic fibroblasts (MEFs) and the teratoma formation test. The feeder cells for pgEpiSCs were prepared from MEFs treated with mitomycin C (Selleckchem, S8146).

### Pigs

For pig embryonic single-cell collection and single-cell transcriptome analysis, BAMA pig oocytes, zygotes, and embryos at E2–E14 were used. All pigs were naturally in oestrus and mating. Embryonic day n (E(n)) embryos were obtained n days after mating. For pgEpiSC derivation, Nongda Xiang pig conceptuses at embryonic days E5–E12 were used. For the production of cloned piglets, BAMA or DLY oestrous sows were used as surrogate mothers.

### Collection of preimplantation pig embryos and isolation of embryonic single cells

For oocyte collection, oocytes were aspirated from the follicle using a syringe with follicular fluid and cleaned with embryo washing buffer (DPBS + 2% FBS). Well-developed oocytes with multiple layers of cumulus cells were screened for collection. Hyaluronidase (Sigma, H3506) was used to remove cumulus cells, and pronase (Sigma, 10165921001) was used to remove the zona pellucidae.

Zygotes and 2-cell stage and 4–8-cell stage embryos were flushed out from the fallopian tube and collected. The mesosalpinx was cut to straighten the fallopian tube, and 25–50 mL of embryo washing buffer was injected through the tubal umbrella and discharged from the uterine horn incision. The embryos were removed from the embryo washing buffer with an embryo transfer pipette and washed three times. The embryos were treated with pronase for 10–15 s until the zona pellucidae became thin and soft and then were transferred to collection washing buffer (DPBS + 0.1% BSA) for cleaning. The blastomeres were mechanically separated and transferred to lysis buffer.

For collection of E4 early morula stage embryos to E6 early blastula embryos, the embryos were washed out of the womb. The mesometrium was cut along the direction of the uterus to straighten the uterus. One end was cut from the junction between the fallopian tube and the uterine horn, and the other end was cut from the junction between the cervix and the uterus; 50–100 mL embryo washing buffer was injected from the uterine horn, and the embryo was carried out of the end of the uterus along with the liquid flow. The zona pellucidae was removed as described above, and then single cells were isolated from the embryos by enzyme treatment using TrypLE™ Express (Gibco, 12605010) at 37 °C for 1–3 min.

For collection of E7 late blastula embryos, the embryos were obtained from the uterus, and then the zona pellucidae was perforated with a laser membrane rupture device. TrypLE™ Express (Gibco, 12605010) was then injected into the inner blastocyst cavity to dissociate epiblasts and hypoblasts and release them from the zona pellucida; epiblasts/hypoblasts and TE cells were digested separately for single cell collection.

For collection of E8–E14 embryos, embryos were obtained from the uterus. Single cells from the embryos were obtained by enzyme treatment and mechanical separation.

Because of the random selection of cells, the TE cells of the E11 embryos and the extracellular endoderm cells of E13–E14 embryos were not obtained. This should not affect the conclusions from the analysis about tracing pluripotency during epiblast development.

### Pig pgEpiSC culture medium

Pig pgEpiSCs were cultured in 3i/LAF culture medium under 20% O_2_ and 5% CO_2_ at 37 °C. The basal medium (BM) of 3i/LAF was optimized with reference to the BM of the LCDM system^53^ and contained the following components per 500 mL: 227.5 mL DMEM/F12 (Thermo Fisher Scientific, 10565-018), 227.5 mL neurobasal (Thermo Fisher Scientific, 21103-049), 2.5 mL N2 supplement (Thermo Fisher Scientific, 17502-048), 5 mL B27 supplement (Thermo Fisher Scientific, 12587-010), 0.5% GlutaMAX (Thermo Fisher Scientific, 35050-061), 1% nonessential amino acids (Thermo Fisher Scientific, 11140-050), 0.1 mM β-mercaptoethanol (Thermo Fisher Scientific, 21985-023), 1% penicillin–streptomycin (Thermo Fisher Scientific, 15140-122), 5% knockout serum replacement (KOSR, Thermo Fisher Scientific, A3181502, optional), and 50 μg/mL ascorbic acid (Sigma–Aldrich, A4544). To prepare the 3i/LAF medium, small molecules and cytokines were added to BM to the following final concentrations: CHIR99021 (1 μM, Selleckchem, S1263), IWR-1-endo (2.5 μM, Selleckchem, S7086), WH-4-023 (1 μM, Selleckchem, S7565), recombinant human LIF (10 ng/mL, PeproTech, 300-05), recombinant human activin A (25 ng/mL, PeproTech, 120-14E), and recombinant human FGF-basic (154 aa) (10 ng/mL, PeproTech, 100-18B). To promote pgEpiSC proliferation, the ROCK inhibitor Y27632 was added (10 μM for passaging, 2 μM for maintenance; Selleckchem, S1049). pgEpiSCs were cultured on mitomycin C (Selleckchem, S8146)-treated mouse embryonic fibroblast (MEF) feeder cells (5 × 10^4^ cells per cm^2^). The culture medium was exchanged every 12 h with fresh 3i/LAF medium. The following three points are important for maintaining pgEpiSCs in an undifferentiated state: (a) the newly prepared 3i/LAF medium should be stored at 4 °C for no more than a week and should not be frozen; (b) the passage density must be appropriate: the seeding density of pgEpiSCs was approximately 3–5 × 10^4^ cells/cm^2^; and (c) fresh feeder cells and proper density (3–4 × 10^4^ cells/cm^2^) must be ensured. Pig pgEpiSCs were passaged as single cells by Accutase cell dissociation reagent (Gibco, A11105-01) every 2–3 days at a ratio from 1:3 to 1:5. The exact number of passaged days and proportions should be adjusted according to the actual situation.

### Derivation of pgEpiSCs from E10 pig epiblasts

For derivation of pig pgEpiSCs from E10 embryonic epiblasts, the hypoblast and TE cells were removed using a mechanical method, and the embryonic epiblast was treated with TrypLE™ Express (Gibco, 12605010) for 3 min and dispersed into small cell masses. The cell masses were seeded onto MEF feeders in 12-well cell culture dishes (Thermo Fisher Scientific, 150628) with 750 μL 3i/LAF medium containing 10 μM Y27632 (Selleckchem, S1049). After 12 h, 750 μL fresh 3i/LAF medium was added without changing the culture medium. After 24 h, the culture medium was exchanged every 12 h with fresh 3i/LAF medium. Outgrowths with domed colony morphology were subcultured with Accutase cell dissociation reagent (Gibco, A11105-01) treatment.

### Single-cell RNA library preparation and sequencing

A scRNA-seq library was prepared by a modified Smart-seq2 protocol as described in previous studies.^39,40^ Briefly, single embryonic cells were transferred into prepared lysis buffer containing an 8-bp barcode. Then, first-strand cDNA was reverse-synthesized and amplified in a reverse transcription (RT) mixture containing 4 U RNase inhibitor, 100 U SuperScript II reverse transcriptase (Invitrogen, 18064071), 1 mM dNTPs (TAKARA, 4019), 60 mM MgCl_2_, and 3 µM RT primer with 10 µM TSO primer. After PCR amplification, the product was purified using 0.8× AMPure XP beads (Beckman, A63882). Subsequently, biotin PCR was carried out for enrichment. Finally, the scRNA-seq library was constructed according to the directions for KAPA Hyper Prep Kits with PCR Library Amplification/Illumina series (KAPA, KK8054). High-quality libraries were sequenced with 150-bp paired-end reads on an Illumina HiSeq Xten (Novogene). The primers used in these experiments are listed in Supplementary information, Table S6 (Key Resources Table).

### Cell growth curve and population doubling time

Pig EpiSCs were cultured in 12-well plates. Triplicate samples of cells were seeded at a density of 2 × 10^5^ cells per well. The cell numbers were counted every 12 h. For each time point, cells were digested and counted using a Luna™ Automated Cell Counter. Those counts were averaged three times and plotted. The cell doubling time was calculated as follows: Doubling time (DT) = 24 × [lg2/(lg*N*_*t*_ − lg*N*_0_)], where 24 is the cell culture time (h); *N*_*t*_ is the number of cells cultured at 48 h; and *N*_0_ is the number of cells recorded at 24 h.

### Analysis of single-cell cloning efficiency

Cells were dissociated by Accutase (Gibco, A11105-01), counted using a haemocytometer, and plated onto preseeded 6-well plate feeders at a density of 100, 200, and 1000 cells per well in triplicate under pgEpiSC culture conditions. The colonies were counted 6 days later using AP staining, and colony formation efficiency was evaluated as a percentage of colony number per number of cells seeded.

### Karyotype analyses

Before karyotype analysis, 1% KaryoMAX Colcemid Solution (Gibco, 15212012) was added to the pgEpiSC culture medium, and the cells were incubated for 1 h. The pgEpiSCs were digested into single cells by TrypLE™ Express (Gibco, 12605010) and collected by centrifugation. pgEpiSCs were resuspended with 0.075 M KCl (Sigma, P5405) hypotonic solution and incubated at 37 °C for 15 min. Then, pgEpiSCs were fixed with methanol and acetic acid at a ratio of 3:1, and this process was repeated three times. The pgEpiSC suspension was dropped onto a precooled slide, dried thoroughly at room temperature, and then dyed with 10% Giemsa Stain Solution (Sangon, E607314-0001) for 30 min. For each cell line, more than 30 cells at metaphase were examined.

### Whole-genome sequencing

Total DNA of pgEpiSCs was extracted using a TIANamp Genomic DNA Kit (TIANGEN, DP304). After DNA extraction, 1 µg genomic DNA was randomly fragmented by Covaris, and 200–400 bp fragments were selected using an Agencourt AMPure XP-Medium kit (BERCKMAN COULTER, A63880). Selected fragments were end-repaired and 3’ adenylated, and then the adaptors were ligated to the ends. The products were amplified by PCR, and then the purified PCR products were heat denatured to single strands and circularized by the splint oligo sequence. The single-strand circular DNAs were formatted as the final library and verified by quality control. The final verified libraries were sequenced by BGISEQ-500.

### AP staining

AP staining of pgEpiSCs was based on the Alkaline Phosphatase Detection Kit (Millipore, SCR004). Specific experimental steps followed the kit instructions.

### Immunofluorescence analysis

Cells were washed with DPBS (Gibco, C14190500BT), fixed with 4% paraformaldehyde at room temperature for 30 min, washed with DPBS again, permeabilized in 0.1% Triton X-100 for 20 min, and blocked with 3% BSA for 1 h. The cells were incubated with primary antibodies diluted with 3% BSA at 4 °C overnight. Cells were then washed with wash buffer (DPBS containing 0.1% Triton X-100 and 0.1% Tween 20) for 5 min three times. Secondary antibodies were diluted and incubated with wash buffer at room temperature for 1 h and then washed with wash buffer three times for 5 min, and the nuclei were stained with DAPI (Roche Life Science, 10236276001) for 3 min. The antibodies used are listed in Supplementary information, Table S6 (Key Resources Table).

### Embryoid body differentiation

pgEpiSCs were dissociated by Accutase (Gibco, A11105-01), separated from the feeder cells using a differential attachment method, and cultured for 5–7 days on 35-mm low-attachment plates in DMEM (Gibco, 11960-044) supplemented with 10% FBS (Gibco, 16000-044), 1% penicillin–streptomycin (Thermo Fisher Scientific, 15140-122), and 1% GlutaMAX (Thermo Fisher Scientific, 35050-061) on a horizontal shaker at 50 rpm. Regular spherical EBs were selected and plated on gelatine-coated plates for over 1 week in the same medium, fixed and detected using the same methods as for immunofluorescence.

### Directional induced differentiation

For neural induction, the 3i/LAF culture medium was replaced with neural induction medium I (2.5 μM IWR-1-endo, 5 μM SB431542, and 10 ng/mL FGF2 in BM) on day 2 after pgEpiSC passage. After culturing for 2 more days, the medium was changed to neural induction medium II (4 μM RA, 10 ng/mL FGF2, and 20 ng/mL Noggin in BM), and immunostaining was performed another 2 days later.

For endoderm induction, the 3i/LAF culture medium was changed to BM with 10 ng/mL BMP4, 5 μM SB431542, and 10 ng/mL FGF2 two days after pgEpiSC passage, and then immunostaining was performed another 2 days later.

For mesoderm induction, the 3i/LAF culture medium was changed to mesoderm induction medium I (10 ng/mL BMP4, 50 ng/mL Activin A, and 20 ng/mL FGF2 in BM) 2 days after pgEpiSC passage. Subsequently, medium I was changed to mesoderm induction medium II (3 μM IWR-1-endo, 5 μM CHIR99021, and 20 ng/mL FGF2 in BM), and immunostaining was performed another 2 days later.

### Teratoma formation

For the teratoma formation assay, approximately 1 × 10^7^ cells of dissociated pgEpiSCs were collected by centrifugation at 1000 rpm for 5 min and subcutaneously injected into the posterior neck of BALB/c nude mice. Teratomas were seen after 4–5 weeks of growth.

### H&E analysis

Teratomas were collected subcutaneously from nude mice, washed twice in PBS and fixed with 4% PFA for 2 days at 4 °C. Teratoma tissues were dehydrated with an alcohol gradient (70%, 80%, 90%, 95%, and 100% for 1 h each), transferred into xylene and embedded in paraffin. Samples were sliced to 5-μm thickness, deparaffinized in xylene and rehydrated with decreasing concentrations of ethanol. Samples were then stained with haematoxylin (Sigma–Aldrich, MHS16) and eosin (Sigma–Aldrich, HT110116) and observed under a microscope (Leica, DM5500B).

### RT-qPCR

Total RNA was extracted from pgEpiSCs using an RNA prep Pure Cell/Bacteria Kit (TIANGEN, DP430) and then reverse transcribed to cDNA using 5× All-In-One RT Master Mix (Abm, G490). PCR was conducted using 2× RealStar Green Power Mixture (GenStar, A311-05) on a LightCycler 480 II Real Time System (Roche). The data were analyzed using the comparative CT (2^−ΔΔCT^) method. ΔCT was calculated using EF1A as an internal control. Three biological replicates were performed for all experiments. The primers used in quantitative real-time PCR are listed in Supplementary information, Table S6 (Key Resources Table).

### Western blot analysis

Total proteins were extracted from cells by cell lysis buffer (Beyotime Biotechnology, P0013), and nuclear and cytoplasmic proteins were extracted by nuclear and cytoplasmic protein extraction kits (Beyotime Biotechnology, P0027) supplemented with protease and phosphatase inhibitor cocktail (Beyotime Biotechnology, P1050). The concentrations of extracted proteins were measured using the Bradford protein assay kit (Bio-red, 5000201). Equal amounts of protein (15 μg) were separated by SDS–PAGE, and proteins were transferred from the gel to Immobilon-P transfer membranes (Merck Millipore; pore size: 0.45 μm; IPVH00010). The blots were blocked in 5% nonfat powdered milk (Sangon Biotech, A600669-0250) in TBST (20 mM Tris, pH 7.5; 150 mM NaCl; 0.1% Tween 20) at room temperature for 1 h and then incubated with primary antibodies diluted in 5% nonfat powdered milk in TBST overnight at 4 °C. The next day, the blots were rinsed three times for 5 min with TBST, followed by incubation in HRP-conjugated secondary antibodies diluted in 5% nonfat powdered milk in TBST for 1 h at room temperature, and finally rinsed three times for 5 min each with TBST. The blots were exposed to SuperSignal^®^ West Dura Extended Duration Substrate (Thermo Fisher Scientific, 34075), and the band intensities of the target proteins were analyzed by CLINX chemiluminescence software. The specific experimental method and reagent formula came from the general protocol for western blotting (Bio-Rad, Bulletin 6376).

### In situ Hi-C

We separately constructed four Hi-C libraries (as technical replicates) for each of four pgEpiSCs (biological replicates) and eight Hi-C libraries (as technical replicates) for each of two pEFs (biological replicates) according to a previously published in situ Hi-C method^87^ with minor modifications. Briefly, cells (5 × 10^6^) were cross-linked with a final concentration of 2% formaldehyde for 5 min at room temperature followed by quenching with glycine at a final concentration of 0.25 M/L. Mixtures were next centrifuged at 1500× *g* for 10 min at room temperature, and supernatants were combined with lysis buffer and incubated for 15 min on ice. The mixture was then centrifuged at 5000× *g* for 10 min at room temperature. The sediment was washed with 100 μL 1× NEBuffer 2. The mixture was combined with SDS at a final concentration of 0.1% and incubated for 10 min at 65 °C. Then, Triton X-100 was added at a final concentration of 1% and incubated for 15 min at 37 °C. Nuclei of cells were permeabilized, and DNA was digested with 200 units of *Dpn*II (a 4-cutter restriction enzyme) for 1 h at 37 °C. The restriction fragment overhangs were filled and labeled by biotinylated nucleotides and then ligated in a small volume. After crosslink reversal, DNA was purified and sonicated to fragments of approximately 300–500 bp using a Covaris S220 sonicator, at which point ligated fragments were pulled down with Dynabeads™ M-280 Streptavidin (Invitrogen, 11206D), end-repaired and A-tailed. Adaptors were next ligated, and DNA fragments were PCR amplified using a KAPA Hyper Prep Kit (Roche, KK8504) for 8–10 cycles. These fragments were then double-sided size selection using AMPure XP Beads (Beckman, A63882) to isolate fragments between 300 and 800 bp, which were prepped for sequencing on the DNBSEQ platform (BGI) to provide 100 bp paired-end reads (Supplementary information, Data S1-Ia).

### rRNA-depleted RNA-seq

We collected and purified pgEpiSCs derived from four donors (as biological replicates) and pEFs derived from the same back skin area of two donors (as biological replicates) with 1 × 10^6^ cells per replicate. Total RNA from six samples (four pgEpiSCs and two pEFs) was separately extracted using the RNeasy Mini Kit (Qiagen, 74106). We used a rRNA depletion protocol (Globin-Zero Gold rRNA Removal Kit, Illumina, GZG1224) coupled with the NEBNext^®^ Ultra™ Directional RNA Library Prep Kit for Illumina^®^ (NEB, E7420S) to construct the strand-specific RNA-seq library for each sample. All libraries were quantified using the Qubit dsDNA High Sensitivity Assay Kit (Invitrogen, Life Technologies, Q32851) and sequenced on a HiSeq 4000 platform (Illumina) (Supplementary information, Data S1-Ib).

### ATAC-seq

We constructed ATAC-seq libraries for pgEpiSCs and pEFs as previously described. This method can acquire high-quality data from a small number of input cells.^88^ Briefly, cells were lysed with 6 μL lysis buffer for 10 min on ice. Then, we added 5 μL ddH_2_O, 4 μL 5× TTBL, and 5 μL TTE mix V5 (TD502, Vazyme) to the tube, and the mixture was incubated at 37 °C for 30 min. Then, 5 μL of 5× TS buffer (TD502, Vazyme) was added and incubated at room temperature for 5 min to terminate the reaction. Next, we added 40 ng carrier RNA (59824, QIAGEN), 73 μL TE (Tris-EDTA), and 100 μL phenol–chloroform to the reaction product. After vortexing and incubating for 3 min at room temperature, the product was transferred to a phase-lock tube (WM5-2302820, TIANGEN) and centrifuged at 12,000 rpm for 5 min. The supernatant was then transferred to a new 1.5 mL tube, and 650 μL ethanol, 24 μL NaOAc (3 M), and 2 μL glycogen were added for DNA precipitation at −20 °C overnight. The DNA pellet was resuspended in 29 μL ddH_2_O, and 10 μL 5× TAB, 1 μL TAE (TD502, Vazyme), 5 μL N5XX primer, and 5 μL N7XX primer (TD202, Vazyme) were added for PCR. DNA was amplified using the following cycling protocol: 72 °C for 3 min; 98 °C for 30 s; 12 cycles of 98 °C for 15 s, 60 °C for 30 s and 72 °C for 3 min; and 72 °C for 5 min. After amplification, libraries were subjected to size selection with 0.5–1.5× AMPure beads (A63882, Beckman) and sequenced as 150 bp paired-end reads on an Illumina NovaSeq 6000 platform.

### ChIP-seq

We performed ChIP-seq of H3K27ac (a canonical histone marker of enhancers) for two biological replicates of pgEpiSCs and pEFs with 1 × 10^7^ cells per sample. The cells were cross-linked with a final concentration of 1% formaldehyde for 10 min at room temperature, followed by quenching with glycine. Cells were lysed with lysis buffer supplemented with protease inhibitor cocktail and 1 mM PMSF (1× final each) and then sonicated to fragments of approximately 200–500 bp using a Bioruptor. Twenty microliters of chromatin was saved at −20 °C as input DNA, and 100 μL of chromatin was used for immunoprecipitation with 5 μg of H3K27ac antibodies (Abcam, ab4729) at 4 °C overnight. Then, 30 μL of protein beads was added, and the samples were further incubated for 3 h. The beads were next washed once with 20 mM Tris/HCl, pH 8.1, 50 mM NaCl, 2 mM EDTA, 1% Triton X-100, and 0.1% SDS; twice with 10 mM Tris/HCl, pH 8.1, 250 mM LiCl, 1 mM EDTA, 1% NP-40, and 1% deoxycholic acid; and twice with 1× TE buffer (10 mM Tris-Cl at pH 7.5, 1 mM EDTA). Bound material was then eluted from the beads in 300 μL of elution buffer (100 mM NaHCO_3_, 1% SDS), treated with RNase A (final concentration 8 μg/mL) for 6 h at 65 °C and then with proteinase K (final concentration 345 μg/mL) overnight at 45 °C. The immunoprecipitated DNA was used to construct sequencing libraries following the protocol provided by the NEXTflex™ ChIP-Seq Kit (Bioo Scientific, NOVA-5143-02). All libraries were sequenced on a HiSeq XTen (Illumina) platform (Supplementary information, Data S1-Ib).

### Vector construction

To test whether pgEpiSCs can tolerate successive gene editing, we conducted three gene editing experiments with different gene editing technologies in pgEpiSCs: (1) GFP-NLS cassette stable transfection using PiggyBac (PB) transposase tools; (2) NANOG-tdTomato reporter knock-in via CRISPR/Cas9 systems; and (3) *TYR* gene point mutation with Cytidine Base Editors (CBEs).

First, to generate GFP-positive cells, we constructed a PB-CMV-EF1A-GFP-NLS plasmid based on the PB-CAG-MCS vector (from Prof. Sen Wu), replaced the chicken β-actin promoter with the *Homo sapiens* elongation Factor 1 alpha (EF1A) promoter, and inserted a GFP-NLS cassette at the end of the EF1A promoter. Second, to obtain NANOG-tdTomato knock-in cell lines, we constructed a *NANOG* DNA donor vector with four fragments, Backbone, Left Homology Arm-3× Flag, 3× Flag-P2A-tdTomato-Loxp-Puro-Loxp and Right Homology Arm, using NEBuilder^®^ HiFi DNA Assembly Master Mix (NEB, E2621X) as previously described.^89^ The *NANOG* sgRNA targeted the sequence before the stop codon site to knock in the donor fragment as a reporter. The annealed sgRNA sequence was cloned into the *Bsa*I-digested pGL3-U6-sgRNA-PGK-puromycin vector (Addgene, 51133). Finally, we used the AncBE4max plasmid to knock out the *TYR* gene. AncBE4max and the pGL3-U6-sgRNA-EGFP vector were obtained from Xingxu Huang’s Laboratory at ShanghaiTech University. SgRNA was synthesized by BGI with the ACCG sequence at the 5’ end of the forward primer and the AAAC sequence at the 5’ end of the reverse primer. Then, sgRNA was annealed and cloned into the pGL3-U6-sgRNA-EGFP vector. The details of sgRNA sequences are provided in Supplementary information, Table S6 (Key Resources Table).

### Cell electroporation

Before electroporation, the pgEpiSCs were dissociated using Accutase Cell Dissociation Reagent (Gibco, A11105-01). For each electroporation, 5 × 10^5^ cells were transfected at 220 V, 5 ms, 2 pulses using a BTX ECM 2001 instrument (Harvard Bioscience, Holliston, MA, USA). For GFP-NLS cassette stable transfection, electroporation was performed with 1 μg PBase helper plasmids and 3 μg PB-CMV-EF1A-GFP-NLS donor plasmids (mass ratio 1:3). For NANOG-tdTomato reporter knock-in, electroporation was performed with 1 μg pST1374-NLS-flag-linker-Cas9 plasmids (Addgene, 44758), 1 μg *NANOG* sgRNA plasmid and 1 μg *NANOG* HMEJ donor plasmids (1 μg of each vector), and for *TYR* gene point mutation, electroporation was performed with 2 μg of ancBE4max vector and 2 μg of pGL3-U6-TYR sgRNA-GFP vectors (mass ratio 1:1). Electrotransfection buffer was provided by the Sen Wu lab at the State Key Laboratory of Agro-biotechnology, China Agricultural University. The primers were designed online using NCBI primer BLAST and synthesized by BGI.

GFP-positive cells were sorted using FACS (MoFlo XDP, Beckman) and detected using the 488 nm (710/50 bandpass filter) channel. To obtain NANOG-tdTomato-positive cells, transfected cells were selected with puromycin (0.3 μg/mL) and blasticidin (4 μg/mL), and GFP-positive colonies were picked and expanded. To identify the base-edited cells, DNA was extracted using cell lysis buffer (Invitrogen, AM8723) and used as a PCR template. The PCR products were sequenced to confirm the point mutation.

### Chimera assay

Pig SCNT embryos were produced using Yorkshire embryonic fibroblasts as donor cells and used for chimerism assays. We cultured pgEpiSCs for 36–48 h after subculture and then injected the cells into the cavity of early SCNT blastocysts at E5.

For embryonic injection, pgEpiSCs were first dissociated using Accutase cell dissociation reagent (Gibco, A11105-01). Afterwards, the cells were centrifuged at 800–1000 rpm at room temperature for 5 min. The supernatant was removed, and the cells were resuspended to an appropriate density (5 × 10^5^ cells/mL) in fresh culture medium (10 μM Y27632 was added directly to the suspension when indicated). The suspension was kept on ice before injection. A total of 15–20 cells were injected into the blastocoel near the ICM through a XYRCOS laser system (Hamilton Thorne, Inc., Beverly, MA, USA), and following cell injection, blastocysts were cultured in 3i/LAF and PZM-3 mix medium (1:1) for 36 h for immunostaining analysis to check if the pgEpiSCs incorporated the ICMs. For chimeric embryo transfer, the blastocysts were cultured in 3i/LAF and PZM-3 mix medium (1:1) for 12 h before transplantation.

### Generation of pgEpiSCs cloned embryos

Ovaries were collected from slaughterhouses around Beijing. Oocytes with three or four layers of cumulus cells were selected and cultured in IVM solution for 44 h at 38.5 °C, 100% humidity, and 5% CO_2_. IVM mother solution M199 (Sigma, M2154) containing 0.1% L-cysteine (Sigma, C7352-25G), 5% FBS (Gibco, 10099141), 0.1% EGF (Sigma, E9644), 1% penicillin–streptomycin (Gibco, 15140122), and 10% pig follicular fluid (follicular fluid was collected during oocyte acquisition, centrifuged and filtered, and stored at –80 °C). After preparation, the IVM mother solution was filtered with a 0.22-μm filter and stored at 4 °C for later use. Before use, 1% GlutaMAX (Gibco, 35050061), 10 IU/mL PMSG, and 10 IU/mL hCG were added.

Pig pgEpiSCs were differentiated in basal medium containing 10 ng/mL BMP4, 5 μM SB431542, and 10 ng/mL FGF2 for more than 1 week and then used as donor cells for nuclear transfer. Matured oocytes in metaphase II were enucleated by micromanipulation in TCM199/HEPES (Theriogenology Sigma, M-2520) containing 7.5 μg/mL cytochalasin B. A morphologically qualified donor cell was injected into the perivitelline space and fused with the recipient cytoplasm using a BLS Electro-cell Manipulator in fusion medium (0.3 M/L mannitol, 1.0 mM/L CaCl_2_, 0.1 mM/L MgCl_2_, and 0.5 mM/L HEPES). Oocytes were then incubated for 15 min in PZM-3, and the fusion percentage was evaluated under a stereomicroscope. Fifty to sixty fused embryos were placed into four-well dishes containing 500 μL PZM-3 per well and cultured in PZM-3 at 38.5 °C in 5% CO_2_, 5% O_2_, and 90% N_2_ with maximum humidity. After 24 h, 150–250 reconstructed embryos were transferred surgically into the uterus of a surrogate mother. The pregnancy status of the surrogates was diagnosed by ultrasonography at 25–30 days. All cloned piglets were delivered by natural birth on days 114–120 of gestation.

### Statistical analysis

The Wilcoxon rank-sum test was applied to analyze the data presented in Fig. 5d–f and Supplementary information, Fig. S6a, b, f–h. Two-way ANOVA multiple comparison test was used to analyze data of RT-qPCR in Fig. 3b, d, f and Supplementary information, Fig. S3e, g, and used to analyze data presented in Figs. 2d and 3h, i. Welch’s unpaired *t*-test was utilized to compare the population doubling time and single-cell cloning efficiency in Fig. 2e, f. Spearman’s correlation was performed to calculate the correlation coefficient (*r*) and statistical significance with the function “cor” in R (Fig. 4d and Supplementary information, Fig. S5g). DEGs for scRNA-seq data were analyzed using the Wilcoxon rank-sum test, and the *P* values were adjusted using the Benjamini–Hochberg method. More materials and methods are available in Supplementary information, Data S3.

## Supplementary information

Supplementary information, Figure S1

Supplementary information, Figure S2

Supplementary information, Figure S3

Supplementary information, Figure S4

Supplementary information, Figure S5

Supplementary information, Figure S6

Supplementary information, Figure S7

Supplementary information, Figure S8

Supplementary information, Figure S9

Supplementary information, Figure S10

Supplementary information, Data S1

Supplementary information, Data S2

Supplementary information, Data S3

Supplementary information, Table S1

Supplementary information, Table S2

Supplementary information, Table S3

Supplementary information, Table S4

Supplementary information, Table S5

Supplementary information, Table S6

## Data Availability

All datasets generated during this study are available at Genome Sequence Archive (GSA) (https://ngdc.cncb.ac.cn/gsa/) of China National Center for Bioinformation-National Genomics Data Center (CNCB-NGDC) with accession code: CRA003960.

## Material availability

Further information and requests for resources and reagents should be directed to and will be fulfilled by the Lead Contact, Jianyong Han (hanjy@cau.edu.cn). All stable pig epiblast stem cell lines generated in this study are available from the Lead Contact with a completed Materials Transfer Agreement.

## References

[1] Chazaud C, Yamanaka Y (2016). Lineage specification in the mouse preimplantation embryo. Development.

[2] Rossant J, Tam PPL (2018). Exploring early human embryo development. Science.

[3] Boroviak T, Loos R, Bertone P, Smith A, Nichols J (2014). The ability of inner-cell-mass cells to self-renew as embryonic stem cells is acquired following epiblast specification. Nat. Cell Biol..

[4] Martin GR (1981). Isolation of a pluripotent cell line from early mouse embryos cultured in medium conditioned by teratocarcinoma stem cells. Proc. Natl. Acad. Sci. USA.

[5] Evans MJ, Kaufman MH (1981). Establishment in culture of pluripotential cells from mouse embryos. Nature.

[6] Ying QL (2008). The ground state of embryonic stem cell self-renewal. Nature.

[7] Brons IG (2007). Derivation of pluripotent epiblast stem cells from mammalian embryos. Nature.

[8] Tesar PJ (2007). New cell lines from mouse epiblast share defining features with human embryonic stem cells. Nature.

[9] Bao S (2009). Epigenetic reversion of post-implantation epiblast to pluripotent embryonic stem cells. Nature.

[10] Kinoshita M (2021). Capture of mouse and human stem cells with features of formative pluripotency. Cell Stem Cell.

[11] Yu L (2021). Derivation of intermediate pluripotent stem cells amenable to primordial germ cell specification. Cell Stem Cell.

[12] Wang X (2021). Formative pluripotent stem cells show features of epiblast cells poised for gastrulation. Cell Res..

[13] Thomson JA (1998). Embryonic stem cell lines derived from human blastocysts. Science.

[14] Gafni O (2013). Derivation of novel human ground state naïve pluripotent stem cells. Nature.

[15] Theunissen TW (2014). Systematic identification of culture conditions for induction and maintenance of naïve human pluripotency. Cell Stem Cell.

[16] Kobayashi T (2017). Principles of early human development and germ cell program from conserved model systems. Nature.

[17] Niu D (2017). Inactivation of porcine endogenous retrovirus in pigs using CRISPR-Cas9. Science.

[18] Yue Y (2021). Extensive germline genome engineering in pigs. Nat. Biomed. Eng..

[19] Yan S (2018). A Huntingtin Knockin pig model recapitulates features of selective neurodegeneration in Huntington’s disease. Cell.

[20] Navarro M, Soto DA, Pinzon CA, Wu J, Ross PJ (2020). Livestock pluripotency is finally captured in vitro. Reprod. Fertil. Dev..

[21] Zhao JG, Lai LX, Ji WZ, Zhou Q (2019). Genome editing in large animals: current status and future prospects. Natl. Sci. Rev..

[22] Notarianni E, Laurie S, Moor RM, Evans MJ (1990). Maintenance and differentiation in culture of pluripotential embryonic cell lines from pig blastocysts. J. Reprod. Fertil. Suppl..

[23] Vassiliev I (2010). In vitro and in vivo characterization of putative porcine embryonic stem cells. Cell Reprogram..

[24] Gao X (2019). Establishment of porcine and human expanded potential stem cells. Nat. Cell Biol..

[25] Choi KH (2019). Chemically defined media can maintain pig pluripotency network in vitro. Stem Cell Rep..

[26] Yuan Y (2019). A six-inhibitor culture medium for improving naïve-type pluripotency of porcine pluripotent stem cells. Cell Death Discov..

[27] Hou DR (2016). Derivation of porcine embryonic stem-like cells from in vitro-produced blastocyst-stage embryos. Sci. Rep..

[28] Alberio R, Croxall N, Allegrucci C (2010). Pig epiblast stem cells depend on activin/nodal signaling for pluripotency and self-renewal. Stem Cells Dev..

[29] Ramos-Ibeas P (2019). Pluripotency and X chromosome dynamics revealed in pig pre-gastrulating embryos by single cell analysis. Nat. Commun..

[30] Liu T (2021). Cross-species single-cell transcriptomic analysis reveals pre-gastrulation developmental differences among pigs, monkeys, and humans. Cell Discov..

[31] Tang F (2010). Tracing the derivation of embryonic stem cells from the inner cell mass by single-cell RNA-Seq analysis. Cell Stem Cell.

[32] Yan L (2013). Single-cell RNA-Seq profiling of human preimplantation embryos and embryonic stem cells. Nat. Struct. Mol. Biol..

[33] Nakamura T (2016). A developmental coordinate of pluripotency among mice, monkeys and humans. Nature.

[34] Petropoulos S (2016). Single-cell RNA-Seq reveals lineage and X chromosome dynamics in human preimplantation embryos. Cell.

[35] Zhou F (2019). Reconstituting the transcriptome and DNA methylome landscapes of human implantation. Nature.

[36] Pijuan-Sala B (2019). A single-cell molecular map of mouse gastrulation and early organogenesis. Nature.

[37] Cao S (2014). Specific gene-regulation networks during the pre-implantation development of the pig embryo as revealed by deep sequencing. BMC Genomics.

[38] Wei Q (2018). Lineage specification revealed by single-cell gene expression analysis in porcine preimplantation embryos. Biol. Reprod..

[39] Gao S (2018). Tracing the temporal-spatial transcriptome landscapes of the human fetal digestive tract using single-cell RNA-sequencing. Nat. Cell Biol..

[40] Wang M (2018). Single-cell RNA sequencing analysis reveals sequential cell fate transition during human spermatogenesis. Cell Stem Cell.

[41] Stuart T (2019). Comprehensive integration of single-cell data. Cell.

[42] Edgar R (2013). LifeMap Discovery™: the embryonic development, stem cells, and regenerative medicine research portal. PLoS One.

[43] Wu J (2016). The landscape of accessible chromatin in mammalian preimplantation embryos. Nature.

[44] Plusa B, Piliszek A, Frankenberg S, Artus J, Hadjantonakis AK (2008). Distinct sequential cell behaviours direct primitive endoderm formation in the mouse blastocyst. Development.

[45] Saiz N, Williams KM, Seshan VE, Hadjantonakis AK (2016). Asynchronous fate decisions by single cells collectively ensure consistent lineage composition in the mouse blastocyst. Nat. Commun..

[46] Oestrup O (2009). From zygote to implantation: morphological and molecular dynamics during embryo development in the pig. Reprod. Domest. Anim..

[47] Zhou Y (2019). Metascape provides a biologist-oriented resource for the analysis of systems-level datasets. Nat. Commun..

[48] Greber B (2010). Conserved and divergent roles of FGF signaling in mouse epiblast stem cells and human embryonic stem cells. Cell Stem Cell.

[49] Kim H (2013). Modulation of beta-catenin function maintains mouse epiblast stem cell and human embryonic stem cell self-renewal. Nat. Commun..

[50] van Oosten AL, Costa Y, Smith A, Silva JC (2012). JAK/STAT3 signalling is sufficient and dominant over antagonistic cues for the establishment of naïve pluripotency. Nat. Commun..

[51] Takashima Y (2014). Resetting transcription factor control circuitry toward ground-state pluripotency in human. Cell.

[52] Yang J (2017). Establishment of mouse expanded potential stem cells. Nature.

[53] Yang Y (2017). Derivation of pluripotent stem cells with in vivo embryonic and extraembryonic potency. Cell.

[54] Li X (2011). Calcineurin-NFAT signaling critically regulates early lineage specification in mouse embryonic stem cells and embryos. Cell Stem Cell.

[55] Probst S (2021). Spatiotemporal sequence of mesoderm and endoderm lineage segregation during mouse gastrulation. Development.

[56] Valdez Magana G, Rodriguez A, Zhang H, Webb R, Alberio R (2014). Paracrine effects of embryo-derived FGF4 and BMP4 during pig trophoblast elongation. Dev. Biol..

[57] Xu J (2019). Generation of pig induced pluripotent stem cells using an extended pluripotent stem cell culture system. Stem Cell Res. Ther..

[58] Shi B (2020). IRF-1 expressed in the inner cell mass of the porcine early blastocyst enhances the pluripotency of induced pluripotent stem cells. Stem Cell Res. Ther..

[59] Secher JO (2017). Systematic in vitro and in vivo characterization of Leukemia-inhibiting factor- and fibroblast growth factor-derived porcine induced pluripotent stem cells. Mol. Reprod. Dev..

[60] Guo G (2017). Epigenetic resetting of human pluripotency. Development.

[61] Ji X (2016). 3D chromosome regulatory landscape of human pluripotent cells. Cell Stem Cell.

[62] Stadhouders R, Filion GJ, Graf T (2019). Transcription factors and 3D genome conformation in cell-fate decisions. Nature.

[63] Zheng H, Xie W (2019). The role of 3D genome organization in development and cell differentiation. Nat. Rev. Mol. Cell Biol..

[64] Lindsly S (2021). 4DNvestigator: time series Hi-C and RNA-seq data analysis toolbox. Nucleus.

[65] Tan L, Xing D, Chang CH, Li H, Xie XS (2018). Three-dimensional genome structures of single diploid human cells. Science.

[66] Bonev B (2017). Multiscale 3D genome rewiring during mouse neural development. Cell.

[67] Dixon JR (2015). Chromatin architecture reorganization during stem cell differentiation. Nature.

[68] Zhou Y (2014). Chitinase 3-like 1 suppresses injury and promotes fibroproliferative responses in Mammalian lung fibrosis. Sci. Transl. Med..

[69] Khan A (2018). JASPAR 2018: update of the open-access database of transcription factor binding profiles and its web framework. Nucleic Acids Res..

[70] Dixon JR (2012). Topological domains in mammalian genomes identified by analysis of chromatin interactions. Nature.

[71] Nir G (2018). Walking along chromosomes with super-resolution imaging, contact maps, and integrative modeling. PLoS Genet..

[72] Lyu X, Rowley MJ, Corces VG (2018). Architectural proteins and pluripotency factors cooperate to orchestrate the transcriptional response of hESCs to temperature stress. Mol. Cell.

[73] Di Giammartino DC (2019). KLF4 is involved in the organization and regulation of pluripotency-associated three-dimensional enhancer networks. Nat. Cell Biol..

[74] Cao Q (2017). Reconstruction of enhancer-target networks in 935 samples of human primary cells, tissues and cell lines. Nat. Genet..

[75] Fulco CP (2019). Activity-by-contact model of enhancer-promoter regulation from thousands of CRISPR perturbations. Nat. Genet..

[76] Whalen S, Truty RM, Pollard KS (2016). Enhancer-promoter interactions are encoded by complex genomic signatures on looping chromatin. Nat. Genet..

[77] Schoenfelder S, Fraser P (2019). Long-range enhancer-promoter contacts in gene expression control. Nat. Rev. Genet..

[78] Schoenfelder S (2015). The pluripotent regulatory circuitry connecting promoters to their long-range interacting elements. Genome Res..

[79] Javierre BM (2016). Lineage-specific genome architecture links enhancers and non-coding disease variants to target gene promoters. Cell.

[80] Kuleshov MV (2016). Enrichr: a comprehensive gene set enrichment analysis web server 2016 update. Nucleic Acids Res..

[81] MacArthur BD, Lemischka IR (2013). Statistical mechanics of pluripotency. Cell.

[82] Komor AC, Kim YB, Packer MS, Zuris JA, Liu DR (2016). Programmable editing of a target base in genomic DNA without double-stranded DNA cleavage. Nature.

[83] Xie J (2019). Efficient base editing for multiple genes and loci in pigs using base editors. Nat. Commun..

[84] Li Z (2018). Efficient RNA-guided base editing for disease modeling in pigs. Cell Discov..

[85] Xue B (2016). Porcine pluripotent stem cells derived from IVF embryos contribute to chimeric development in vivo. PLoS One.

[86] Fan N (2013). Piglets cloned from induced pluripotent stem cells. Cell Res..

[87] Rao SS (2014). A 3D map of the human genome at kilobase resolution reveals principles of chromatin looping. Cell.

[88] Wu J (2018). Chromatin analysis in human early development reveals epigenetic transition during ZGA. Nature.

[89] Xu J (2020). A cytokine screen using CRISPR-Cas9 knock-in reporter pig iPS cells reveals that Activin A regulates NANOG. Stem Cell Res. Ther..

