# Supplementary material for: Generation and characterization of stable pig pregastrulation epiblast stem cell lines
Source: Cell Res. 2021 Nov 30;32(4):383–400. doi: 10.1038/s41422-021-00592-9 (PMC8976023; doi:10.1038/s41422-021-00592-9)
Supplement: Supplementary file 1 — Supplementary information, Figure S1 [file 41422_2021_592_MOESM1_ESM.pdf]

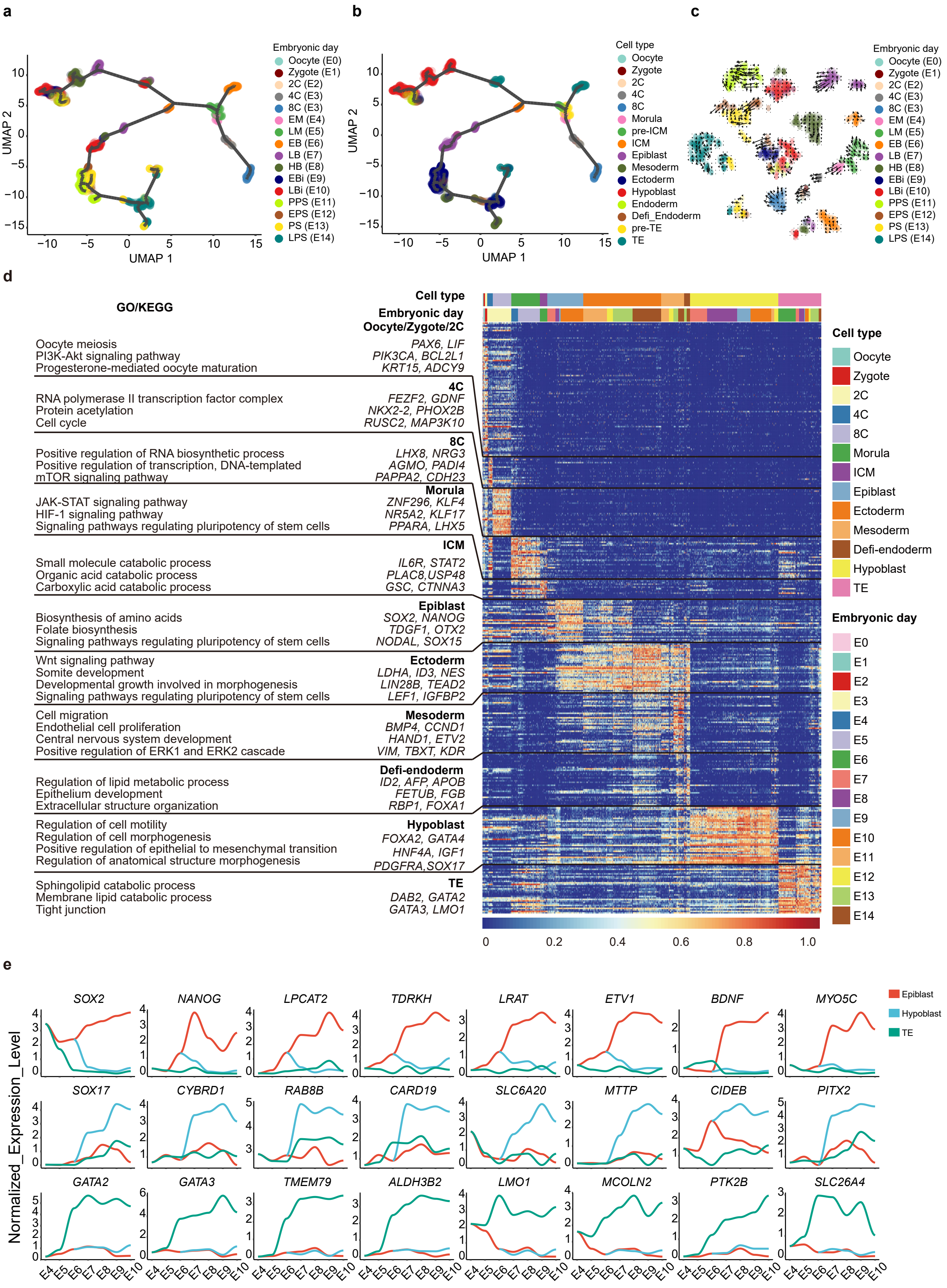
a
b
c
UMAP 1
UMAP 2
Embryonic day
Oocyte (E0)
Zygote (E1)
2C (E2)
4C (E3)
8C (E3)
EM (E4)
LM (E5)
EB (E6)
LB (E7)
HB (E8)
EBi (E9)
LBi (E10)
PPS (E11)
EPS (E12)
PS (E13)
LPS (E14)
Cell type
Oocyte
Zygote
2C
4C
8C
Morula
pre-ICM
ICM
Epiblast
Mesoderm
Ectoderm
Hypoblast
Endoderm
Defi_Endoderm
pre-TE
TE
d
GO/KEGG
Cell type
Embryonic day
Oocyte/Zygote/2C
Oocyte meiosis
PI3K-Akt signaling pathway
Progesterone-mediated oocyte maturation
PAX6, LIF
PIK3CA, BCL2L1
KRT15, ADCY9
4C
RNA polymerase II transcription factor complex
Protein acetylation
Cell cycle
FEZF2, GDNF
NKX2-2, PHOX2B
RUSC2, MAP3K10
8C
Positive regulation of RNA biosynthetic process
Positive regulation of transcription, DNA-templated
mTOR signaling pathway
LHX8, NRG3
AGMO, PADI4
PAPPA2, CDH23
Morula
JAK-STAT signaling pathway
HIF-1 signaling pathway
Signaling pathways regulating pluripotency of stem cells
ZNF296, KLF4
NR5A2, KLF17
PPARA, LHX5
ICM
Small molecule catabolic process
Organic acid catabolic process
Carboxylic acid catabolic process
IL6R, STAT2
PLAC8,USP48
GSC, CTNNA3
Epiblast
Biosynthesis of amino acids
Folate biosynthesis
Signaling pathways regulating pluripotency of stem cells
SOX2, NANOG
TDGF1, OTX2
NODAL, SOX15
Ectoderm
Wnt signaling pathway
Somite development
Developmental growth involved in morphogenesis
Signaling pathways regulating pluripotency of stem cells
LDHA, ID3, NES
LIN28B, TEAD2
LEF1, IGFBP2
Mesoderm
Cell migration
Endothelial cell proliferation
Central nervous system development
Positive regulation of ERK1 and ERK2 cascade
BMP4, CCND1
HAND1, ETV2
VIM, TBXT, KDR
Defi-endoderm
Regulation of lipid metabolic process
Epithelium development
Extracellular structure organization
ID2, AFP, APOB
FETUB, FGB
RBP1, FOXA1
Hypoblast
Regulation of cell motility
Regulation of cell morphogenesis
Positive regulation of epithelial to mesenchymal transition
Regulation of anatomical structure morphogenesis
FOXA2, GATA4
HNF4A, IGF1
PDGFRA,SOX17
TE
Sphingolipid catabolic process
Membrane lipid catabolic process
Tight junction
DAB2, GATA2
GATA3, LMO1
0 0.2 0.4 0.6 0.8 1.0
Cell type
Oocyte
Zygote
2C
4C
8C
Morula
ICM
Epiblast
Ectoderm
Mesoderm
Defi-endoderm
Hypoblast
TE
Embryonic day
E0
E1
E2
E3
E4
E5
E6
E7
E8
E9
E10
E11
E12
E13
E14
e
Normalized_Expression_Level
SOX2
NANOG
LPCAT2
TDRKH
LRAT
ETV1
BDNF
MYO5C
SOX17
CYBRD1
RAB8B
CARD19
SLC6A20
MTTP
CIDEB
PITX2
GATA2
GATA3
TMEM79
ALDH3B2
LMO1
MCOLN2
PTK2B
SLC26A4
E4 E5 E6 E7 E8 E9 E10
Epiblast
Hypoblast
TE

Fig. S1: Single Cell RNA Sequencing of Pig Pre-implantation Embryos, Related to Fig. 1

a, b Developmental trajectories deduced by pseudotime analysis using monocle 3 and visualized according to embryonic days (**a**) and cell types or lineages (**b**). **c** RNA velocities are visualized on t-SNE plots for the cells at each embryonic stage from E0 to E14. **d** Heat map for the expression of lineage specific genes. The genes were ordered by unsupervised hierarchical clustering (UHC) in each cell type. The representative functional categories that were enriched by relevant genes are shown. **e** Time-course expression profile of canonical and novel lineage markers for epiblast, hypoblast and trophectoderm.
