# Supplementary material for: Generation and characterization of stable pig pregastrulation epiblast stem cell lines
Source: Cell Res. 2021 Nov 30;32(4):383–400. doi: 10.1038/s41422-021-00592-9 (PMC8976023; doi:10.1038/s41422-021-00592-9)
Supplement: Supplementary file 2 — Supplementary information, Figure S2 [file 41422_2021_592_MOESM2_ESM.pdf]

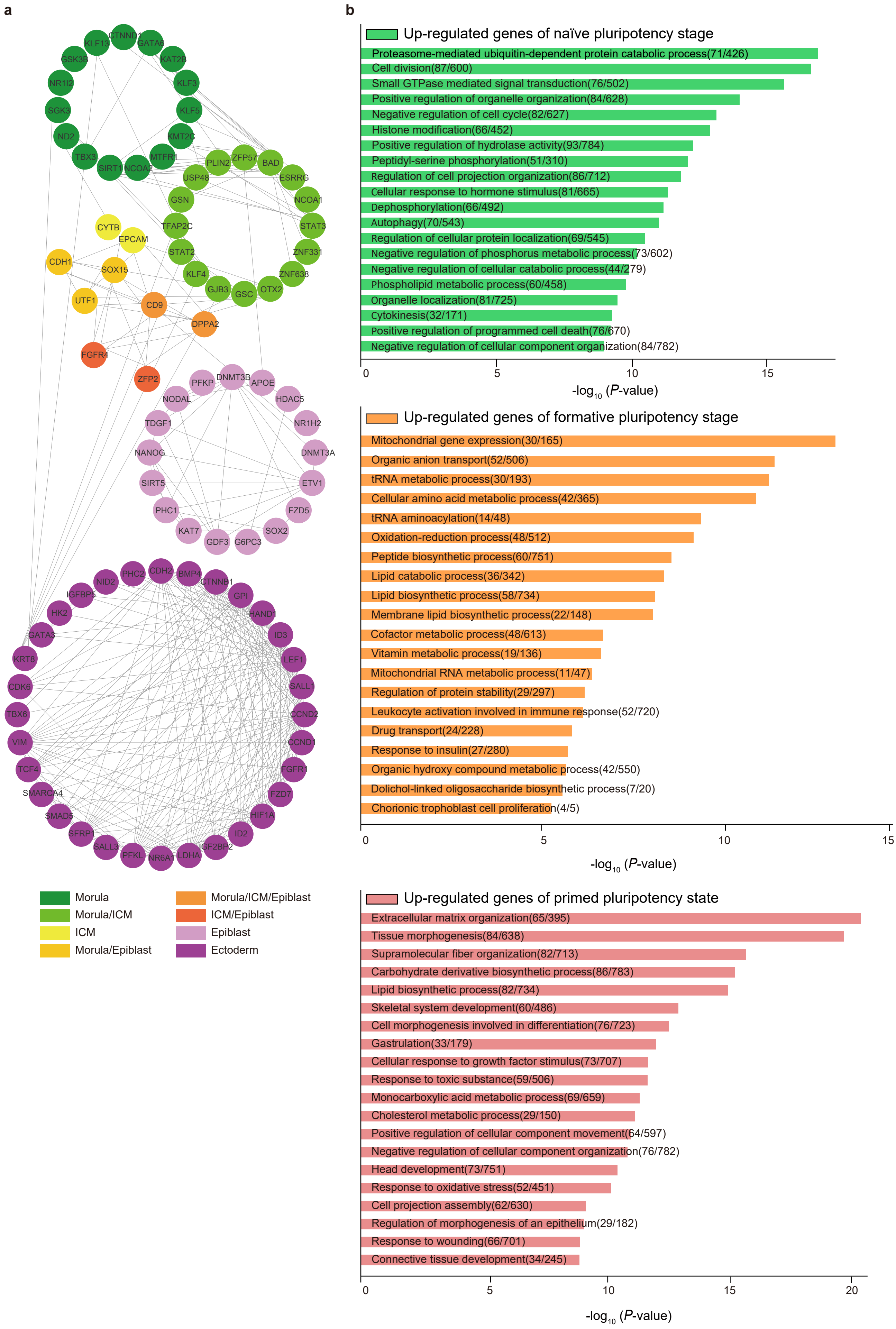
a
CTNND1
KLF13
GATA6
GSK3B
KAT2B
NR1I2
KLF3
SGK3
KLF5
ND2
KMT2C
TBX3
MTFR1
SIRT1
NCOA2
PLIN2
ZFP57
BAD
USP48
ESRRG
GSN
NCOA1
TFAP2C
STAT3
CYTB
EPCAM
ZNF331
CDH1
STAT2
SOX15
KLF4
ZNF638
GJB3
GSC
OTX2
UTF1
CD9
DPPA2
FGFR4
ZFP2
PFKP
DNMT3B
APOE
NODAL
HDAC5
TDGF1
NR1H2
NANOG
DNMT3A
SIRT5
ETV1
PHC1
FZD5
KAT7
SOX2
GDF3
G6PC3
NID2
PHC2
CDH2
BMP4
CTNNB1
IGFBP5
GPI
HK2
HAND1
GATA3
ID3
KRT8
LEF1
CDK6
SALL1
TBX6
CCND2
VIM
CCND1
TCF4
FGFR1
SMARCA4
FZD7
SMAD5
HIF1A
SFRP1
ID2
SALL3
IGF2BP2
PFKL
NR6A1
LDHA
Morula
Morula/ICM
ICM
Morula/Epiblast
Morula/ICM/Epiblast
ICM/Epiblast
Epiblast
Ectoderm
b
Up-regulated genes of naïve pluripotency stage
Proteasome-mediated ubiquitin-dependent protein catabolic process(71/426)
Cell division(87/600)
Small GTPase mediated signal transduction(76/502)
Positive regulation of organelle organization(84/628)
Negative regulation of cell cycle(82/627)
Histone modification(66/452)
Positive regulation of hydrolase activity(93/784)
Peptidyl-serine phosphorylation(51/310)
Regulation of cell projection organization(86/712)
Cellular response to hormone stimulus(81/665)
Dephosphorylation(66/492)
Autophagy(70/543)
Regulation of cellular protein localization(69/545)
Negative regulation of phosphorus metabolic process(73/602)
Negative regulation of cellular catabolic process(44/279)
Phospholipid metabolic process(60/458)
Organelle localization(81/725)
Cytokinesis(32/171)
Positive regulation of programmed cell death(76/670)
Negative regulation of cellular component organization(84/782)
0
5
10
15
$-\log_{10}$ (P-value)
Up-regulated genes of formative pluripotency stage
Mitochondrial gene expression(30/165)
Organic anion transport(52/506)
tRNA metabolic process(30/193)
Cellular amino acid metabolic process(42/365)
tRNA aminoacylation(14/48)
Oxidation-reduction process(48/512)
Peptide biosynthetic process(60/751)
Lipid catabolic process(36/342)
Lipid biosynthetic process(58/734)
Membrane lipid biosynthetic process(22/148)
Cofactor metabolic process(48/613)
Vitamin metabolic process(19/136)
Mitochondrial RNA metabolic process(11/47)
Regulation of protein stability(29/297)
Leukocyte activation involved in immune response(52/720)
Drug transport(24/228)
Response to insulin(27/280)
Organic hydroxy compound metabolic process(42/550)
Dolichol-linked oligosaccharide biosynthetic process(7/20)
Chorionic trophoblast cell proliferation(4/5)
0
5
10
15
$-\log_{10}$ (P-value)
Up-regulated genes of primed pluripotency state
Extracellular matrix organization(65/395)
Tissue morphogenesis(84/638)
Supramolecular fiber organization(82/713)
Carbohydrate derivative biosynthetic process(86/783)
Lipid biosynthetic process(82/734)
Skeletal system development(60/486)
Cell morphogenesis involved in differentiation(76/723)
Gastrulation(33/179)
Cellular response to growth factor stimulus(73/707)
Response to toxic substance(59/506)
Monocarboxylic acid metabolic process(69/659)
Cholesterol metabolic process(29/150)
Positive regulation of cellular component movement(64/597)
Negative regulation of cellular component organization(76/782)
Head development(73/751)
Response to oxidative stress(52/451)
Cell projection assembly(62/630)
Regulation of morphogenesis of an epithelium(29/182)
Response to wounding(66/701)
Connective tissue development(34/245)
0
5
10
15
20
$-\log_{10}$ (P-value)

Fig. S2: Dynamic Changes of Pluripotency in Pig Pre-implantation Embryos, Related to Fig. 1

a Network showing the differentially expressed genes (DEGs) in each cell type compared to other cell types. The different colors indicate different cell types. The circles denote the collections of DEGs. Each cell type is connected with its DEGs by the internal lines of the network. **b** A collection of four naïve pluripotency related clusters with functional enrichment for 1 735 genes from Fig. 1f (green bars), and 1 117 genes from four formative pluripotency related clusters (orange bars), and 1 289 genes from four primed pluripotency related clusters (red bars). The top functional terms of Metascape (https://metascape.org; see Materials and methods) summary gene set in each enriched cluster are shown, with the constraint of showing no more than 20 terms. The number after each term represents the hit genes out of total genes for this term.
