# Supplementary material for: Generation and characterization of stable pig pregastrulation epiblast stem cell lines
Source: Cell Res. 2021 Nov 30;32(4):383–400. doi: 10.1038/s41422-021-00592-9 (PMC8976023; doi:10.1038/s41422-021-00592-9)
Supplement: Supplementary file 3 — Supplementary information, Figure S3 [file 41422_2021_592_MOESM3_ESM.pdf]

a

Signaling pathways	Inhibitors and Cytokines	Culture Conditions								
		LAF	C/LAF	CI/L	CI/LAF	2iGo/L	5i/LA	mEPSCM	LCDM	3i/LAF
WNT/β-catenin	GSK3β i (CHIR99021)		+	+	+	+	+	+	+	+
	WNT i (IWR-1-endo)			+	+					+
	WNT i (XAV939)							+		
	SRC i (WH-4-023)						+	+		+
FGF/ERK	FGF2	+	+		+					+
	PD0325901					+	+	+		
Activin/Nodal	Activin A	+	+		+		+			+
JAK/STAT3	LIF	+	+	+	+	+	+	+	+	+
Others	ROCK i (Y27632)	+	+	+	+	+	+	+	+	+
	pan-PKC i (GO 6983)					+				
	BRAF i (SB590885)						+			
	DIM								+	
	MIH								+	
	JNK i VIII							+		
	P38 MAPK i (SB203580)							+		
Attachment Rate		6/8	8/8	7/8	5/5	8/8	5/6	6/6	8/8	8/8
Outgrowth Rate		5/8	6/8	3/8	5/5	0	0	0	6/8	8/8
Characteristics		AP+; Epithelial morphology; Maximum generation<P10	Partial AP+; Epithelial morphology; No well defined borders; Maximum generation<P5	AP-; Epithelial morphology; Tublin β-III+; Maximum generation<P5	AP+; Heterogeneous expression of POU5F1/GATA6	Unable to subculture	Unable to subculture	Unable to subculture	AP-; Neural differentiation; Maximum generation<P8	AP+; Dome-liked morphology; generation >P240

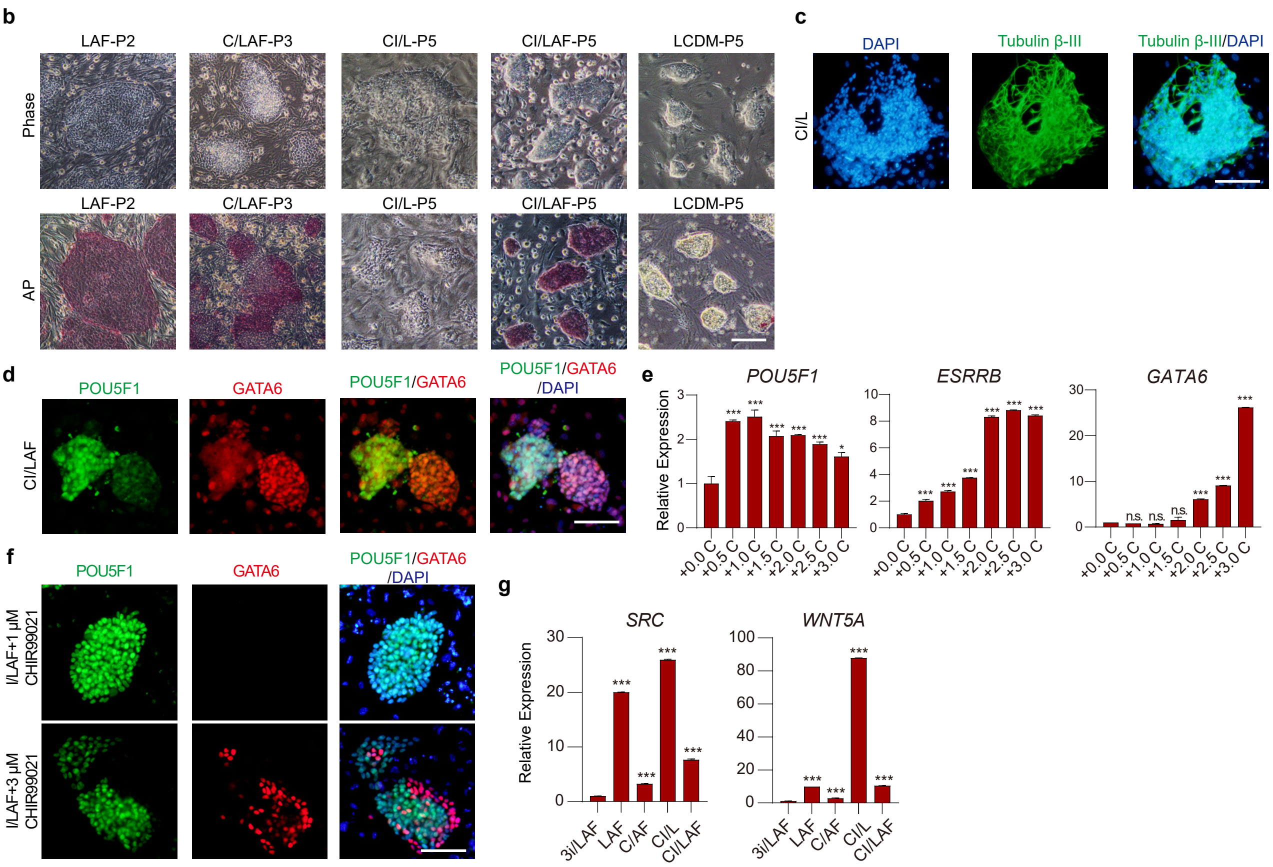

Fig. S3. Establishment of pgEpiSC culture condition, Related to Fig. 2

a Summary of the designed and tested culture conditions. “+” means that the corresponding small molecule inhibitor or cytokine has been added to the culture condition. Attachment rate represents the rate of cell masses visible on feeder cells after epiblast cell seeding for 48 hours. Outgrowth rate represents the rate of growth and expansion of adherent cells. **b** Cell morphology and AP staining of cells in different culture conditions. Scale bar, 200 μm. **c** Immunostaining of neuroectoderm marker Tubulin β-III for the cells under CI/L culture condition. The nucleus is indicated by DAPI. Scale bar, 100 μm. **d** Immunostaining of POU5F1 and GATA6 for the cells under CI/LAF culture condition. The nucleus is indicated by DAPI. Scale bar, 100 μm. **e** Relative expression levels of *POU5F1*, *ESRRB* and *GATA6* in the cells cultured under I/LAF condition treated with different concentrations of C (CHIR99021), the concentration unit, μM. **f** Immunostaining of POU5F1 and GATA6 for the cells under CI/LAF condition treated with different concentrations of CHIR99021. The cell nucleus is indicated by DAPI. Scale bar, 100 μm. **g** Relative expression levels of EMT related genes *SRC* and *WNT5A* in the cells under different conditions. For (**e**) and (**g**), error bars indicate ± SD (n = 3 independent experiments), n.s., $P \geq 0.05$; *, $P < 0.05$; **, $P < 0.01$, ***, $P < 0.001$. For (**b**), (**c**), (**d**), and (**f**), similar results were obtained in three independent experiments.
