# Supplementary material for: Generation and characterization of stable pig pregastrulation epiblast stem cell lines
Source: Cell Res. 2021 Nov 30;32(4):383–400. doi: 10.1038/s41422-021-00592-9 (PMC8976023; doi:10.1038/s41422-021-00592-9)
Supplement: Supplementary file 4 — Supplementary information, Figure S4 [file 41422_2021_592_MOESM4_ESM.pdf]

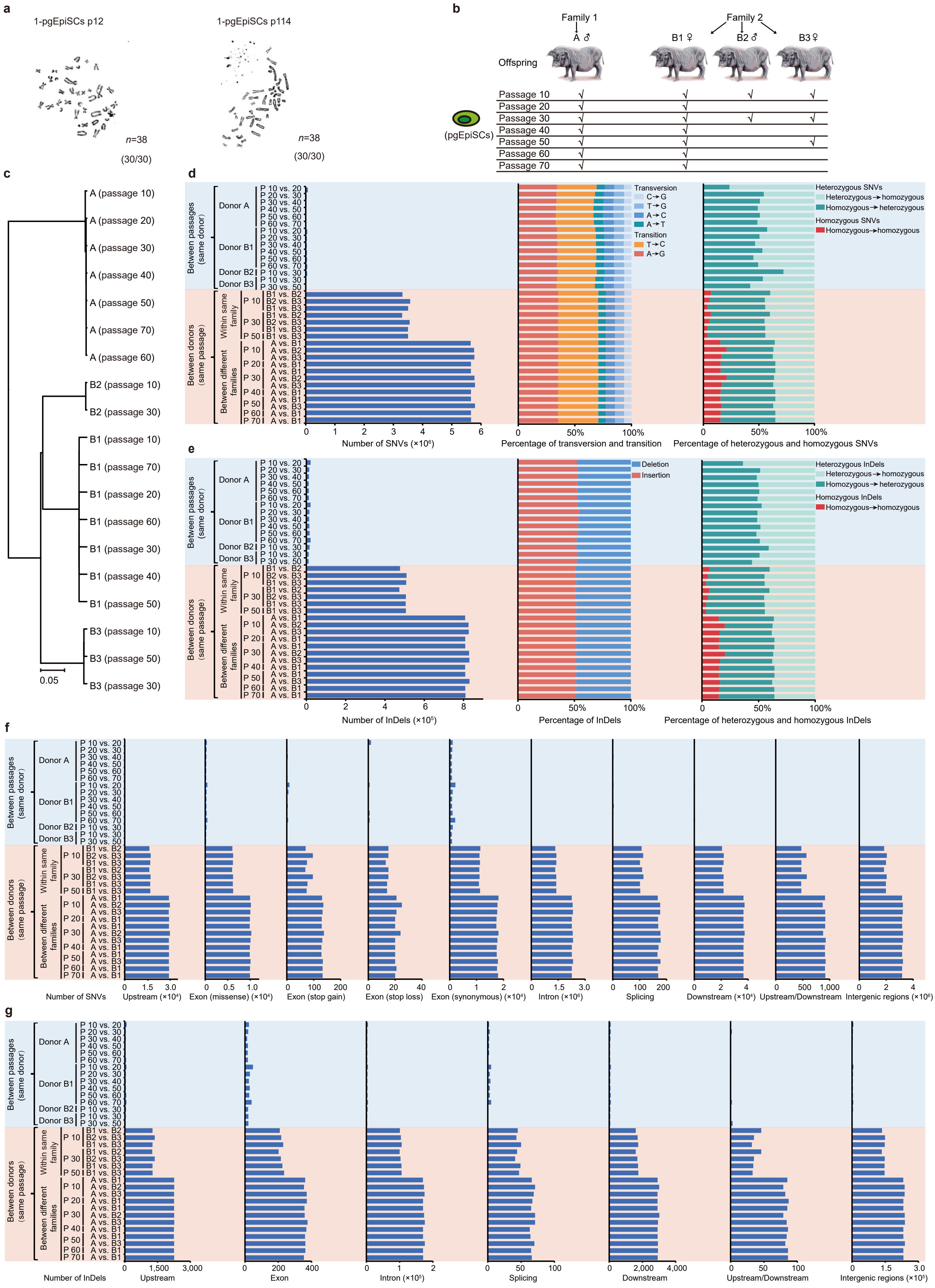

a
1-pgEpiSCs p12
n=38
(30/30)
1-pgEpiSCs p114
n=38
(30/30)
b
Family 1
Family 2
A ♂
B1 ♀
B2 ♂
B3 ♀
Offspring
(pgEpiSCs)
Passage 10
Passage 20
Passage 30
Passage 40
Passage 50
Passage 60
Passage 70
c
A (passage 10)
A (passage 20)
A (passage 30)
A (passage 40)
A (passage 50)
A (passage 70)
A (passage 60)
B2 (passage 10)
B2 (passage 30)
B1 (passage 10)
B1 (passage 70)
B1 (passage 20)
B1 (passage 60)
B1 (passage 30)
B1 (passage 40)
B1 (passage 50)
B3 (passage 10)
B3 (passage 50)
B3 (passage 30)
0.05
d
Between passages (same donor)
Between donors (same passage)
Within same family
Between different families
Donor A
Donor B1
Donor B2
Donor B3
Transversion
C→G
T→G
A→C
A→T
Transition
T→C
A→G
Heterozygous SNVs
Heterozygous→homozygous
Homozygous→heterozygous
Homozygous SNVs
Homozygous→homozygous
Number of SNVs (×10⁶)
Percentage of transversion and transition
Percentage of heterozygous and homozygous SNVs
e
Deletion
Insertion
Heterozygous InDels
Homozygous InDels
Number of InDels (×10⁵)
Percentage of InDels
Percentage of heterozygous and homozygous InDels
f
Number of SNVs
Upstream (×10⁴)
Exon (missense) (×10⁴)
Exon (stop gain)
Exon (stop loss)
Exon (synonymous) (×10⁴)
Intron (×10⁶)
Splicing
Downstream (×10⁴)
Upstream/Downstream
Intergenic regions (×10⁶)
g
Number of InDels
Upstream
Exon
Intron (×10⁵)
Splicing
Downstream
Upstream/Downstream
Intergenic regions (×10⁵)

Fig. S4. Characterization of SNVs and Short InDels (≤30 bp) in the pgEpiSC Lines after Multi-passages and Between Different Donors, Related to Fig. 2

a Karyotype analysis of low and high passage pgEpiSCs. For each cell line, 30 cells at metaphase were examined. **b** Illustration of the whole-genome resequencing of 19 pgEpiSC lines (~24.42 × sequencing depth for each line) derived from four donors (A, B1, B2 and B3). Notably, three donors (i.e., B1, B2 and B3) are full-sibs. **c** Neighbor-joining (NJ) phylogenetic tree of 19 pgEpiSC lines. The scale bar represents *p* distance. **d, e** Number and composition of SNVs (**d**) and InDels (**e**). Compared to a large amount of genetic mutations between pgEpiSCs at same passage but from different donors (between three full-sibs: ~3.46 M SNVs [the Ts/Tv ratio: ~2.41] and ~498.10 K InDels; between different families: ~5.71 M SNVs [the Ts/Tv ratio: ~2.41] and ~816.72 K InDels), the number of mutations between pgEpiSCs from the same donor after multi-passages (~37.98 K SNVs [the Ts/Tv ratio: ~2.13] and ~15.58 K InDels) only account for a small portion (compared with that between two full-sibs, SNVs: ~1.10%; InDels: ~3.13%). Additionally, compared to a fraction of mutations between pgEpiSCs from the same donor after multi-passages are homozygous (SNVs: ~0.08%; InDels: ~0.22%), the occurrence of homozygous mutations is more dramatically increased between pgEpiSCs from different donors (between three full-sibs: ~5.22% of SNVs and ~4.96% of InDels; between donors from different families: ~16.67% of SNVs and ~16.02% of InDels). **f, g** Summary and annotation of SNVs (**f**) and InDels (**g**) in different genomic elements. The package ANNOVAR was used to annotate the location for each SNV and InDel with respect to genes.
