# Supplementary material for: Generation and characterization of stable pig pregastrulation epiblast stem cell lines
Source: Cell Res. 2021 Nov 30;32(4):383–400. doi: 10.1038/s41422-021-00592-9 (PMC8976023; doi:10.1038/s41422-021-00592-9)
Supplement: Supplementary file 5 — Supplementary information, Figure S5 [file 41422_2021_592_MOESM5_ESM.pdf]

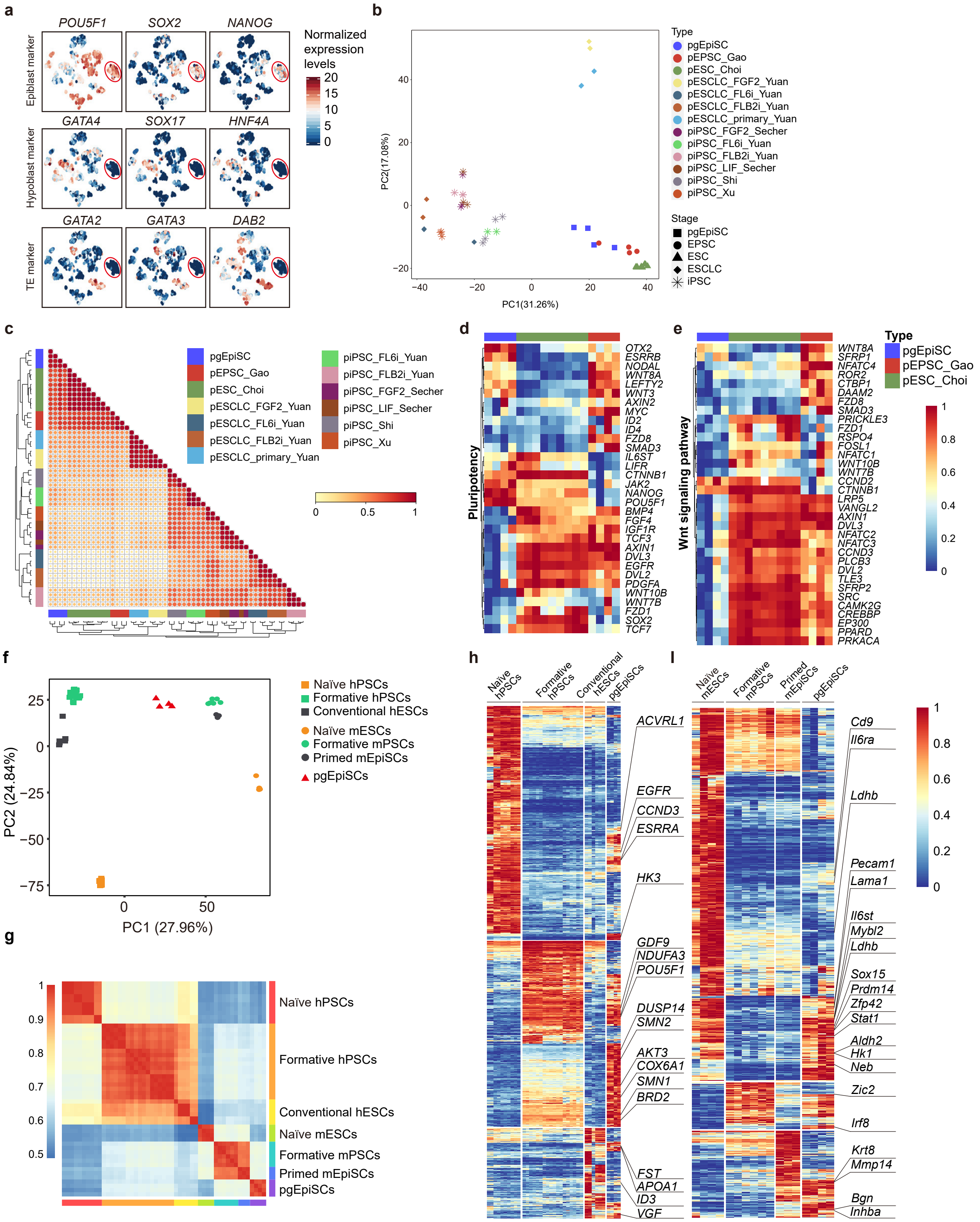
a
POU5F1
SOX2
NANOG
Epiblast marker
GATA4
SOX17
HNF4A
Hypoblast marker
GATA2
GATA3
DAB2
TE marker
Normalized expression levels
b
Type
pgEpiSC
pEPSC_Gao
pESC_Choi
pESCLC_FGF2_Yuan
pESCLC_FL6i_Yuan
pESCLC_FLB2i_Yuan
pESCLC_primary_Yuan
piPSC_FGF2_Secher
piPSC_FL6i_Yuan
piPSC_FLB2i_Yuan
piPSC_LIF_Secher
piPSC_Shi
piPSC_Xu
Stage
pgEpiSC
EPSC
ESC
ESCLC
iPSC
PC1(31.26%)
PC2(17.08%)
c
d
Pluripotency
e
Wnt signaling pathway
Type
pgEpiSC
pEPSC_Gao
pESC_Choi
f
Naïve hPSCs
Formative hPSCs
Conventional hESCs
Naïve mESCs
Formative mPSCs
Primed mEpiSCs
pgEpiSCs
PC1 (27.96%)
PC2 (24.84%)
g
h
Naïve hPSCs
Formative hPSCs
Conventional hESCs
pgEpiSCs
ACVRL1
EGFR
CCND3
ESRRA
HK3
GDF9
NDUFA3
POU5F1
DUSP14
SMN2
AKT3
COX6A1
SMN1
BRD2
FST
APOA1
ID3
VGF
I
Naïve mESCs
Formative mPSCs
Primed mEpiSCs
pgEpiSCs
Cd9
Il6ra
Ldhb
Pecam1
Lama1
Il6st
Mybl2
Ldhb
Sox15
Prdm14
Zfp42
Stat1
Aldh2
Hk1
Neb
Zic2
Irf8
Krt8
Mmp14
Bgn
Inhba

Fig. S5: Transcriptome Characterization of pgEpiSCs, Related to Fig. 4

a Expression ($\log_2$(TPM/10 + 1)) of marker genes representing epiblast, hypoblast and TE are normalized and displayed in t-SNE plots. The gradient ranging from blue to red indicates low to high expression levels. **b** PCA plot of pgEpiSCs and public established pig PSCs, each dot represents one cell line, pgEpiSCs shown more close to pESCs and pEPSCs contrast to iPSCs. **c** Spearman's correlation coefficients of pgEpiSCs and reported established pig PSCs. **d**, **e** Heatmap of the DEGs relevant with pluripotency (**d**) and Wnt signaling pathway (**e**) among pgEpiSCs, pEPSCs and pESCs, pgEpiSCs highly expressed canonical pluripotent markers (*NANOG, POU5F1,* and *OTX2*) and kept an extremely low state of WNT pathway activity. **f** PCA plot of naïve, formative and conventional hPSCs; naïve, formative and primed mPSCs; and pgEpiSCs based on the union of uniquely expressed genes for each PSC. Color represents pluripotency state. Triangles represent pigs, squares represent humans, and circles represent mice. **g** Spearman's correlation coefficients of pgEpiSCs, naïve, formative, and conventional or primed PSCs in human and mouse based on the union of uniquely expressed genes identified within naïve hPSCs, formative hPSCs, conventional hESCs, naïve mESCs, formative mPSCs, and primed mEpiSCs. **h**, **i** Heatmap showing the expression levels of uniquely expressed genes identified within naïve hPSCs, formative hPSCs, and conventional hESCs (**h**) and within naïve mESCs, formative mPSCs, and primed mEpiSCs (**i**), compared with those in pgEpiSCs (Supplementary information, Tables S4b, c). The genes listed are highly expressed in pgEpiSCs.
