# Supplementary material for: Generation and characterization of stable pig pregastrulation epiblast stem cell lines
Source: Cell Res. 2021 Nov 30;32(4):383–400. doi: 10.1038/s41422-021-00592-9 (PMC8976023; doi:10.1038/s41422-021-00592-9)
Supplement: Supplementary file 6 — Supplementary information, Figure S6 [file 41422_2021_592_MOESM6_ESM.pdf]

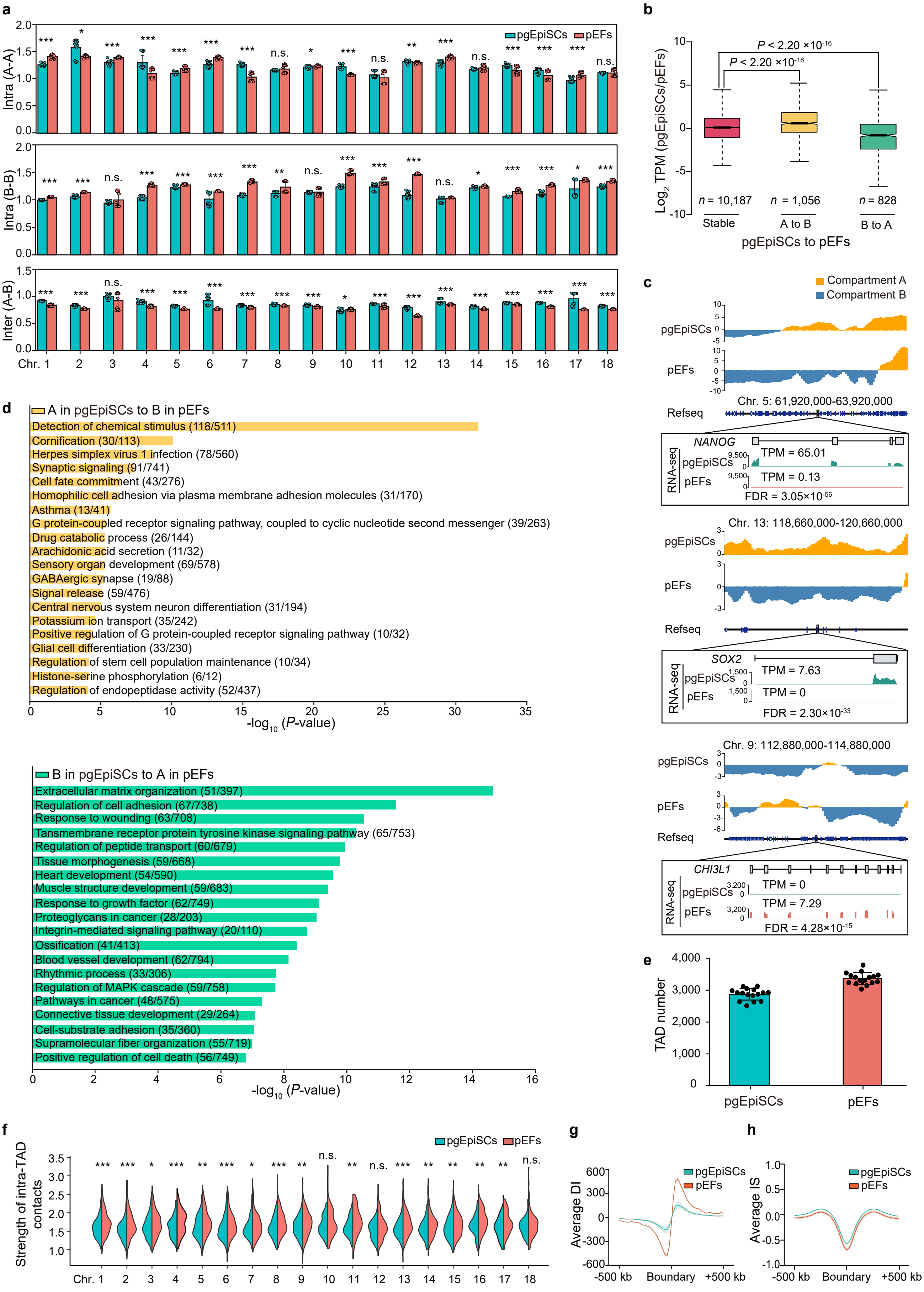
a
pgEpiSCs pEFs
Intra (A-A)
Intra (B-B)
Inter (A-B)
*** * *** *** *** *** *** n.s. * *** n.s. ** *** n.s. *** *** *** n.s.
*** *** n.s. *** *** *** *** ** n.s. *** *** *** n.s. * *** *** * ***
*** *** n.s. *** *** *** *** *** *** * *** *** *** *** *** *** *** ***
Chr. 1 2 3 4 5 6 7 8 9 10 11 12 13 14 15 16 17 18
b
$P < 2.20 \times 10^{-16}$
$P < 2.20 \times 10^{-16}$
Log_2 TPM (pgEpiSCs/pEFs)
n = 10,187
n = 1,056
n = 828
Stable
A to B
B to A
pgEpiSCs to pEFs
c
Compartment A
Compartment B
pgEpiSCs
pEFs
Refseq
Chr. 5: 61,920,000-63,920,000
NANOG
RNA-seq
pgEpiSCs
pEFs
TPM = 65.01
TPM = 0.13
$FDR = 3.05 \times 10^{-56}$
Chr. 13: 118,660,000-120,660,000
SOX2
TPM = 7.63
TPM = 0
$FDR = 2.30 \times 10^{-33}$
Chr. 9: 112,880,000-114,880,000
CHI3L1
TPM = 0
TPM = 7.29
$FDR = 4.28 \times 10^{-15}$
d
A in pgEpiSCs to B in pEFs
Detection of chemical stimulus (118/511)
Cornification (30/113)
Herpes simplex virus 1 infection (78/560)
Synaptic signaling (91/741)
Cell fate commitment (43/276)
Homophilic cell adhesion via plasma membrane adhesion molecules (31/170)
Asthma (13/41)
G protein-coupled receptor signaling pathway, coupled to cyclic nucleotide second messenger (39/263)
Drug catabolic process (26/144)
Arachidonic acid secretion (11/32)
Sensory organ development (69/578)
GABAergic synapse (19/88)
Signal release (59/476)
Central nervous system neuron differentiation (31/194)
Potassium ion transport (35/242)
Positive regulation of G protein-coupled receptor signaling pathway (10/32)
Glial cell differentiation (33/230)
Regulation of stem cell population maintenance (10/34)
Histone-serine phosphorylation (6/12)
Regulation of endopeptidase activity (52/437)
$-\log_{10}$ (P-value)
B in pgEpiSCs to A in pEFs
Extracellular matrix organization (51/397)
Regulation of cell adhesion (67/738)
Response to wounding (63/708)
Tansmembrane receptor protein tyrosine kinase signaling pathway (65/753)
Regulation of peptide transport (60/679)
Tissue morphogenesis (59/668)
Heart development (54/590)
Muscle structure development (59/683)
Response to growth factor (62/749)
Proteoglycans in cancer (28/203)
Integrin-mediated signaling pathway (20/110)
Ossification (41/413)
Blood vessel development (62/794)
Rhythmic process (33/306)
Regulation of MAPK cascade (59/758)
Pathways in cancer (48/575)
Connective tissue development (29/264)
Cell-substrate adhesion (35/360)
Supramolecular fiber organization (55/719)
Positive regulation of cell death (56/749)
$-\log_{10}$ (P-value)
e
TAD number
pgEpiSCs
pEFs
f
pgEpiSCs pEFs
Strength of intra-TAD contacts
*** *** * *** ** *** * *** ** n.s. ** n.s. *** ** ** ** ** n.s.
Chr. 1 2 3 4 5 6 7 8 9 10 11 12 13 14 15 16 17 18
g
Average DI
pgEpiSCs
pEFs
-500 kb
Boundary
+500 kb
h
Average IS
pgEpiSCs
pEFs
-500 kb
Boundary
+500 kb

Fig. S6: Nuclear Architectures Between pgEpiSCs and pEFs, Related to Fig. 5

a Comparison of compartment contacts across 18 autosomes at 20-kb resolution in each of 16 Hi-C maps. The dramatic decrease of compartmentalization strength (AA × BB/AB^2) in pgEpiSCs compared to pEFs (Fig. 5e) is not only attributed to the increased inter-compartment contacts in pgEpiSCs (pgEpiSCs compared to pEFs: 0.86/0.79, $P = 1.12 \times 10^{-3}$, Wilcoxon rank-sum test), but also resulted from more reduced contacts within the B compartment (pgEpiSCs compared to pEFs: 1.11/1.23, $P = 2.97 \times 10^{-3}$, Wilcoxon rank-sum test) and slightly increased contacts within the A compartment (pgEpiSCs compared to pEFs: 1.22/1.20, $P = 0.58$, Wilcoxon rank-sum test) in pgEpiSCs. **b** Expression changes of genes located in regions exhibiting compartment A/B switching between pgEpiSCs and pEFs. Genes detected in at least one cell type are shown. **c** Examples of genes located in regions that exhibited compartment A/B switching between pgEpiSCs and pEFs at loci of *NANOG* and *SOX2* (from A status in pgEpiSCs to B status in pEFs, and upregulated in pgEpiSCs), and *CHI3L1* (from B status in pgEpiSCs to A status in pEFs, and downregulated in pgEpiSCs). The Benjamini-Hochberg adjusted FDRs were calculated using DEseq2 tool (version 1.28.1) to estimate statistical significance. **d** Functional enrichment for 2 817 genes from A status in pgEpiSCs to B status in pEFs (yellow bars), and 1 164 genes from B status in pgEpiSCs to A status in pEFs (green bars). The top functional terms of Metascape (https://metascape.org; see Materials and methods) summary gene set in each enriched cluster are shown, with the constraint of showing no more than 20 terms. The number after each term represents the hit genes out of total genes in this term. **e** The number of TADs in each Hi-C map. **f** Violin plots showing examples of the strength of intra-TAD contacts in pgEpiSC-1-B and pEF-1-G. **g**, **h** Average directionality index (DI) (**g**) and average insulation score (IS) (**h**) in a 1 Mb region centered on shared TAD boundaries between pgEpiSCs and pEFs. Lines show mean values, while shaded ribbons represent SD. Note the spatial segregation between neighboring TADs in pgEpiSCs were weakened compared to that in PEFs (113.34/320.95 for DI, $P = 1.44 \times 10^{-4}$, and -0.05/-0.12 for IS, $P = 2.59 \times 10^{-3}$, Wilcoxon rank-sum test). For (**a**), (**b**), (**f**), (**g**), and (**h**), values are means ± S.D. Statistical significance was calculated by Wilcoxon rank-sum test (n.s., $P \geq 0.05$; *, $P < 0.05$; **, $P < 0.01$, ***, $P < 0.001$).
