# Supplementary material for: Generation and characterization of stable pig pregastrulation epiblast stem cell lines
Source: Cell Res. 2021 Nov 30;32(4):383–400. doi: 10.1038/s41422-021-00592-9 (PMC8976023; doi:10.1038/s41422-021-00592-9)
Supplement: Supplementary file 7 — Supplementary information, Figure S7 [file 41422_2021_592_MOESM7_ESM.pdf]

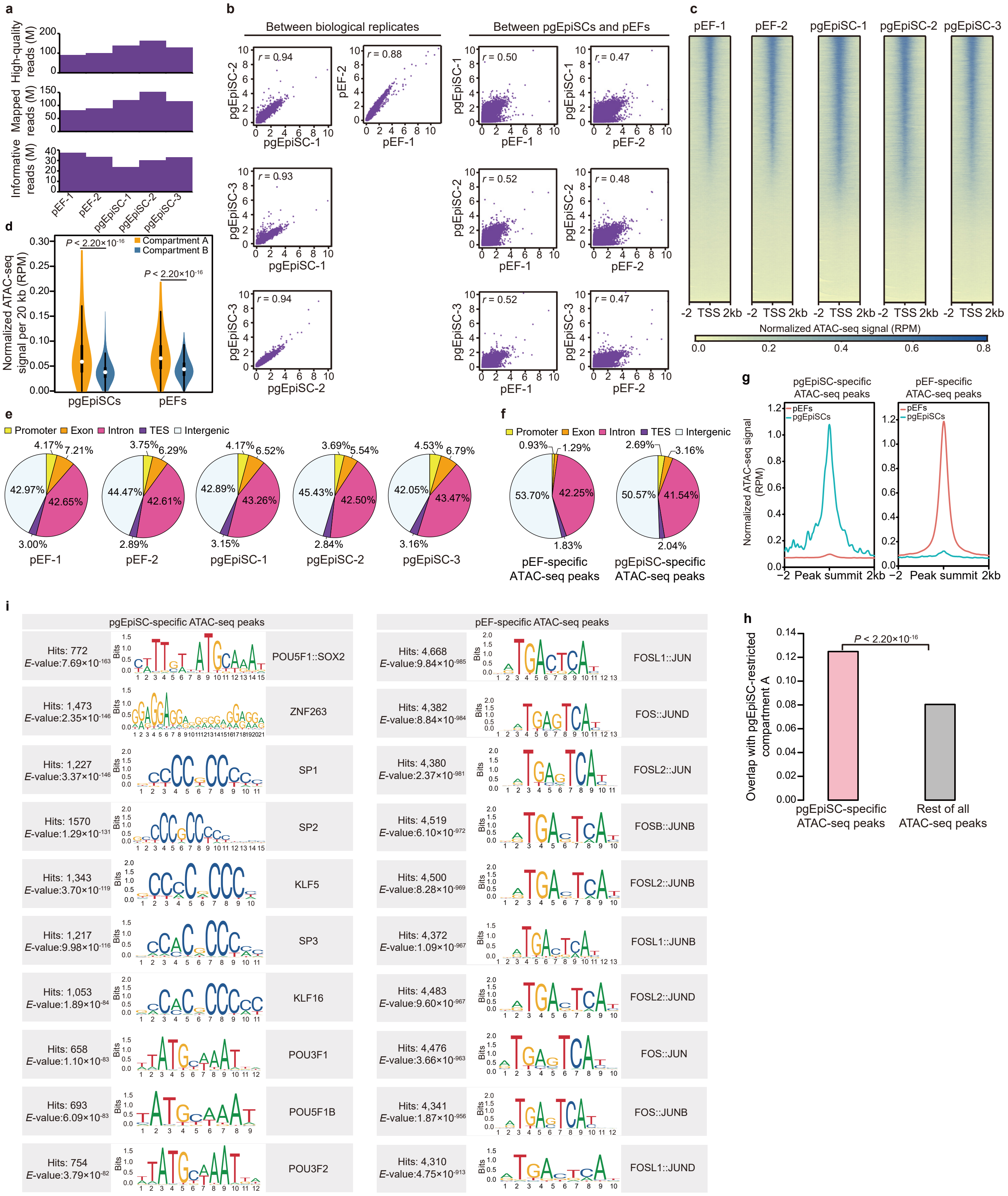

a
High-quality reads (M)
Mapped reads (M)
Informative reads (M)
pEF-1
pEF-2
pgEpiSC-1
pgEpiSC-2
pgEpiSC-3
b
Between biological replicates
Between pgEpiSCs and pEFs
r = 0.94
r = 0.88
r = 0.50
r = 0.47
r = 0.93
r = 0.52
r = 0.48
r = 0.94
r = 0.52
r = 0.47
c
-2 TSS 2kb
Normalized ATAC-seq signal (RPM)
d
Normalized ATAC-seq signal per 20 kb (RPM)
P < 2.20×10^-16
Compartment A
Compartment B
pgEpiSCs
pEFs
e
Promoter
Exon
Intron
TES
Intergenic
4.17%
7.21%
42.97%
42.65%
3.00%
3.75%
6.29%
44.47%
42.61%
2.89%
4.17%
6.52%
42.89%
43.26%
3.15%
3.69%
5.54%
45.43%
42.50%
2.84%
4.53%
6.79%
42.05%
43.47%
3.16%
f
0.93%
1.29%
53.70%
42.25%
1.83%
pEF-specific ATAC-seq peaks
2.69%
3.16%
50.57%
41.54%
2.04%
pgEpiSC-specific ATAC-seq peaks
g
pgEpiSC-specific ATAC-seq peaks
pEF-specific ATAC-seq peaks
Normalized ATAC-seq signal (RPM)
pEFs
pgEpiSCs
-2 Peak summit 2kb
h
Overlap with pgEpiSC-restricted compartment A
P < 2.20×10^-16
pgEpiSC-specific ATAC-seq peaks
Rest of all ATAC-seq peaks
i
pgEpiSC-specific ATAC-seq peaks
Hits: 772 E-value:7.69×10^-163 POU5F1::SOX2
Hits: 1,473 E-value:2.35×10^-146 ZNF263
Hits: 1,227 E-value:3.37×10^-146 SP1
Hits: 1570 E-value:1.29×10^-131 SP2
Hits: 1,343 E-value:3.70×10^-119 KLF5
Hits: 1,217 E-value:9.98×10^-116 SP3
Hits: 1,053 E-value:1.89×10^-84 KLF16
Hits: 658 E-value:1.10×10^-83 POU3F1
Hits: 693 E-value:6.09×10^-83 POU5F1B
Hits: 754 E-value:3.79×10^-82 POU3F2
pEF-specific ATAC-seq peaks
Hits: 4,668 E-value:9.84×10^-985 FOSL1::JUN
Hits: 4,382 E-value:8.84×10^-984 FOS::JUND
Hits: 4,380 E-value:2.37×10^-981 FOSL2::JUN
Hits: 4,519 E-value:6.10×10^-972 FOSB::JUNB
Hits: 4,500 E-value:8.28×10^-969 FOSL2::JUNB
Hits: 4,372 E-value:1.09×10^-967 FOSL1::JUNB
Hits: 4,483 E-value:9.60×10^-967 FOSL2::JUND
Hits: 4,476 E-value:3.66×10^-963 FOS::JUN
Hits: 4,341 E-value:1.87×10^-956 FOS::JUNB
Hits: 4,310 E-value:4.75×10^-913 FOSL1::JUND
Bits

Fig. S7: Chromatin Accessibility Between pgEpiSCs and pEFs, Related to Fig. 6

a Data summary of ATAC-seq. **b** Scatterplots showing pairwise Pearson's *r* of normalized ATAC-seq signal (reads per million; RPM) between five ATAC-seq data. The genome-wide ATAC-seq signal was highly reproducible between biological replicates (average Pearson's $r = 0.92$), but are more dissimilar between pgEpiSCs and pEFs (average Pearson's $r = 0.49$). **c** Heatmaps depicting the enrichment of normalized ATAC-seq signal centered on TSS (ordered by signal intensity). **d** Normalized ATAC-seq signal in each 20 kb bins for compartment A (orange) or B (blue) regions of pgEpiSCs and pEFs, respectively. Statistical significance was calculated by Wilcoxon rank-sum test. **e, f** Distribution of ATAC-seq peaks (**e**) and cell type-specific ATAC-seq peaks (**f**) relative to genomic features. The peaks were divided into five distinct categories based on 1 bp overlap with features (i.e., promoter, exon, intron, TES, and intergenic regions). Notably, we found ~4.06% peaks are localized in promoter (**e**), which are depleted in cell type-specific peaks (~1.81%) (**f**), thus suggesting that cell type-specific peaks are more likely to delineate enhancers. **g** Average of normalized ATAC-seq signal (RPM) in a 4 kb region centered on cell type-specific peaks between pgEpiSCs and pEFs. **h** Overlap of pgEpiSC-specific ATAC-seq peaks or the rest of all peaks with pgEpiSC-restricted compartment A regions. Statistical significance was determined by two proportions test. **i** Top ten enriched transcription factor (TF) binding motifs within cell type-specific ATAC-seq peaks, as identified by JASPAR database [69]. As expected, the pgEpiSCs-specific peaks are mainly enriched in motifs corresponding to the common pluripotent transcription factors (TFs) (typically, *POU5F1*, *SOX2*, *KLF5*, *POU3F1*, *SP1* and *SP3*). In contrast, the pEF-specific peaks are mainly enriched in motifs corresponding to the FOS and JUN family (two subfamilies of activator protein 1).
