# Supplementary material for: Generation and characterization of stable pig pregastrulation epiblast stem cell lines
Source: Cell Res. 2021 Nov 30;32(4):383–400. doi: 10.1038/s41422-021-00592-9 (PMC8976023; doi:10.1038/s41422-021-00592-9)
Supplement: Supplementary file 8 — Supplementary information, Figure S8 [file 41422_2021_592_MOESM8_ESM.pdf]

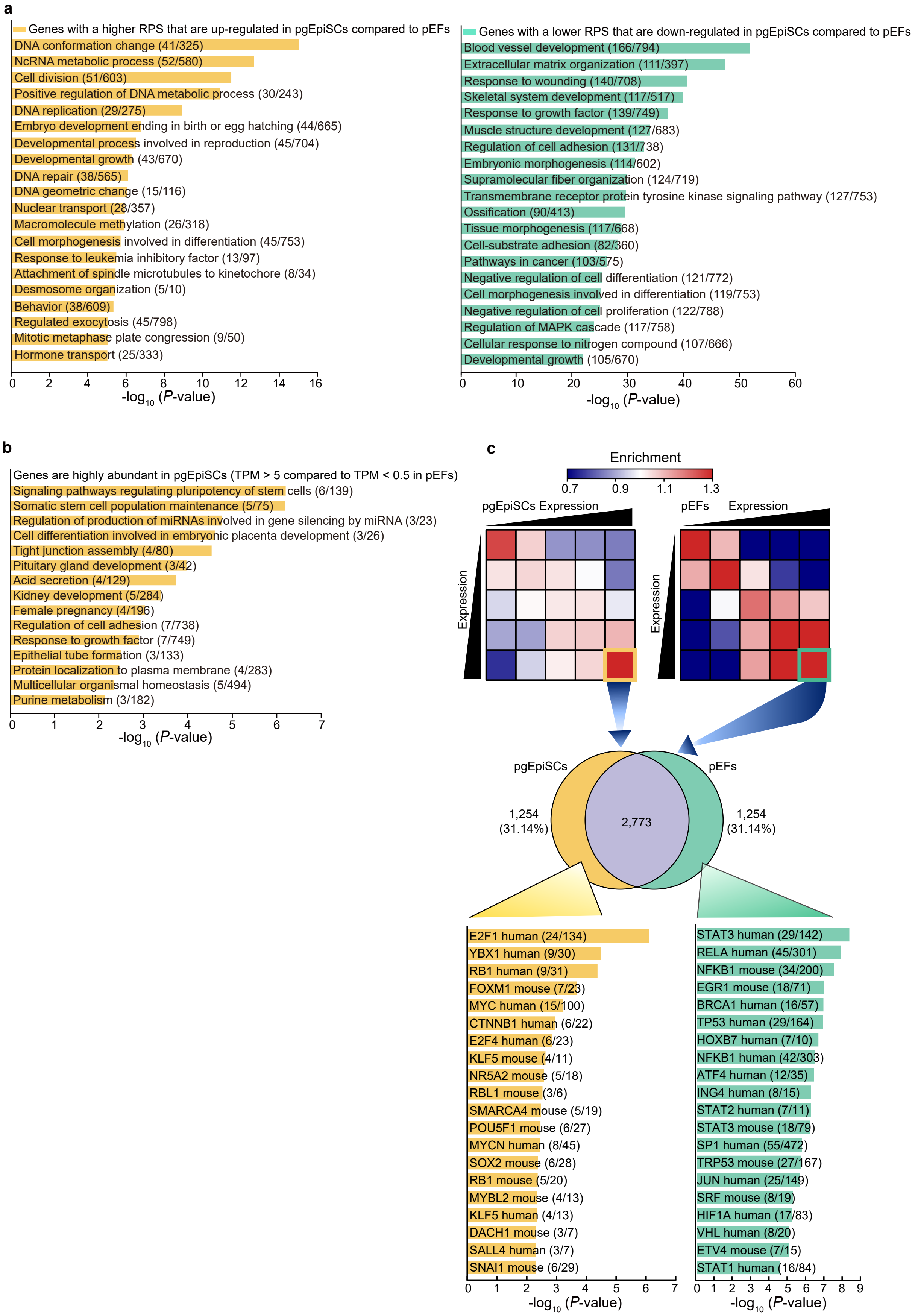

a
Genes with a higher RPS that are up-regulated in pgEpiSCs compared to pEFs
DNA conformation change (41/325)
NcRNA metabolic process (52/580)
Cell division (51/603)
Positive regulation of DNA metabolic process (30/243)
DNA replication (29/275)
Embryo development ending in birth or egg hatching (44/665)
Developmental process involved in reproduction (45/704)
Developmental growth (43/670)
DNA repair (38/565)
DNA geometric change (15/116)
Nuclear transport (28/357)
Macromolecule methylation (26/318)
Cell morphogenesis involved in differentiation (45/753)
Response to leukemia inhibitory factor (13/97)
Attachment of spindle microtubules to kinetochore (8/34)
Desmosome organization (5/10)
Behavior (38/609)
Regulated exocytosis (45/798)
Mitotic metaphase plate congression (9/50)
Hormone transport (25/333)
-log10 (P-value)
Genes with a lower RPS that are down-regulated in pgEpiSCs compared to pEFs
Blood vessel development (166/794)
Extracellular matrix organization (111/397)
Response to wounding (140/708)
Skeletal system development (117/517)
Response to growth factor (139/749)
Muscle structure development (127/683)
Regulation of cell adhesion (131/738)
Embryonic morphogenesis (114/602)
Supramolecular fiber organization (124/719)
Transmembrane receptor protein tyrosine kinase signaling pathway (127/753)
Ossification (90/413)
Tissue morphogenesis (117/668)
Cell-substrate adhesion (82/360)
Pathways in cancer (103/575)
Negative regulation of cell differentiation (121/772)
Cell morphogenesis involved in differentiation (119/753)
Negative regulation of cell proliferation (122/788)
Regulation of MAPK cascade (117/758)
Cellular response to nitrogen compound (107/666)
Developmental growth (105/670)
-log10 (P-value)
b
Genes are highly abundant in pgEpiSCs (TPM > 5 compared to TPM < 0.5 in pEFs)
Signaling pathways regulating pluripotency of stem cells (6/139)
Somatic stem cell population maintenance (5/75)
Regulation of production of miRNAs involved in gene silencing by miRNA (3/23)
Cell differentiation involved in embryonic placenta development (3/26)
Tight junction assembly (4/80)
Pituitary gland development (3/42)
Acid secretion (4/129)
Kidney development (5/284)
Female pregnancy (4/196)
Regulation of cell adhesion (7/738)
Response to growth factor (7/749)
Epithelial tube formation (3/133)
Protein localization to plasma membrane (4/283)
Multicellular organismal homeostasis (5/494)
Purine metabolism (3/182)
-log10 (P-value)
c
Enrichment
0.7 0.9 1.1 1.3
pgEpiSCs Expression
pEFs Expression
Expression
pgEpiSCs
pEFs
1,254 (31.14%)
2,773
1,254 (31.14%)
E2F1 human (24/134)
YBX1 human (9/30)
RB1 human (9/31)
FOXM1 mouse (7/23)
MYC human (15/100)
CTNNB1 human (6/22)
E2F4 human (6/23)
KLF5 mouse (4/11)
NR5A2 mouse (5/18)
RBL1 mouse (3/6)
SMARCA4 mouse (5/19)
POU5F1 mouse (6/27)
MYCN human (8/45)
SOX2 mouse (6/28)
RB1 mouse (5/20)
MYBL2 mouse (4/13)
KLF5 human (4/13)
DACH1 mouse (3/7)
SALL4 human (3/7)
SNAI1 mouse (6/29)
-log10 (P-value)
STAT3 human (29/142)
RELA human (45/301)
NFKB1 mouse (34/200)
EGR1 mouse (18/71)
BRCA1 human (16/57)
TP53 human (29/164)
HOXB7 human (7/10)
NFKB1 human (42/303)
ATF4 human (12/35)
ING4 human (8/15)
STAT2 human (7/11)
STAT3 mouse (18/79)
SP1 human (55/472)
TRP53 mouse (27/167)
JUN human (25/149)
SRF mouse (8/19)
HIF1A human (17/83)
VHL human (8/20)
ETV4 mouse (7/15)
STAT1 human (16/84)
-log10 (P-value)

Fig. S8: Additional Spatial Regulatory Circuitry of the Transcription Between pgEpiSCs and pEFs, Related to Fig. 6

a Functional enrichment for the 2 480 genes that exhibit the same relationships of changes between expression (log_2 fold change [FC] >1, FDR < 0.05) and RPS (*i.e.*, higher RPS being upregulated, and *vice versa*). In pgEpiSCs, 875 genes are up-regulated (yellow bars); whereas 1 605 genes are up-regulated in pEFs (green bars). The top functional terms of Metascape (https://metascape.org; see Materials and methods) summary gene set in each enriched cluster are shown, with the constraint of showing no more than 20 terms. Numbers after each term represent the hit genes out of total genes in this term. **b** Functional enrichment for the co-variation genes (*i.e.*, genes exhibiting the same relationships of changes between expression and RPS) that were specifically expressed in pgEpiSCs (average TPM > 5) compared to pEFs (average TPM < 0.5) (n = 75). Numbers after each term represents the hit genes out of total genes in this term. **c** Comparison of promoter-promoter interactions between pgEpiSCs and pEFs. Top panels: enrichment of interactions between promoters from different expression categories in pgEpiSCs (left) and pEFs (right). Middle panels: Venn diagram showing the distribution of the top 20% of genes with the highest expression level in pgEpiSCs and pEFs. Bottom panels: functional enrichment for genes specifically in pgEpiSCs (left) and pEFs (right). The pig genes were mapped to their human orthologs. Transcription factors (TFs) enriched in cell type specific genes were identified using the results section 'TRRUST TFs 2019' from the Enrichr web server [80] (http://amp.pharm.mssm.edu/Enrichr). The TFs with P < 0.01 are depicted in bar plots, with the constraint that no more than 20 TFs are shown. The numbers after each TF represent the hit genes out of total target genes for this TF.
