# Supplementary material for: Generation and characterization of stable pig pregastrulation epiblast stem cell lines
Source: Cell Res. 2021 Nov 30;32(4):383–400. doi: 10.1038/s41422-021-00592-9 (PMC8976023; doi:10.1038/s41422-021-00592-9)
Supplement: Supplementary file 9 — Supplementary information, Figure S9 [file 41422_2021_592_MOESM9_ESM.pdf]

a b c

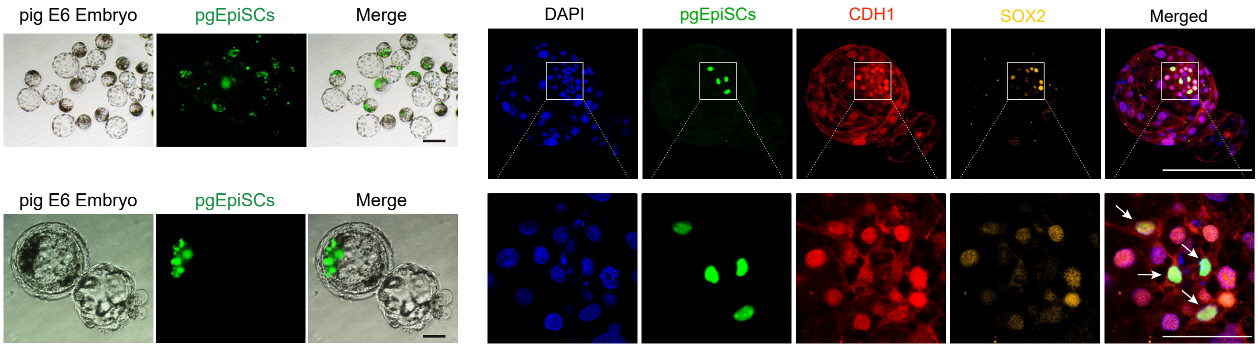

d

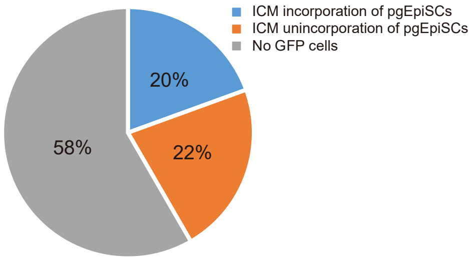

e

Embryo Type	No. Pregnancy	No. Transferred	No. Dissected (Embryonic Day)	No. GFP$^+$
SCNT	2	503	135 (E10)	0
SCNT	4	528	26 (E21~23)	0

Fig. S9: Evaluation of Chimeric Ability of The pgEpiSCs

a, **b** Morphological observation of chimeric embryos. a) Scale bar, 200 μm; b) Scale bar, 50 μm. **c** Representative immunofluorescence images showing ICM incorporation of pgEpiSCs. DAPI (blue), pgEpiSCs (green), CDH1 (red) and SOX2 (yellow). Up, scale bar, 200 μm; bottom, scale bar, 50 μm. Similar results were obtained in three independent experiments. **d** The rate of ICM incorporation of pgEpiSCs. A total of 36 embryos from 2 independent experiments were counted. **e** Statistics of experimental results of chimeric embryo *in vivo* transplantation.
