# Supplementary material for: Generation and characterization of stable pig pregastrulation epiblast stem cell lines
Source: Cell Res. 2021 Nov 30;32(4):383–400. doi: 10.1038/s41422-021-00592-9 (PMC8976023; doi:10.1038/s41422-021-00592-9)
Supplement: Supplementary file 10 — Supplementary information, Figure S10 [file 41422_2021_592_MOESM10_ESM.pdf]

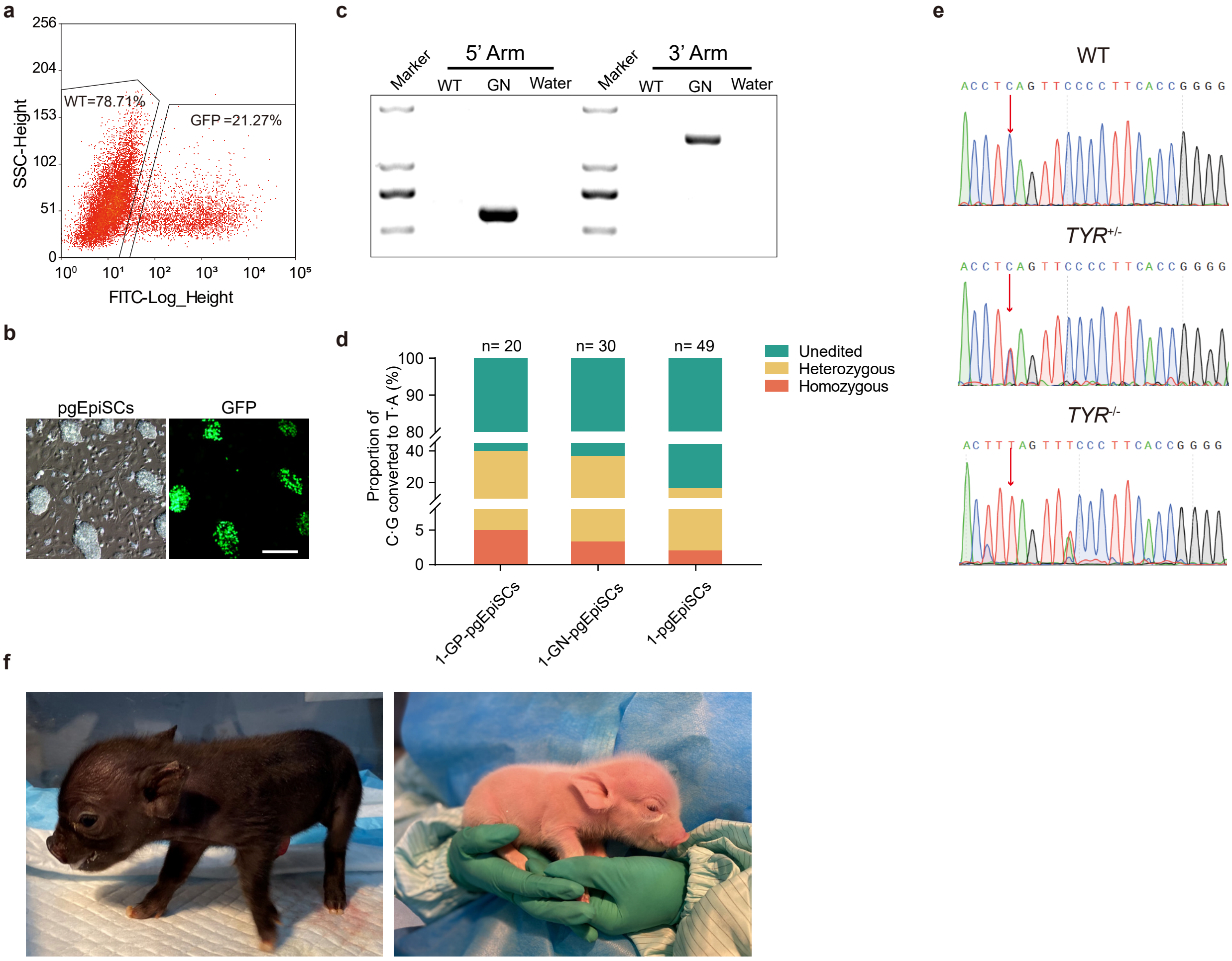
a
WT=78.71%
GFP =21.27%
SSC-Height
FITC-Log_Height
b
pgEpiSCs
GFP
c
Marker
5' Arm
WT
GN
Water
3' Arm
d
n= 20
n= 30
n= 49
Unedited
Heterozygous
Homozygous
Proportion of C·G converted to T·A (%)
1-GP-pgEpiSCs
1-GN-pgEpiSCs
1-pgEpiSCs
e
WT
TYR+/-
TYR-/-
f

Fig. S10: Cloned Piglets from pgEpiSCs with Multiple Gene Edits, Related to Fig. 7

a Transfection efficiency of pgEpiSCs detected by GFP positive ratio using flow cytometry. **b** Morphology and fluorescence of the GFP-pgEpiSC colonies, scale bar, 200 μm. **c** Identification of NANOG-tdTomato knock-in by PCR. “GN” represents a GFP-positive NANOG-tdTomato konck-in pgEpiSC line. **d** Statistics of unedited, homozygous, and heterozygous ratios for *TYR* gene C to T mutation in WT pgEpiSCs (1-pgEpiSCs) and gene-modified pgEpiSCs (1-GP-pgEpiSCs and 1-GN-pgEpiSCs). **e** Representative DNA sequencing analysis of the C to T mutation site in *TYR* gene for WT, heterozygous, and homozygous mutation pgEpiSCs. **f** A representative cloned piglet produced using WT pgEpiSCs as donor cells (left) showing black coat color and a representative cloned piglet produced from GNT-pgEpiSCs (right) showing the phenotype of albinism with white coat color.
