# Supplementary material for: Generation and characterization of stable pig pregastrulation epiblast stem cell lines
Source: Cell Res. 2021 Nov 30;32(4):383–400. doi: 10.1038/s41422-021-00592-9 (PMC8976023; doi:10.1038/s41422-021-00592-9)
Supplement: Supplementary file 11 — Supplementary information, Data S1 [file 41422_2021_592_MOESM11_ESM.pdf]

DATA S1-I

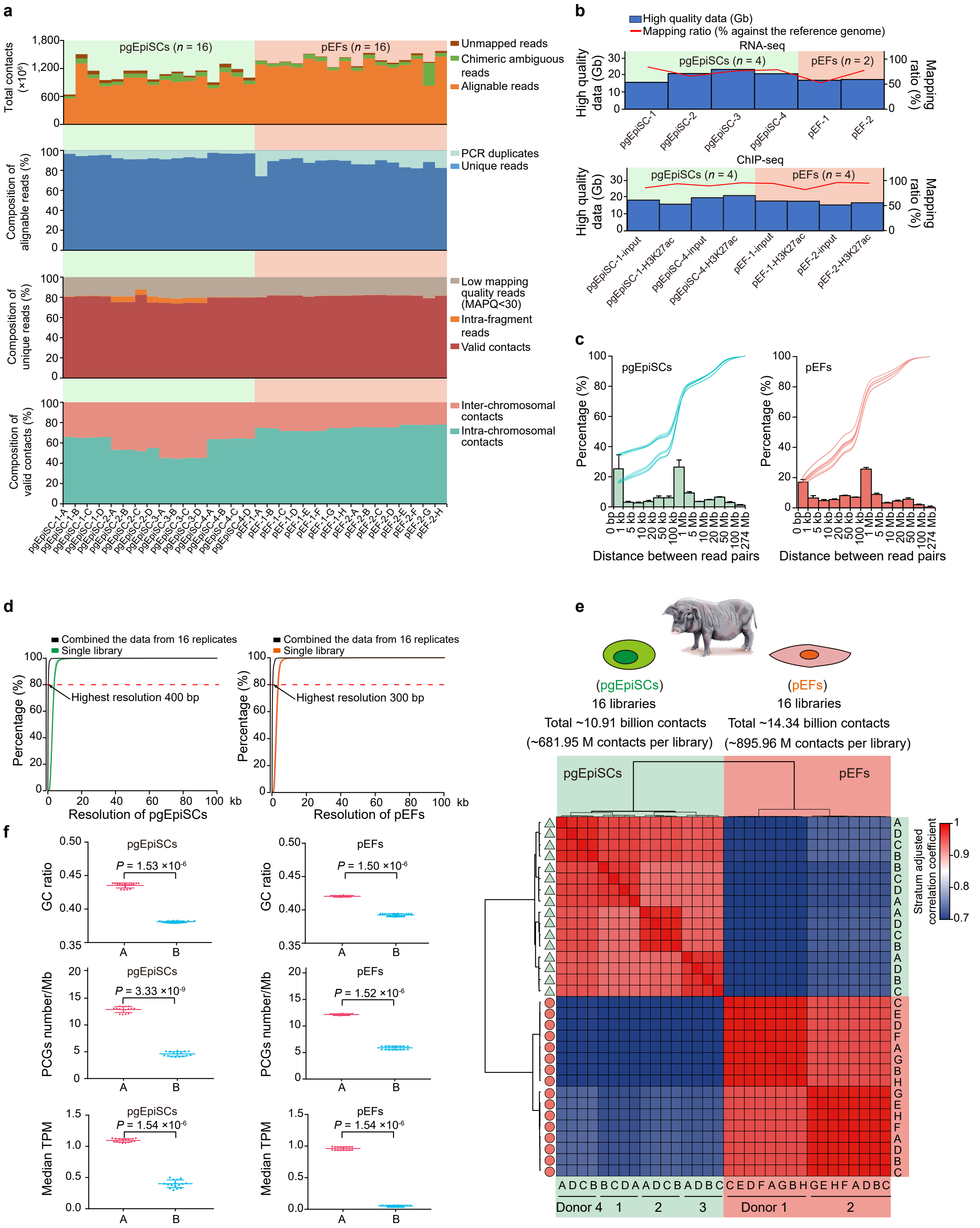

a
pgEpiSCs (n = 16)
pEFs (n = 16)
Total contacts (×10⁶)
Unmapped reads
Chimeric ambiguous reads
Alignable reads
Composition of alignable reads (%)
PCR duplicates
Unique reads
Composition of unique reads (%)
Low mapping quality reads (MAPQ<30)
Intra-fragment reads
Valid contacts
Composition of valid contacts (%)
Inter-chromosomal contacts
Intra-chromosomal contacts
b
High quality data (Gb)
Mapping ratio (% against the reference genome)
RNA-seq
pgEpiSCs (n = 4)
pEFs (n = 2)
ChIP-seq
pgEpiSCs (n = 4)
pEFs (n = 4)
c
pgEpiSCs
pEFs
Percentage (%)
Distance between read pairs
d
Combined the data from 16 replicates
Single library
Highest resolution 400 bp
Highest resolution 300 bp
Resolution of pgEpiSCs
Resolution of pEFs
e
(pgEpiSCs)
16 libraries
Total ~10.91 billion contacts
(~681.95 M contacts per library)
(pEFs)
16 libraries
Total ~14.34 billion contacts
(~895.96 M contacts per library)
Stratum adjusted correlation coefficient
Donor 4
Donor 1
f
GC ratio
PCGs number/Mb
Median TPM
P = 1.53 ×10⁻⁶
P = 1.50 ×10⁻⁶
P = 3.33 ×10⁻⁹
P = 1.52 ×10⁻⁶
P = 1.54 ×10⁻⁶
P = 1.54 ×10⁻⁶

Data S1: Additional Hi-C Maps, rRNA-depleted RNA-seq and ChIP-seq (H3K27ac), Relevant to Main Findings, Related to Fig. 5

Data S1-I: Hi-C Maps, RNA-seq and ChIP-seq (H3K27ac) of pgEpiSCs and pEFs

a Data summary of 32 Hi-C data. We separately constructed 16 Hi-C libraries for pgEpiSCs (a total of ~10.9-billion valid contacts with a depth of ~682 M contacts per library) and pEFs (a total of ~14.3-billion valid contacts with a depth of ~896 M contacts per library). The intra-chromosomal read pairs (~531.06 M contacts, or 66.01% of valid contacts per library) are the dominant interactions compared to inter-chromosomal read pairs. **b** Data summary of rRNA-depleted RNA-seq (top panel; ~126 M 150-bp paired-end high-quality reads were generated for each library) and ChIP-seq (H3K27ac) (bottom panel; ~115.78 M high-quality reads for each library). **c** Insert size distribution of intra-chromosomal contacts. The curves represent the cumulative percentages in each library. Note that ~86.00% of intra-chromosomal contacts mainly occurred within 10 Mb. **d** Evaluation of Hi-C resolution. The curve represents the percentage of bins with more than 1 000 intra-chromosomal contacts at different kb resolutions. According to a previously described criterion that the smallest bin size is where 80% of bins have at least 1 000 intra-chromosomal contacts [1], our Hi-C data of pgEpiSCs and pEFs achieved resolution of at least 5-kb for a single library, and even achieved respective resolutions of 400 bp and 300 bp for pgEpiSCs and pEFs after combining the data from replicates. **e** Estimated interrelationships between 32 intra-chromosomal Hi-C maps at 100-kb resolution of pgEpiSCs (four libraries [A to D] for each of four donors; 16 green triangles) and pEFs (eight libraries [A to G] for each of two donors; 16 red circles) using the pairwise stratum adjusted correlation coefficient (SCC) as implemented in the R package HiCRep [2]. We generated the normalized intra-chromosomal contact maps at 100-kb resolution for each of 32 libraries (~52 884 contacts in each 100-kb bin), which are highly reproducible between maps derived from the same donor of pgEpiSCs (median SCC =0.98) and pEFs (SCC=0.99), followed by between four donors of pgEpiSCs (SCC = 0.95) and two donors of pEFs (SCC = 0.96), but are more dissimilar between pgEpiSCs and pEFs (SCC = 0.71). **f** Compared to the compartment B regions (48.11% of genome in pgEpiSCs and 52.50% of genome in pEFs), the compartment A regions (51.89% of genome in pgEpiSCs and 47.50% of genome in pEFs) are more GC-rich, gene-rich, and have higher transcriptional activities. Statistical significance was calculated by Wilcoxon rank-sum test.

DATA S1-II

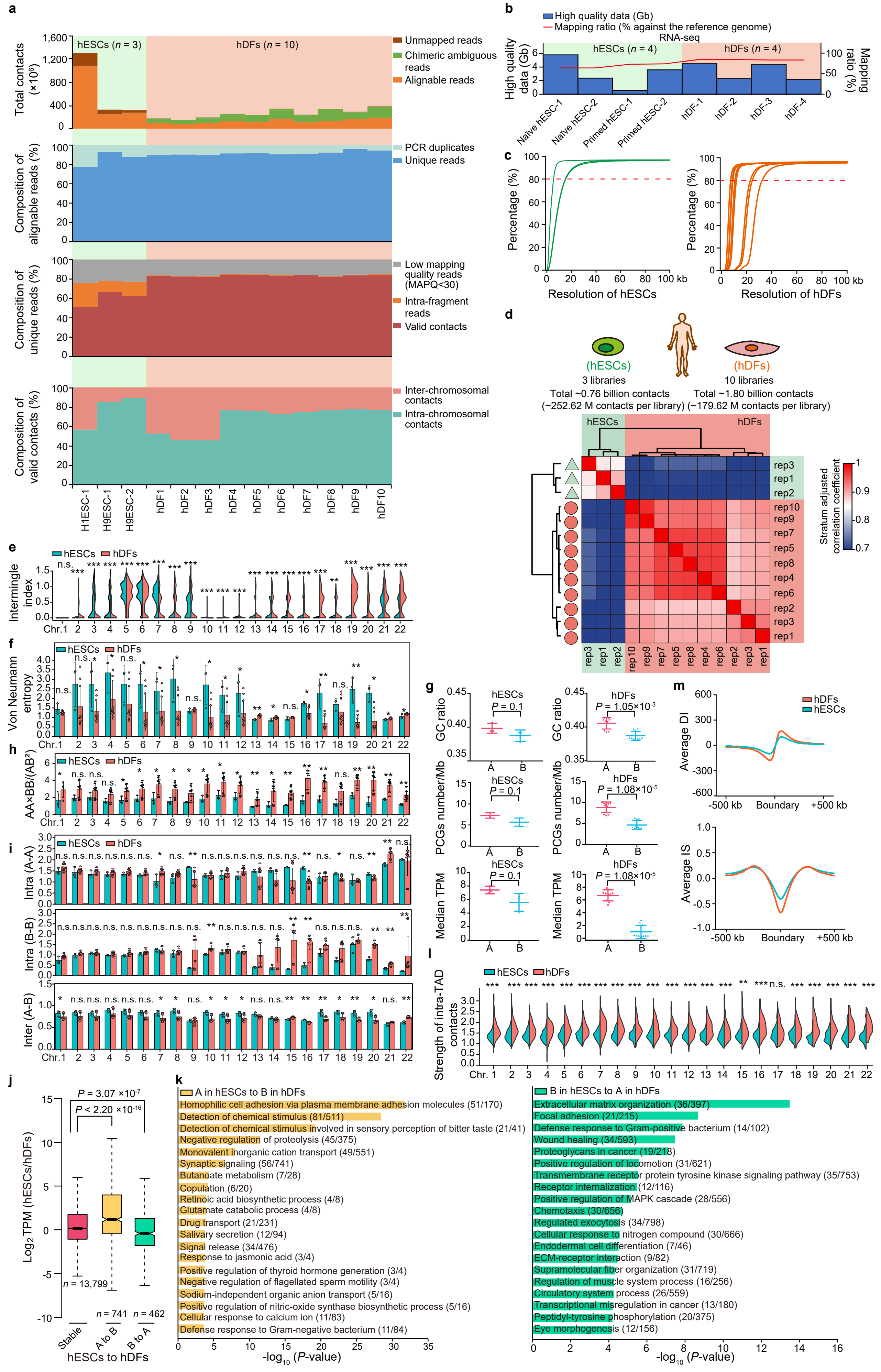
a
hESCs (n = 3)
hDFs (n = 10)
Total contacts (×10^6)
Unmapped reads
Chimeric ambiguous reads
Alignable reads
Composition of alignable reads (%)
PCR duplicates
Unique reads
Composition of unique reads (%)
Low mapping quality reads (MAPQ<30)
Intra-fragment reads
Valid contacts
Composition of valid contacts (%)
Inter-chromosomal contacts
Intra-chromosomal contacts
H1ESC-1
H9ESC-1
H9ESC-2
hDF1
hDF2
hDF3
hDF4
hDF5
hDF6
hDF7
hDF8
hDF9
hDF10
b
High quality data (Gb)
Mapping ratio (% against the reference genome)
RNA-seq
hESCs (n = 4)
hDFs (n = 4)
High quality data (Gb)
Mapping ratio (%)
Naive hESC-1
Naive hESC-2
Primed hESC-1
Primed hESC-2
hDF-1
hDF-2
hDF-3
hDF-4
c
Percentage (%)
Resolution of hESCs
Resolution of hDFs
d
(hESCs)
3 libraries
Total ~0.76 billion contacts
(~252.62 M contacts per library)
(hDFs)
10 libraries
Total ~1.80 billion contacts
(~179.62 M contacts per library)
hESCs
hDFs
Stratum adjusted correlation coefficient
e
hESCs
hDFs
Intermingle index
f
Von Neumann entropy
g
GC ratio
P = 0.1
P = 1.05×10-3
PCGs number/Mb
P = 1.08×10-5
Median TPM
h
AA×BB/(AB²)
i
Intra (A-A)
Intra (B-B)
Inter (A-B)
j
P = 3.07 ×10-7
P < 2.20 ×10-16
Log2 TPM (hESCs/hDFs)
n = 13,799
n = 741
n = 462
Stable
A to B
B to A
hESCs to hDFs
k
A in hESCs to B in hDFs
Homophilic cell adhesion via plasma membrane adhesion molecules (51/170)
Detection of chemical stimulus (81/511)
Detection of chemical stimulus involved in sensory perception of bitter taste (21/41)
Negative regulation of proteolysis (45/375)
Monovalent inorganic cation transport (49/551)
Synaptic signaling (56/741)
Butanoate metabolism (7/28)
Copulation (6/20)
Retinoic acid biosynthetic process (4/8)
Glutamate catabolic process (4/8)
Drug transport (21/231)
Salivary secretion (12/94)
Signal release (34/476)
Response to jasmonic acid (3/4)
Positive regulation of thyroid hormone generation (3/4)
Negative regulation of flagellated sperm motility (3/4)
Sodium-independent organic anion transport (5/16)
Positive regulation of nitric-oxide synthase biosynthetic process (5/16)
Cellular response to calcium ion (11/83)
Defense response to Gram-negative bacterium (11/84)
-log10 (P-value)
B in hESCs to A in hDFs
Extracellular matrix organization (36/397)
Focal adhesion (21/215)
Defense response to Gram-positive bacterium (14/102)
Wound healing (34/593)
Proteoglycans in cancer (19/218)
Positive regulation of locomotion (31/621)
Transmembrane receptor protein tyrosine kinase signaling pathway (35/753)
Receptor internalization (12/116)
Positive regulation of MAPK cascade (28/556)
Chemotaxis (30/656)
Regulated exocytosis (34/798)
Cellular response to nitrogen compound (30/666)
Endodermal cell differentiation (7/46)
ECM-receptor interaction (9/82)
Supramolecular fiber organization (31/719)
Regulation of muscle system process (16/256)
Circulatory system process (26/559)
Transcriptional misregulation in cancer (13/180)
Peptidyl-tyrosine phosphorylation (20/375)
Eye morphogenesis (12/156)
-log10 (P-value)
l
Strength of intra-TAD contacts
m
Average DI
Boundary
-500 kb
+500 kb
Average IS

Data S1: Additional Hi-C Maps, rRNA-depleted RNA-seq and ChIP-seq (H3K27ac), Relevant to Main Findings, Related to Fig. 5

Data S1-II: Hi-C Maps and RNA-seq of hESCs and hDFs

a Data summary of publicly available Hi-C data for hESCs (three libraries; a total of ~757.87 M valid contacts with a depth of ~252.62 M contacts per library) [3,4] and hDFs (ten libraries; a total of ~1.80-billion valid contacts with a depth of ~179.62 M contacts per library) [5]. The intra-chromosomal read pairs (~137.54 M contacts, or 69.68% of valid contacts per library) are the dominant interactions compared to inter-chromosomal read pairs. **b** Data summary of human RNA-seq data. **c** Evaluation of Hi-C resolution. Individual replicates achieved resolutions of 6 to 16 kb and 8 to 33 kb for hESCs and hDFs, respectively. **d** Estimated interrelationships between 13 normalized (as implemented in KR algorithm [6] and quantile algorithm [7]) intra-chromosomal Hi-C maps at 100-kb resolution of hESCs (three green triangles) and hDFs (ten red circles) using the HiCRep tool [2]. The Hi-C maps are highly reproducible between replicates of hESCs (median SCC = 0.87) and hDFs (SCC = 0.92), but are more dissimilar between hESCs and hDFs (SCC = 0.70). **e** Probability of extensive multi-chromosome intermingling (average of Hi-C maps for each cell type) across 22 autosomes based on intra- (at 100-kb resolution) and inter-chromosomal (at 1-Mb resolution) contact maps in hESCs (green) and hDFs (red). **f** The extent of disorder in chromatin structure (quantified by the VNE) at 100-kb resolution in each Hi-C map of hESCs (green) and hDFs (red). Compared to hDFs, the chromatin of hESCs was more permissive and disordered (reflected by the high-entropy statue in hESCs compared to hDFs: 2.03/1.20, $P = 7.35 \times 10^{-4}$, Wilcoxon rank-sum test). **g** Compared to compartment B regions (43.81% of genome in hESCs and 51.86% of genome in hDFs), the compartment A regions (56.19% of genome in hESCs and 48.14% of genome in hDFs) are more GC-rich, gene-rich, and had relatively higher transcriptional activities. **h** Compartmentalization strength (AA × BB/AB2) across 22 autosomes at 20-kb resolution in each Hi-C map of hESCs (green) and hDFs (red). Note that strength was substantially lost in hESCs compared to hDFs (1.70/3.17, $P = 9.22 \times 10^{-11}$, Wilcoxon rank-sum test). **i** Comparison of compartment contacts across 22 autosomes at 20-kb resolution in each Hi-C map. The dramatic decrease of compartmentalization strength ($AA \times BB/AB^2$) in hESCs compared to hDFs (Supplementary information, Data S1, IIh) is not only attributed to the increased inter-compartment contacts in hESCs (hESCs compared to hDFs: 0.79/0.71, $P = 1.84 \times 10^{-3}$, Wilcoxon rank-sum test), but also resulted in more reduced contacts within the B compartment (hESCs compared to hDFs: 0.82/1.22, $P = 9.94 \times 10^{-5}$, Wilcoxon rank-sum test) and slightly increased contacts within the A compartment (hESCs compared to hDFs: 1.41/1.37, $P = 0.52$, Wilcoxon rank-sum test) in hESCs. **j** Expression changes of genes located in regions exhibiting compartments A/B switching between hESCs and hDFs. Genes detected in at least one cell type are shown. **k** Functional enrichment for 1 072 genes from A status in hESCs to B status in hDFs (yellow bars), and 559 genes from B status in hESCs to A status in hDFs (green bars). The top functional terms of Metascape (https://metascape.org) [8] summary gene set in each enriched cluster are shown, with the constraint of showing no more than 20 terms. The number after each term represents the hit genes out of total genes in this term. **l** The extent of the strength of intra-TAD contacts in hESCs and hDFs. Data represented as violin plots based on the strength of intra-TAD contacts for each TAD. **m** Average directionality index (DI) (left) [9] and insulation score (IS) (right) [10] in a 1 Mb region centered on shared TAD boundaries between hESCs and hDFs. For (E), (F), (G), (H), (I), (J), and (L), statistical significance was calculated by Wilcoxon rank-sum test (n.s., $P \geq 0.05$; *, $P < 0.05$; **, $P < 0.01$, ***, $P < 0.001$).

DATA S1-III

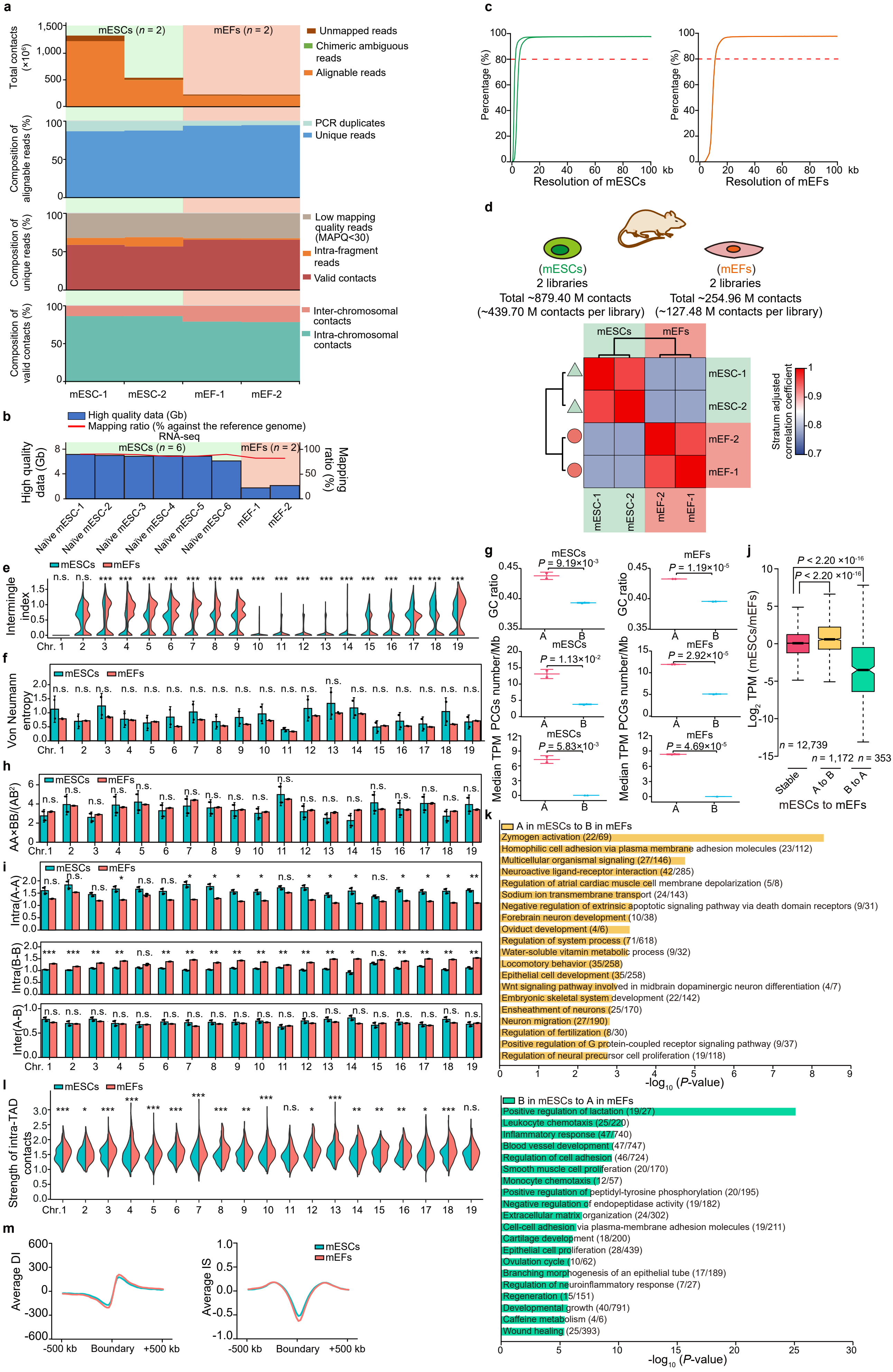

a
mESCs (n = 2)
mEFs (n = 2)
Total contacts (×10^6)
Unmapped reads
Chimeric ambiguous reads
Alignable reads
Composition of alignable reads (%)
PCR duplicates
Unique reads
Composition of unique reads (%)
Low mapping quality reads (MAPQ<30)
Intra-fragment reads
Valid contacts
Composition of valid contacts (%)
Inter-chromosomal contacts
Intra-chromosomal contacts
mESC-1
mESC-2
mEF-1
mEF-2
b
High quality data (Gb)
Mapping ratio (% against the reference genome)
RNA-seq
mESCs (n = 6)
mEFs (n = 2)
High quality data (Gb)
Mapping ratio (%)
Naive mESC-1
Naive mESC-2
Naive mESC-3
Naive mESC-4
Naive mESC-5
Naive mESC-6
mEF-1
mEF-2
c
Percentage (%)
Resolution of mESCs
Resolution of mEFs
d
(mESCs)
2 libraries
Total ~879.40 M contacts
(~439.70 M contacts per library)
(mEFs)
2 libraries
Total ~254.96 M contacts
(~127.48 M contacts per library)
Stratum adjusted correlation coefficient
e
mESCs
mEFs
Intermingle index
Chr. 1
f
Von Neumann entropy
g
GC ratio
P = 9.19×10^-3
P = 1.19×10^-5
Median TPM PCGs number/Mb
P = 1.13×10^-2
P = 2.92×10^-5
Median TPM
P = 5.83×10^-3
P = 4.69×10^-5
h
AA×BB/(AB^2)
i
Intra(A-A)
Intra(B-B)
Inter(A-B)
j
P < 2.20 ×10^-16
Log2 TPM (mESCs/mEFs)
n = 12,739
n = 1,172
n = 353
Stable
A to B
B to A
mESCs to mEFs
k
A in mESCs to B in mEFs
Zymogen activation (22/69)
Homophilic cell adhesion via plasma membrane adhesion molecules (23/112)
Multicellular organismal signaling (27/146)
Neuroactive ligand-receptor interaction (42/285)
Regulation of atrial cardiac muscle cell membrane depolarization (5/8)
Sodium ion transmembrane transport (24/143)
Negative regulation of extrinsic apoptotic signaling pathway via death domain receptors (9/31)
Forebrain neuron development (10/38)
Oviduct development (4/6)
Regulation of system process (71/618)
Water-soluble vitamin metabolic process (9/32)
Locomotory behavior (35/258)
Epithelial cell development (35/258)
Wnt signaling pathway involved in midbrain dopaminergic neuron differentiation (4/7)
Embryonic skeletal system development (22/142)
Ensheathment of neurons (25/170)
Neuron migration (27/190)
Regulation of fertilization (8/30)
Positive regulation of G protein-coupled receptor signaling pathway (9/37)
Regulation of neural precursor cell proliferation (19/118)
-log10 (P-value)
B in mESCs to A in mEFs
Positive regulation of lactation (19/27)
Leukocyte chemotaxis (25/220)
Inflammatory response (47/740)
Blood vessel development (47/747)
Regulation of cell adhesion (46/724)
Smooth muscle cell proliferation (20/170)
Monocyte chemotaxis (12/57)
Positive regulation of peptidyl-tyrosine phosphorylation (20/195)
Negative regulation of endopeptidase activity (19/182)
Extracellular matrix organization (24/302)
Cell-cell adhesion via plasma-membrane adhesion molecules (19/211)
Cartilage development (18/200)
Epithelial cell proliferation (28/439)
Ovulation cycle (10/62)
Branching morphogenesis of an epithelial tube (17/189)
Regulation of neuroinflammatory response (7/27)
Regeneration (15/151)
Developmental growth (40/791)
Caffeine metabolism (4/6)
Wound healing (25/393)
-log10 (P-value)
l
Strength of intra-TAD contacts
m
Average DI
Average IS
-500 kb
Boundary
+500 kb

Data S1: Additional Hi-C Maps, rRNA-depleted RNA-seq and ChIP-seq (H3K27ac), Relevant to Main Findings, Related to Fig. 5

Data S1-III: Hi-C Maps and RNA-seq of mESCs and mEFs

a Data summary of publicly available Hi-C data for mESCs (two libraries; a total of ~ 879.40 M valid contacts with a depth of ~ 439.70 M contacts per library) [11] and mEFs (two libraries; a total of ~254.96 M valid contacts with a depth of ~127.48 M contacts per library) [12]. The intra-chromosomal read pairs (~240.06 M contacts, or 82.59% of valid contacts per library) are the dominant interactions compared to inter-chromosomal read pairs. **b** Data summary of mouse RNA-seq data. **c** Evaluation of Hi-C resolution. Individual replicates achieved resolutions of 3 to 6 kb and 12 kb for mESCs and mEFs, respectively. **d** Estimated interrelationships between four normalized (as implemented in KR algorithm [6] and quantile algorithm [7]) intra-chromosomal Hi-C maps at 100-kb resolution of mESCs (two green triangles) and mEFs (two red circles) using the HiCRep tool [2]. The Hi-C maps are highly reproducible between replicates of mESCs (SCC = 0.96) and mEFs (SCC = 0.96), but are more dissimilar between mESCs and mEFs (median SCC = 0.78). **e** Probability of extensive multi-chromosome intermingling (average of Hi-C maps for each cell type) across 19 autosomes based on intra- (at 100-kb resolution) and inter-chromosomal (at 1-Mb resolution) contact maps in mESCs (green) and mEFs (red). Note the overall extent of chromosome intermingling is less in the mESCs than in mEFs (0.32/0.40, $P < 2.2 \times 10^{-16}$, Wilcoxon rank sum test). Statistical significance was calculated by Wilcoxon rank-sum test (n.s., P ≥ 0.05; *, $P < 0.05$; **, $P < 0.01$, ***, $P < 0.001$). **f** The extent of disorder in chromatin structure (quantified by the VNE) at 100-Kb resolution in each Hi-C map of mESCs (green) and mEFs (red), respectively. Compared to mEFs, the chromatin of mESCs was more permissive and disordered (reflected by the high-entropy statue in mESCs compared to mEFs: 0.86/0.68, $P = 4.30 \times 10^{-2}$, Wilcoxon rank-sum test). **g** Compared to compartment B regions (49.26% of genome in mESCs and 50.74% of genome in mEFs), the compartment A regions (50.74% of genome in mESCs and 49.26% of genome in mEFs) are more GC-rich, gene-rich and have relatively higher transcriptional activities. **h** Compartmentalization strength [$AA \times BB/AB^2$] across 19 autosomes at 20-kb resolution in each Hi-C map of mESCs (green) and mEFs (red). The compartmentalization was subtly decreased in mESCs compared to mEFs (3.46/3.55, $P = 0.75$, Wilcoxon rank-sum test). **i** Comparison of compartment contacts across 19 autosomes at 20-kb resolution in each Hi-C map. **j** Expression changes of genes located in regions exhibiting compartment switching between mESCs and mEFs. Genes detected in at least one cell type are shown. **k** Functional enrichment for 1 901 genes from A status in mESCs to B status in mEFs (yellow bars), and 572 genes from B status in mESCs to A status in mEFs (green bars). The top functional terms of Metascape (https://metascape.org) [8] summary gene set in each enriched cluster are shown, with the constraint that no more than 20 terms are portrayed. The number after each term represents the hit genes out of total genes in this term. **l** The extent of TAD intactness in mESCs and mEFs. Data represented as violin plots based on the TAD intactness per TAD. Note the TAD intactness of mESCs was weaker than that in mEFs. **m** Average directionality index (DI) (left) [9] and insulation score (IS) [10] (right) in a 1 Mb region centered on shared TAD boundaries between mESCs and mEFs.

For (**e**), (**g**), (**j**), and (**l**), statistical significance was calculated by Wilcoxon rank-sum test, (**f**), (**h**), and (**i**) was calculated by Student's *t*-test (n.s., $P \geq 0.05$; *, $P < 0.05$; **, $P < 0.01$, ***, $P < 0.001$).

Reference:

1 Rao, S. S. *et al.* A 3D map of the human genome at kilobase resolution reveals principles of chromatin looping. *Cell* **159**, 1665-1680 (2014).

2 Yang, T. *et al.* HiCRep: assessing the reproducibility of Hi-C data using a stratum-adjusted correlation coefficient. *Genome Res.* **27**, 1939-1949 (2017).

3 Dixon, J. R. *et al.* Chromatin architecture reorganization during stem cell differentiation. *Nature* **518**, 331-336 (2015).

4 Lyu, X., Rowley, M. J. & Corces, V. G. Architectural Proteins and Pluripotency Factors Cooperate to Orchestrate the Transcriptional Response of hESCs to Temperature Stress. *Mol. Cell* **71**, 940-955 e947 (2018).

5 Nir, G. *et al.* Walking along chromosomes with super-resolution imaging, contact maps, and integrative modeling. *PLoS Genet.* **14**, e1007872 (2018).

6 Durand, N. C. *et al.* Juicer Provides a One-Click System for Analyzing Loop-Resolution Hi-C Experiments. *Cell Syst.* **3**, 95-98 (2016).

7 Fletez-Brant, K., Qiu, Y., Gorkin, D. U., Hu, M. & Hansen, K. D. Removing unwanted variation between samples in Hi-C experiments. *bioRxiv*, 214361 (2021).

8 Zhou, Y. *et al.* Metascape provides a biologist-oriented resource for the analysis of systems-level datasets. *Nat. Commun.* **10**, 1523 (2019).

9 Dixon, J. R. *et al.* Topological domains in mammalian genomes identified by analysis of chromatin interactions. *Nature* **485**, 376-380 (2012).

10 Crane, E. *et al.* Condensin-driven remodelling of X chromosome topology during dosage compensation. *Nature* **523**, 240-244 (2015).

11 Bonev, B. *et al.* Multiscale 3D Genome Rewiring during Mouse Neural Development. *Cell* **171**, 557-572 e524 (2017).

12 Di Giammartino, D. C. *et al.* KLF4 is involved in the organization and regulation of pluripotency-associated three-dimensional enhancer networks. *Nat. Cell Biol.* **21**, 1179-1190 (2019).
