# Supplementary material for: Generation and characterization of stable pig pregastrulation epiblast stem cell lines
Source: Cell Res. 2021 Nov 30;32(4):383–400. doi: 10.1038/s41422-021-00592-9 (PMC8976023; doi:10.1038/s41422-021-00592-9)
Supplement: Supplementary file 12 — Supplementary information, Data S2 [file 41422_2021_592_MOESM12_ESM.pdf]

DATA S2-I

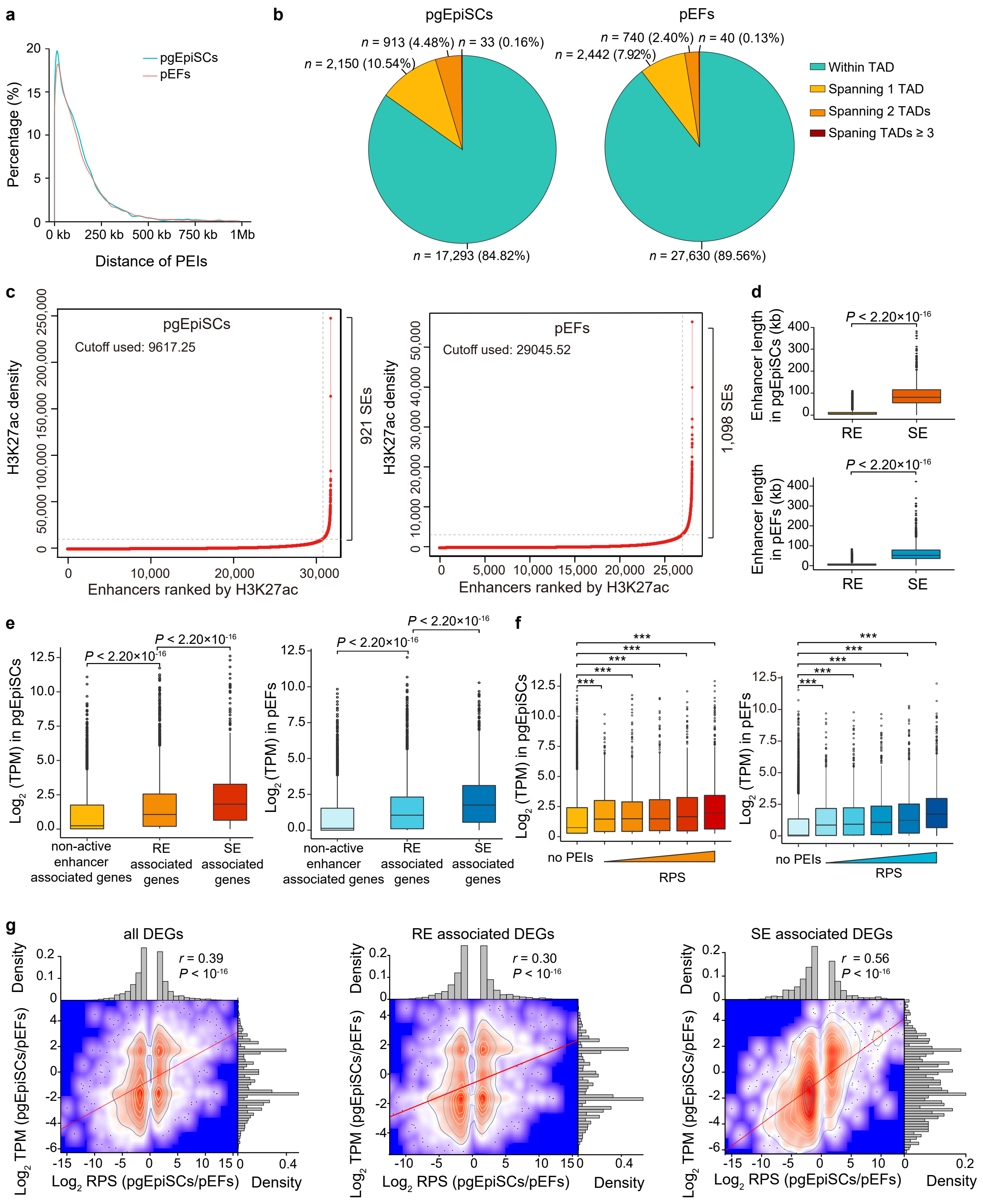

a
pgEpiSCs
pEFs
Percentage (%)
Distance of PEIs
0 kb 250 kb 500 kb 750 kb 1Mb
b
pgEpiSCs
pEFs
n = 913 (4.48%) n = 33 (0.16%)
n = 2,150 (10.54%)
n = 17,293 (84.82%)
n = 740 (2.40%) n = 40 (0.13%)
n = 2,442 (7.92%)
n = 27,630 (89.56%)
Within TAD
Spanning 1 TAD
Spanning 2 TADs
Spaning TADs ≥ 3
c
pgEpiSCs
Cutoff used: 9617.25
H3K27ac density
921 SEs
Enhancers ranked by H3K27ac
pEFs
Cutoff used: 29045.52
1,098 SEs
Enhancers ranked by H3K27ac
d
P < 2.20×10-16
Enhancer length in pgEpiSCs (kb)
Enhancer length in pEFs (kb)
RE
SE
e
P < 2.20×10-16
Log2 (TPM) in pgEpiSCs
Log2 (TPM) in pEFs
non-active enhancer associated genes
RE associated genes
SE associated genes
f
***
Log2 (TPM) in pgEpiSCs
Log2 (TPM) in pEFs
no PEIs
RPS
g
all DEGs
r = 0.39
P < 10-16
RE associated DEGs
r = 0.30
P < 10-16
SE associated DEGs
r = 0.56
P < 10-16
Density
Log2 TPM (pgEpiSCs/pEFs)
Log2 RPS (pgEpiSCs/pEFs)

Data S2: Additional Results of Promoter-enhancer Interactions (PEIs), Relevant to Main Findings, Related to Fig. 6

Data S2-I: Additional Results of Promoter-enhancer Interactions (PEIs)

a Percentage of chromatin interactions that span a given genomic distance. More than a half of PEIs (54.79% in pgEpiSCs and 54.77% in pEFs) span 100 kb or larger genomic distance (with a median distance of 110 kb for pgEpiSCs and pEFs). **b** Percentages of PEIs within TADs or spanning TADs. Consistent with the finding of stronger spatial segregation (thus more self-interaction) in pEFs compared to pgEpiSCs (Fig. 5), we observed PEIs were constrained primarily inside TADs and are more evident in pEFs (89.56% PEIs are occurred within TADs) compared to pgEpiSCs (84.82%). **c** Saturation curves of H3K27ac density. The number of ranked regular enhancers (REs) and super enhancers (SEs) by H3K27ac density (x-axis) and their densities (y-axis) are plotted. Horizontal dotted lines represent density cutoffs used for the classification of SEs and vertical dotted lines demarcate SEs from REs. The total number of predicted SEs is noted on the right side of each graph. **d** The distribution of length of REs and SEs. About one fifth of promoter-interacting enhancers are classified as SEs (4 005 of 20 389, or 19.64% in pgEpiSCs; 6 831 of 30 852, or 22.14% in pEFs), which exhibited larger domain size and higher levels of H3K27ac signal than REs (16 384 of 20 389, or 80.36% in pgEpiSCs; 24 021 of 30 852, or 77.86% in pEFs). **e** Comparison of expression between non-enhancer/RE/SE associated genes. As expected, the promoters contacting SEs (median TPM = 2.52 in pgEpiSCs, 2.37 in pEFs) show higher expression than promoters contacting REs (median TPM = 1.03 in pgEpiSCs, 1.07 in pEFs) and promoters depleted with enhancers (median TPM = 0.18 in pgEpiSCs, 0.09 in pEFs). **f** Comparison of gene expression separated by RPS categories. **g** Correlation between differential RPS and fold-changes of differentially expressed genes (DEGs). As expected, the enhancer-interacting genes with larger RPS were more highly expressed (Pearson's $r = 0.39$, $P < 10^{-16}$), especially for SE-interacting genes (Pearson's $r = 0.56$ compared to $r = 0.30$ for RE-interacting genes $P < 10^{-16}$). This result supports the notion that regulatory information to direct transcription is conveyed through the physical PEIs, and demonstrates that the majority of the enhancers identified had a measurable additive effect on target-gene transcription [1-3].
For (**d**), (**e**) and (**f**), statistical significance was calculated by Wilcoxon rank-sum test (n.s., $P \geq 0.05$; *, $P < 0.05$; **, $P < 0.01$, ***, $P < 0.001$).

DATA S2-II

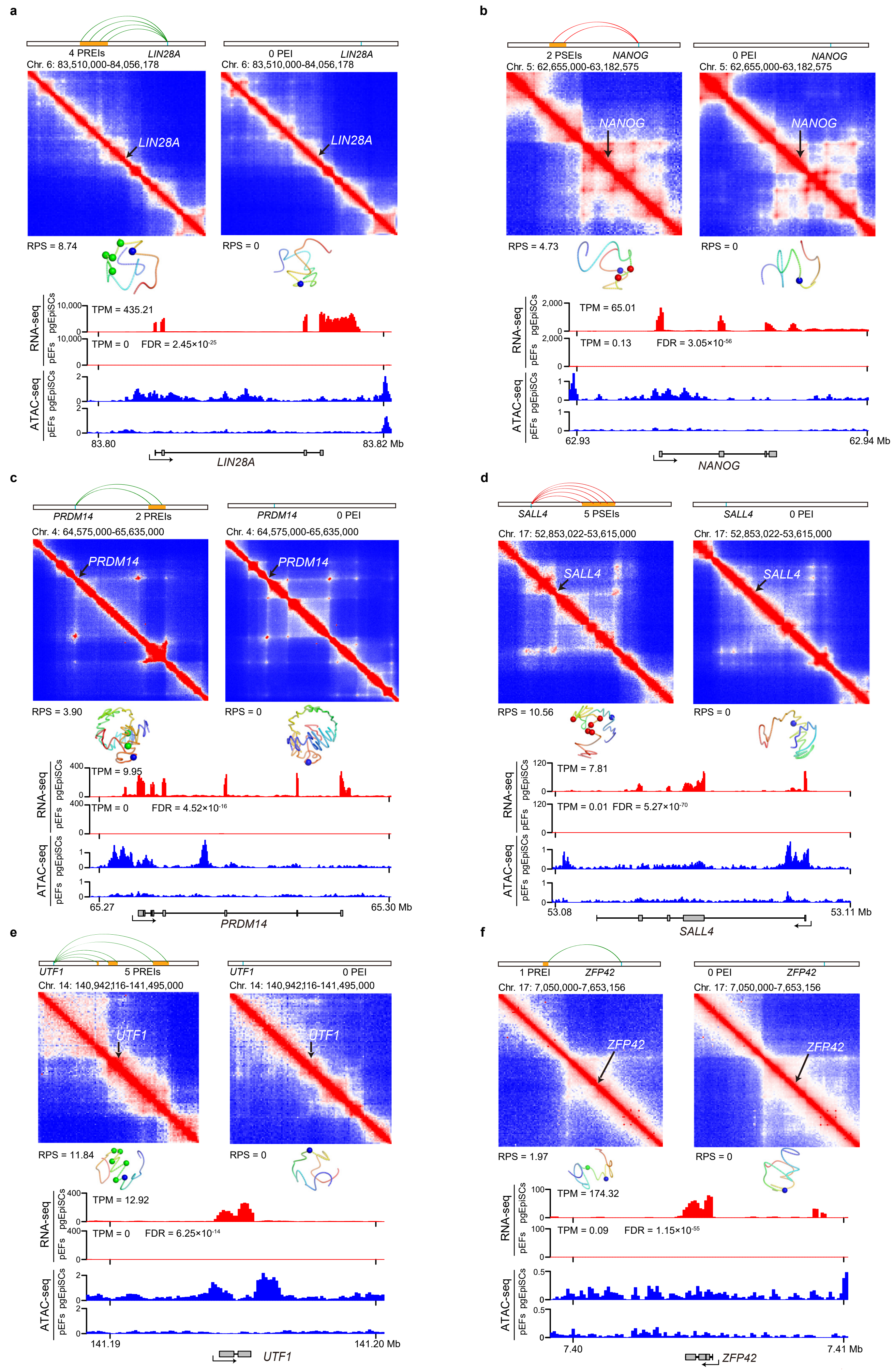
a
4 PREIs
LIN28A
Chr. 6: 83,510,000-84,056,178
0 PEI
RPS = 8.74
RPS = 0
RNA-seq
ATAC-seq
pgEpiSCs
pEFs
TPM = 435.21
TPM = 0
FDR = 2.45×10^-25
83.80
83.82 Mb
b
2 PSEIs
NANOG
Chr. 5: 62,655,000-63,182,575
RPS = 4.73
TPM = 65.01
TPM = 0.13
FDR = 3.05×10^-56
62.93
62.94 Mb
c
PRDM14
2 PREIs
Chr. 4: 64,575,000-65,635,000
RPS = 3.90
TPM = 9.95
FDR = 4.52×10^-16
65.27
65.30 Mb
d
SALL4
5 PSEIs
Chr. 17: 52,853,022-53,615,000
RPS = 10.56
TPM = 7.81
TPM = 0.01
FDR = 5.27×10^-70
53.08
53.11 Mb
e
UTF1
5 PREIs
Chr. 14: 140,942,116-141,495,000
RPS = 11.84
TPM = 12.92
FDR = 6.25×10^-14
141.19
141.20 Mb
f
1 PREI
ZFP42
Chr. 17: 7,050,000-7,653,156
RPS = 1.97
TPM = 174.32
TPM = 0.09
FDR = 1.15×10^-55
7.40
7.41 Mb

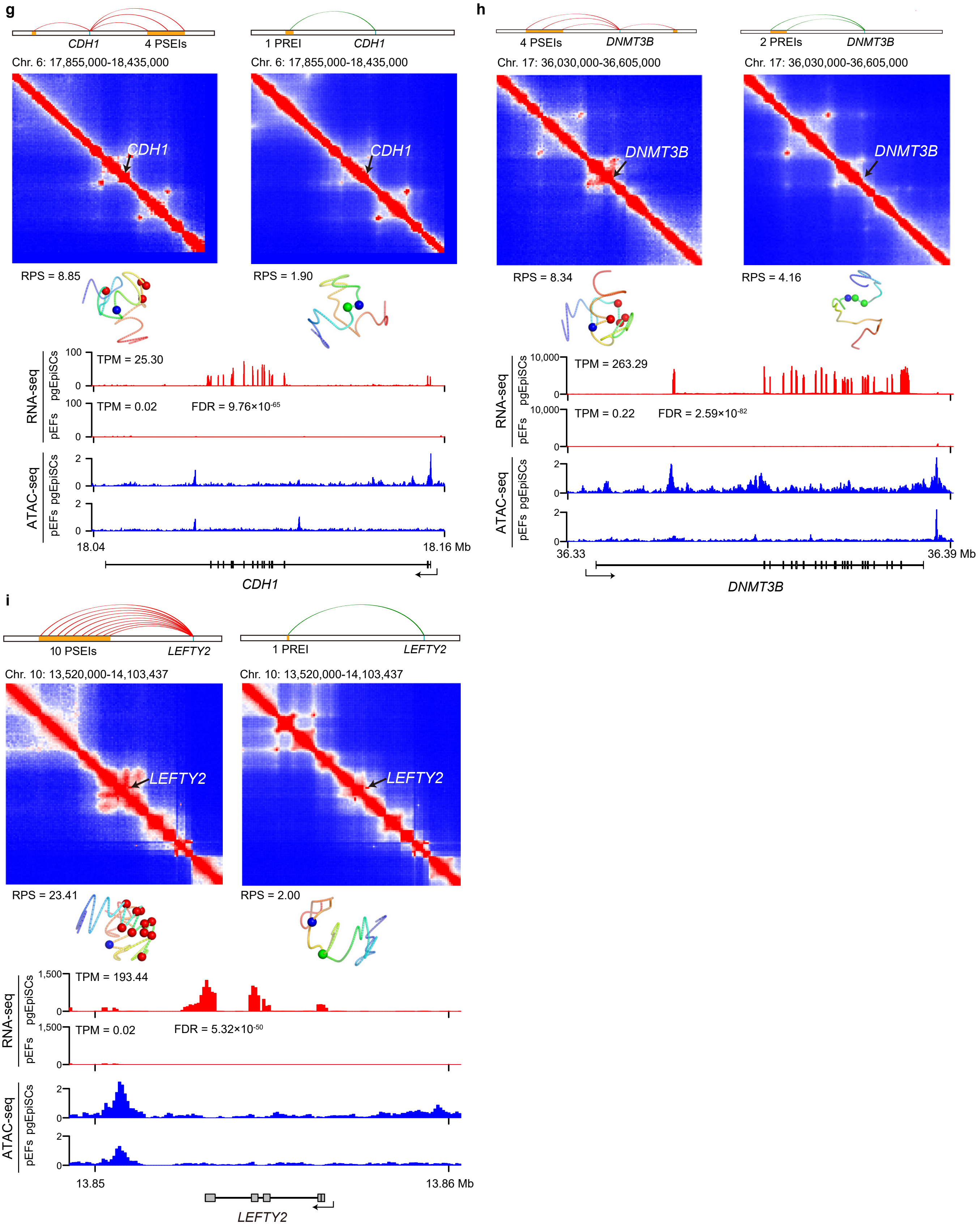
g
CDH1
4 PSEIs
1 PREI
CDH1
Chr. 6: 17,855,000-18,435,000
Chr. 6: 17,855,000-18,435,000
CDH1
CDH1
RPS = 8.85
RPS = 1.90
RNA-seq
pgEpiSCs
pEFs
100
0
TPM = 25.30
TPM = 0.02
FDR = 9.76×10-65
ATAC-seq
2
0
18.04
18.16 Mb
CDH1
h
4 PSEIs
DNMT3B
2 PREIs
DNMT3B
Chr. 17: 36,030,000-36,605,000
Chr. 17: 36,030,000-36,605,000
DNMT3B
DNMT3B
RPS = 8.34
RPS = 4.16
10,000
TPM = 263.29
TPM = 0.22
FDR = 2.59×10-82
36.33
36.39 Mb
DNMT3B
i
10 PSEIs
LEFTY2
1 PREI
LEFTY2
Chr. 10: 13,520,000-14,103,437
Chr. 10: 13,520,000-14,103,437
LEFTY2
LEFTY2
RPS = 23.41
RPS = 2.00
1,500
TPM = 193.44
TPM = 0.02
FDR = 5.32×10-50
13.85
13.86 Mb
LEFTY2

Data S2: Additional Results of Promoter-enhancer Interactions (PEIs), Relevant to Main Findings, Related to Fig. 6

Data S2-II: Additional Results of Schematic Representation of the PEIs for Genes That Are Highly Abundant in pgEpiSCs (TPM > 5 compared to TPM < 0.5 in pEFs)

The green, red and blue balls represent RE, SE and promoter, respectively. The Benjamini-Hochberg adjusted FDRs were calculated by DEseq2 tool (version 1.28.1) for estimation of statistical significance.

Reference:

1 Cao, Q. *et al.* Reconstruction of enhancer-target networks in 935 samples of human primary cells, tissues and cell lines. *Nat. Genet.* **49**, 1428-1436 (2017).

2 Fulco, C. P. *et al.* Activity-by-contact model of enhancer-promoter regulation from thousands of CRISPR perturbations. *Nat. Genet.* **51**, 1664-1669 (2019).

3 Whalen, S., Truty, R. M. & Pollard, K. S. Enhancer-promoter interactions are encoded by complex genomic signatures on looping chromatin. *Nat. Genet.* **48**, 488-496 (2016).
