# Supplementary material for: Generation and characterization of stable pig pregastrulation epiblast stem cell lines
Source: Cell Res. 2021 Nov 30;32(4):383–400. doi: 10.1038/s41422-021-00592-9 (PMC8976023; doi:10.1038/s41422-021-00592-9)
Supplement: Supplementary file 13 — Supplementary information, Data S3 [file 41422_2021_592_MOESM13_ESM.pdf]

Supplementary information, Data S3, related to Materials and Methods

Single-cell RNA-seq Processing

Raw reads from scRNA-seq were split by 8 bp cell barcodes located on Read 2 allowing 2 mismatches. Additionally, the 8 bp unique molecular identifiers (UMIs) located on Read 2 were switched to the identifier line of paired Read 1 [1,2]. Then Read 1 was processed to remove the template switch oligo (TSO) primer, low quality bases and polyA sequence. The trimmed reads were aligned against the Ensembl *Sus scrofa* reference genome (Sscrofa11.1, GCA_000003025.6) using STAR software (version 2.7.1a) with default parameters [3]. The aligned reads were further assigned to the Ensembl gene annotations (Sus_scrofa.Sscrofa11.1.98) using featureCounts (version 1.6.4) [4]. Gene expression levels were estimated by counting the UMIs assigned to the genes after deduplication by UMI-tools [5], yielding an expression matrix consisting of UMI counts for each cell and gene.

For downstream analysis, the expression matrix was filtered based on the following criteria to get high quality cells and detectable genes: 1) the number of genes and UMIs should be more than 2 000 and 5 000 for each cell, respectively; 2) the proportions of UMIs for external RNA controls consortium (ERCC) and mitochondrial genes should be below 20%; and 3) maximum cell-to-cell Pearson's correlations for each cell should be above 0.6 to exclude outlier cells. Moreover, genes should be detected in at least 5 out of 2 058 sequenced cells, and the genes located on the sex chromosomes were excluded to eliminate gender interference as sequenced cells were derived from different embryos of both genders. After filtering, 1 908 (1 717 embryonic cells, 189 pgEpiSC cells) out of 2 058 cells were retained in the high-quality expression matrix for down-stream clustering and cell type identification (Supplementary information, Table S1). The expression level for each gene was transformed to $\log_2(TPM/10 + 1)$, and TPM values were divided by 10 to account for low complexity of single-cell libraries [6].

Clustering and Cell Type Identification

The filtered expression matrix was loaded into R package Seurat (verison 3.0.0) [7] to cluster and characterize the cell types in porcine preimplantation embryos. Raw UMI counts were normalized and scaled after regression on the total UMI number, mitochondrial gene ratio and expressed gene number per cell using the SCTransform function. Additionally, porcine homologs for predefined human gene sets specifically expressed in S and G2/M cell cycle phases were used to calculate cell cycle score via the CellCycleScoring function, and the cell cycle scores were integrated with the aforementioned features to recalibrate the expression data by rerunning the SCTransform function. Recalibrated data was then used for principal components analysis (PCA) using RunPCA on the top 3 000 highly variable genes with default parameters. Dimensionality reduction was applied to the first ten principal components, and the clusters were identified by construction of a shared nearest neighbor (SNN) graph. Finally, we visualized cell clusters after projecting them into two-dimensional latent space via t-distributed stochastic neighbor embedding (t-SNE).

We collected the known marker genes expressed in embryonic lineages [8], and ranked these genes in each cell based on expression levels and calculated the area under curve (AUC) to estimate gene signature scores for each cell using the AUCell_calcAUC function in AUCell package [9]. According to the gene signature scores, we annotated the clusters at each embryonic day to known lineages. The cells derived from embryos at E5 were divided into pre-ICM and pre-TE, and the E6 cells were assigned to ICM and TE. Sequentially, cells from E7 to E10 were additionally subdivided into epiblast, hypoblast and TE, cells from E11 to E14 were further classified into ectoderm, mesoderm, endoderm (definitive endoderm since E12) and TE (Fig. 1b). The cell type annotation was further supported by visualizing the expression level of marker genes and enriched functional

terms (Supplementary information, Fig. S1d; Table S2). In addition, to explore the transcriptome characteristics of early pig embryonic development, 259 out of 1 717 embryonic cells were excluded, due to the ambiguous classification of embryonic linages. Downstream analysis and demonstration were conducted on these 1 458 cells (Supplementary information, Table S1).

Identification of Differentially Expressed Genes between Embryonic Stages in Respective Lineages

Based on the differentiation process during embryonic development, we grouped the porcine embryonic cells into three main lineages after the morula stage: embryonic lineages, covering morula at E4, pre-ICM at E5, ICM at E6, epiblasts ranging from E7 to E10, and the ectoderms from E11 to E14; TE lineages, including pre-TE at E5 and TE ranging from E6 to E14; and hypoblast lineages, consisting of hypoblasts ranging from E7 to E10, endoderm at E11-E12, and definitive endoderm at E13-E14 (Fig. 1b).

We then detected DEGs between all the embryonic development time points in each respective lineage. This was done to identify the genes critical for the differentiation process, especially for embryonic lineages, which were used to design the culture system of the pgEpiSC cells. Differential expression analysis was performed using the FindMarkers function in the Seurat package based on the Wilcoxon rank-sum test, and genes detected in at least 20% of the two compared cell populations were considered. The DEGs with absolute average log transformed fold change >0.5 and adjusted P value < 0.05 were retained for downstream functional enrichment analysis (Supplementary information, Table S3).

Construction of Expression Tendencies

To trace the dynamic changes of DEGs during embryonic development, we constructed the expression tendencies of DEGs in epiblast lineages. We first calculated the average expression levels for each gene at specific embryonic development time points in each respective lineage separately. The average expression levels were rescaled for each lineage and analyzed by the k-means clustering method with parameters k=36 and iter.max=100, grouping the DEGs with similar tendencies during embryonic development into separate clusters (Supplementary information, Table S3). The average and standard deviation of scaled expression levels for each cluster were calculated to evaluate the performance of clustering.

Co-expression Network Analysis of Epiblast Lineage

To obtain the core co-expression network during embryonic development in the epiblast lineage, we constructed a signed weighted co-expression network using R package WGCNA (version 1.68) [10]. We first filtered the raw UMI matrix by selecting the top 50% most highly variable genes, and measured the pair-wise correlations between these genes. Next, co-expression modules were constructed by using 'blockwiseModules' based on the correlation matrix with parameter soft-threshold power set to 12. Modules with eigengene correlations above 0.8 were further merged. Genes with edge weight above 0.02, and shared with the DEGs in the epiblast lineage between different embryonic stages, were retained. The co-expressed network of selected genes was visualized by running circular layout as part of the Cytoscape core (Supplementary information, Fig. S2a) (version 3.8.0) [11].

Pseudotime Analysis of Pig Embryonic Cells

The development trajectory based on pseudotime was inferred by using Monocle3 [12]. The marker genes represented for each lineage (listed in Supplementary information, Table S2c) were used to order the cells in pseudotime analysis, the expression level matrix of these genes was loaded and created an object. After the dimension reduction, clustering and graph learning process by following Monocle3 tutorial, we constructed the pig embryonic development trajectory via pseudotime analysis.

RNA velocity analysis of Pig Embryonic Cells

For RNA velocity analysis, we first counted the spliced reads and unspliced reads via the bam files of scRNA-seq by using the velocyto run command [13], the counts of spliced reads and unspliced reads were used to calculated the velocity values for each gene following the velocyto python pipeline with default parameters. Then the velocity vector was embedded into two-dimensional latent space generated by tSNE based on RNA abundance to trace developmental process of pig embryo.

Transcriptome Relatedness Analysis of pgEpiSCs to Embryonic Cells

The UMI matrix of pgEpiSCs were analyzed integratively with those of pig embryonic cells, using the accordant procedure descript in **Clustering and Cell Type Identification**, and the clusters of pgEpiSCs were visualized in contrast to those of pig embryonic cells (Fig. 4a). Differential expression analysis was performed by comparing the pgEpiSCs with epiblasts ranging from E7 to E10, and ectoderm from E11 to E14, and functional enrichment of the DEGs were performed to evaluate the differentiation and pluripotent characteristics of pgEpiSCs, then 102 DEGs over-represented in “Signaling pathways regulating pluripotency of stem cells” and “epithelial cell differentiation” were chosen to conduct PCA and correlation analyses using ‘prcomp’ and ‘cor’ functions in R (Supplementary information, Table S4a).

rRNA-depleted RNA-seq Processing and Analysis

High quality reads of six RNA-seq libraries were mapped to the reference pig genome (Sscrofa 11.1, GCA_000003025.6) using the alignment tool STAR (version 2.6.0c) [3] with default parameters, generating an average of ~50.06 million aligned reads (~79.51%) for each library. Expression levels of protein coding genes (gene annotation file [GTF] from Ensembl Sscrofa 11.1) were quantified as transcripts per million (TPM) using the high-speed transcript quantification tool Kallisto (version 0.44.0) [14]. We considered a gene as detected if its TPM value was more than 0.5 in at least two of four biological replicates for pgEpiSCs, or one of two biological replicates for pEFs. DEGs between pgEpiSCs and pEFs were identified using the DEseq2 tool (version 1.28.1) [15]. We used Benjamini-Hochberg adjusted false discovery rate (FDR) < 0.05 and absolute $\log_2$ (fold change) >1 as cut-offs for statistical significance.

ATAC-seq Processing and Analysis

Using trim-galore (version 0.6.4_dev, https://www.bioinformaticsbabrahamacuk/projects/trim_galore/), we first removed low quality bases and adaptors from the 150-bp paired-end ATAC-seq raw reads, with the options “-q 25 --phred33 --length 74 -e 0.1 --stringency 4 --paired”. The obtained high-quality reads were further aligned to the reference pig genome assembly utilizing Bowtie (version 2.3.5.1) [16], with “-t -q -N 1 -L 25 -X 2000 --no-mixed --no-discordant” parameters. Mitochondrial, low-quality (q < 10) and duplicated reads were filtered through SAMtools (version 0.1.19) [17], and the obtained BAM files were further converted to BED files using bedtools2 (version 2.27.1) [18]. Finally, we identified peaks utilizing MACS2 (version 2.1.1) [19] with options “--shift -100 --extsize 200 --nomodel -B --SPMR --format=BEDPE --keep-dup=1 --qvalue=0.05”.

We acquired the non-redundant union peak set by merging the peaks called from each separate sample with bedtools2 (version 2.27.1) [18]. We calculated the read pair numbers inside each non-redundant peak using HTseq (version 0.8.0) [20] `with options “--format=bam --order=pos --stranded=no --nonunique=all”. The differentially accessible regions were detected using EdgeR (version 3.20.9)[21] with fold-change > 4 and FDR < 10^{-5}. The over-represented motifs for each set of peaks were identified using the AME (Analysis of Motif Enrichment) tool packed within the MEME Suite (version 5.3.3) [22,23] (Supplementary information, Fig. S7).

ChIP-seq Processing and Peak Calling

High quality reads of four ChIP-seq libraries were aligned to the reference pig genome (Sscrofa 11.1) using the BWA tool (version 0.7.15) [24] with default parameters. The two biological replicates of each cell type were pooled using SAMtools (version 0.1.19) [17]. Peak calling was performed using SICER (version 1.1) [25]. To identify punctate enriched regions for H3K27ac marks, peak calling was performed with flags '--windowSize 200 --gapSize 3 --mapq 0 –effGenomeSize 0.95' along with a threshold of FDR < 0.05.

Hi-C Data Processing

Hi-C datasets were processed using the Juicer pipeline (version 1.8.9) [26]. Briefly, high-quality Hi-C reads were aligned against the reference pig genome (Sscrofa11.1) using BWA software (version 0.7.8) with default parameters. The aligning steps were followed by duplicated read removal provided in Juicer, and only unique reads were retained. Low-quality alignments (defined as MAPQ < 30) and intra-fragment reads were removed from unique reads, and valid Hi-C contacts were thus generated for subsequent analyses. Intra-chromosomal contract matrices were separately generated at different resolutions after normalization accounting for biases within a map (Knight and Ruiz algorithm [26]) and between maps (quantile algorithm [27]). The correlations between normalized intra-chromosomal matrices were calculated using the HiCRep tool [28] considering only intra-chromosomal contacts at a distance of less than 5-Mb. Inter-chromosomal contact matrices were generated after normalization accounting for biases within a map (Knight and Ruiz algorithm [26]) and between maps (counts per million [CPM] algorithm).

Chromatin 3D Modeling and Chromosome Intermingling

3D chromosome conformations were inferred for each Hi-C map based on normalized intra- (at 100-kb resolution) and inter-chromosomal (at 1-Mb resolution) contact maps using an approximation of the multidimensional scaling (MDS) method as implemented in the Python package miniMDS [29], and visualized using PyMOL software (version 2.4). The extent of chromosome intermingling for each 100-kb particle was estimated as previously described [30]. Briefly, multi-chromosome intermingling of each 100-kb particle was measured by Shannon's diversity index of chromosomes ($-\Sigma pi \ln pi$, where pi represents the fraction of nearby particles [particles within three particle radii] located in chromosome *i*).

Von Neumann Entropy (VNE) of Intra-chromosomal Contacts

VNE were used to quantify the order of chromatin structure based on normalized 100-kb intra-chromosomal contract matrices as previously described [31]. In brief, for the intra-chromosomal Hi-C matrix (A) of each autosome, we first calculated the Pearson's correlation matrix C = corr (log2(A)). Then, we performed eigen-decomposition on correlation matrix C and generated normalized eigenvalues:

$$\bar{\lambda} i = \frac{\lambda i}{\sum_{j=1}^{n} \lambda j}$$

(λ are the eigenvalues of correlation matrix C, λ1 ≤ λi ≤ λn). Finally, VNE was calculated using the following formula:

$$\text{VNE} = -\sum_{i=1}^{n} \bar{\lambda} i \ln (\bar{\lambda} i)$$

Analysis of A/B Compartments

Identification of A/B compartments was performed in two steps at 20-kb resolution. First, principal components

analysis (PCA) was performed on the Hi-C observed/expected matrix as previously described [32] to generate PC1 values (the first eigenvector) at 100-kb resolution. Compartments A and B were determined by the PC1 values. If a positive Pearson's correlation between PC1 values and gene density were observed with bins at 100-kb resolution, we defined bins with positive PC1 values as compartment A, and the reverse for compartment B. If the contrary is observed, we defined bins with negative PC1 values as compartment A, and vice versa for compartment B. Second, the A-B index, which represented the likelihood of a bin (at 20-kb resolution) interacting with A or B compartment (defined at 100-kb resolution), was generated as previously described [33]. Bins at 20-kb resolution with positive or negative A-B index were considered as A or B compartments, respectively.

Compartment strength was calculated using $(AA \times BB)/AB^2$ as previously described [34] at 20-kb resolution, where AA is the average of observed/expected contacts between pairs of bins with compartment A signal, and BB is the average of observed/expected contacts between pairs of bins with compartment B signal. Similarly, AB is average of pairs of bins with compartment A and B signal. We considered regions exhibiting compartment status changes (*i.e.*, from A to B or from B to A) between pgEpiSCs and pEFs as A/B switched regions. Genes with transcription start sites (TSSs) located within A/B switched regions were considered as A/B switched genes.

Analysis of Topologically Associated Domains (TADs)

Topologically associated domains (TADs) were identified for each Hi-C map based on normalized 20-kb contract maps using directionality index (DI) and HMM (hidden Markov model) implemented in the DomainCaller tool [35]. Briefly, DI was calculated using the contacts between each bin and 2-Mb regions upstream and downstream from that specific bin at 20-kb resolution. HMM was then used to predict DI states for final TAD generation. The inter-TAD regions less than 400-kb in length were defined as TAD boundaries, and those longer than 400-kb were defined as unorganized chromatin regions. Next, we selected pgEpiSC-1-B and pEF-1-G with similar sequencing depths to represent their respective cell type and identified cell-type specific TAD boundaries by applying a method as previously described [35]. For each TAD boundary, we calculated the Spearman's correlation between cell types using DIs assigned to bins around the center of the boundary (+/- 10 bins). Then, we randomly selected 20 bins in each of two cell types and calculated the Spearman's correlation between cell types using DIs to obtain a random correlation, which were repeated 10 000 times to generate a random distribution of correlation coefficients. A cell-type specific boundary was determined as the boundary identified in only one cell type that lacked a significant correlation of DIs between the two cell types (*i.e.*, $r < 0.95$, $P > 0.05$).

The strength of intra-TAD contacts for each TAD were calculated according to a previously described method [36] with minor modifications. In brief, we chose a neighbor isosceles trapezoid range around each TAD. At a certain genomic distance, d, we have two types of contact frequencies, including contacts within TAD (I_{d_TAD}) at distance d and contacts outside TAD but at distance d in the neighboring isosceles trapezoid (I_{d_outTAD}). The strength of intra-TAD contacts = $\frac{\sum d \ (\mathrm{mean}\ (Id\_TAD)/\mathrm{mean}\ (Id\_outTAD))}{\mathrm{n}}$. The insulation score of each 20-kb bin was calculated as previously described [37].

Average TAD and Intra- or Inter-TAD Contact Enrichment

To obtain an enrichment map for intra- or inter-TADs, we extended each TAD region of length x upstream and downstream by x, separately. We further binned the extended 2D interval into a 100 × 100 matrix. As each TAD varies in length, the binned matrix had distinct sizes. We obtained expected contacts at different distances calculated from the average contacts of the 2D bins at the respective distances. We then calculated log ratio of the observed over the expected contacts in each 2D bin of the 100 × 100 matrix per TAD. The average

values in each 2D bin across all TADs for a given cell type are shown in Fig. 5g.

Identification of Promoter-Enhancer Interactions (PEIs)

To identify PEIs at a high resolution of 5-kb, we combined the Hi-C data from 16 replicates for pgEpiSCs and pEFs, separately (Supplementary information, Data S1-I d) and generated their respective Hi-C maps. Promoter regions were defined as regions of 2 200 bp upstream to 500 bp downstream from the TSS. Putative PEIs were identified by applying PSYCHIC [38] based on 5-kb contact matrices. Briefly, the genome was divided into TADs and similar neighboring domains were further merged into a hierarchical structure. Then, a domain-specific background model was built according to the fitted bilinear power-law model for each TAD or merged region. Putative PEIs were identified using interaction intensity normalized by the background model with FDR values < 0.001 and interaction distance ≥ 20 kb.

Beyond characterizing the spatial proximity, we further measured activities of putative enhancers involved in PEIs by analyzing the distribution of acetylation of H3K27 (H3K27ac, a canonical histone mark of enhancers). Excluding H3K27ac peaks with more than 50% of regions located within ± 2 kb region from RefSeq annotated TSSs of genes, we obtained H3K27ac peaks that were defined as confident enhancers. Then, super enhancers (SEs) were identified using the Rank Ordering of Super-Enhancers (ROSE) algorithm (version 0.1) [39,40] based on the H3K27ac signal with default parameters. The remaining active enhancers not marked as SEs were defined as regular enhancers (REs). Then, we defined REs involved in PEIs if the 5-kb enhancer bin overlapped with REs by at least 1-bp in length. SEs were considered to be involved in PEIs if more than half of the 5-kb enhancer bin overlapped with SEs.

Calculation of Regulatory Potential Score (RPS)

To explore the regulatory effects of multi-enhancers on a gene, in order to accurately elucidate dynamic PEIs architecture contributing to the functional transcriptomic divergence between pgEpiSCs and pEFs, we calculated regulatory potential score (RPS) for each promoter (see Materials and methods; Supplementary information, Fig. S8c). RPSs were based on the biochemical assumption that an enhancer's quantitative effect on the expression level of a gene should depend on its spatial proximity, and that the relative contribution of each enhancer is additive. The RPS for each promoter is calculated as $\sum n\ (\log_{10} I_n)$, where I_n is normalized interaction intensity of PEI n for this promoter.

Identification of Intra-Chromosomal Interactions Between Promoters

After separately combining the Hi-C data for replicates of pgEpiSCs and pEFs, we used HiCCUPS [41] to identify interactions between promoters within the same chromosome based on the Hi-C maps at 20-kb resolution with slightly modified parameters. For donut filter, we set the parameters P = 1, w = 3 at 20-kb resolution. We set the average FDR<0.1 for all four local neighborhood backgrounds, and required that the center pixel be enriched by at least 50% over the horizontal, vertical, lower-left and donut expected values. To obtain high confidence interactions, we also required that each pixel be enriched by at least 1.5-fold above either the donut or the lower-left expected value.

Analysis of Publicly Available Hi-C Data and RNA-seq Data for Human and Mouse

To test whether genomic structural features of pgEpiSCs are similar to those of human and mouse embryonic stem cells, we downloaded *in situ* Hi-C data of human embryonic stem cells (hESCs, three biological replicates, GSE52457, GSE105028) [42,43], human dermal fibroblasts (hDFs, ten biological replicates, GSE123552) [44], mouse embryonic stem cells (mESCs, two biological replicates, GSE96107) [45] and mouse embryonic fibroblasts (derived from back skin) (mEFs, two biological replicates, GSE113339) [46].

Hi-C reads of human and mouse were mapped to their respective reference genomes (human: hg38; mouse: mm10) using the Juicer pipeline. A/B compartments were identified using the same strategy as that used for pig data at 20-kb resolution. TADs were obtained at 20-kb resolution based on aggregated Hi-C maps in each cell type using the DI-based method.

RNA-seq data of human (hDFs: GSE78670 [47]; naïve and conventional PSCs: E-MTAB-5674 [48]; GSE69692 [49]; formative PSCs: GSE131556 [50]) and mouse (mEFs: GSE113431 [46]; mESCs: GSE121171 [51]; naïve, formative and primed PSCs: GSE131556 [50]) were downloaded from previous studies and analyzed using the same pipelines as those used for pig.

Functional Enrichment Analysis

Functional enrichment analysis of selected genes was performed using Metascape (http://metascape.org) [52]. The pig genes were mapped to their human orthologs and human (*Homo sapiens*) was the target species for analysis. Enrichment analyses were performed using all genes in the genome as the background set with Gene Ontology (GO)-biological processes (GO-BP) and Kyoto Encyclopedia of Genes and Genomes (KEGG) pathway as ontology sources. Terms with a minimum count ≥ 3, adjusted $P < 0.01$, and enrichment factor ≥ 1.5 were considered to be significant and similar terms were grouped into clusters. The most statistically significant terms in each cluster were depicted using $-\log_{10}$ (*P*-value) bar plots.

Resequencing Data Processing and Variation Calling

The whole genome sequencing data of 19 pgEpiSC lines (~24.42 × sequencing depth for each line) derived from four different donors (of which, three donors are full-sibs) after multi-passages (Supplementary information, Fig. S4b) were separately mapped to the reference pig genome (Suscrof.11.1) using BWA software (version.0.7.8) with default options [24]. We used the 'MarkDuplicates' module in package Picard (version.1.48) to remove duplicated reads. The module 'HaplotypeCaller' in Genome Analysis Toolkit (GATK; version.3.7) [53] was used to identify SNVs and short InDels (≤30 bp), which were further filtered by the following criteria: QUAL < 30.0, QD < 2.0, MQ < 40.0, FS > 60.0. Sex chromosomes were excluded for variation calling. We also removed SNVs and InDels with depth less than 10 × coverage for each sample.

The package ANNOVAR [54] was used to annotate the location of each SNV and InDel with respect to genes. In detail, 'Upstream' refers to a variant that overlaps with the 1 kb region upstream of the gene start site. 'Stop gain' means that an nsSNV leads to the creation of a stop codon at the variant site. 'Stop loss' means that an nsSNV leads to the elimination of a stop codon at the variant site. 'Splicing' means that a variant is within 2 bp of a splice junction. 'Downstream' means that a mutation overlaps with the 1 kb region downstream of the gene end site. 'Upstream/Downstream' means that a variant is located in both downstream and upstream regions (possibly for two different genes).

Reference:

1 Gao, S. *et al.* Tracing the temporal-spatial transcriptome landscapes of the human fetal digestive tract using single-cell RNA-sequencing. *Nat. Cell Biol.* **20**, 721-734 (2018).

2 Islam, S. *et al.* Highly multiplexed and strand-specific single-cell RNA 5' end sequencing. *Nat. Protoc.* **7**, 813-828 (2012).

3 Dobin, A. *et al.* STAR: ultrafast universal RNA-seq aligner. *Bioinformatics* **29**, 15-21 (2013).

4 Liao, Y., Smyth, G. K. & Shi, W. The Subread aligner: fast, accurate and scalable read mapping by seed-and-vote. *Nucleic Acids Res.* **41**, e108 (2013).

5 Smith, T., Heger, A. & Sudbery, I. UMI-tools: modeling sequencing errors in Unique Molecular Identifiers to improve quantification accuracy. *Genome Res.* **27**, 491-499 (2017).

6 Puram, S. V. *et al.* Single-Cell Transcriptomic Analysis of Primary and Metastatic Tumor Ecosystems in Head and Neck Cancer. *Cell* **171**, 1611-1624 e1624 (2017).

7 Stuart, T. *et al.* Comprehensive Integration of Single-Cell Data. *Cell* **177**, 1888-1902 e1821 (2019).

8 Edgar, R. *et al.* LifeMap Discovery™: The Embryonic Development, Stem Cells, and Regenerative Medicine Research Portal. *PLoS One* **8**, e66629 (2013).

9 Aibar, S. *et al.* SCENIC: single-cell regulatory network inference and clustering. *Nat. Methods* **14**, 1083-1086 (2017).

10 Langfelder, P. & Horvath, S. WGCNA: an R package for weighted correlation network analysis. *BMC Bioinformatics* **9**, 559 (2008).

11 Shannon, P. *et al.* Cytoscape: a software environment for integrated models of biomolecular interaction networks. *Genome Res.* **13**, 2498-2504 (2003).

12 Cao, J. *et al.* The single-cell transcriptional landscape of mammalian organogenesis. *Nature* **566**, 496-502 (2019).

13 La Manno, G. *et al.* RNA velocity of single cells. *Nature* **560**, 494-498 (2018).

14 Bray, N. L., Pimentel, H., Melsted, P. & Pachter, L. Near-optimal probabilistic RNA-seq quantification. *Nat. Biotechnol.* **34**, 525-527 (2016).

15 Love, M. I., Huber, W. & Anders, S. Moderated estimation of fold change and dispersion for RNA-seq data with DESeq2. *Genome Biol.* **15**, 550 (2014).

16 Langmead, B. & Salzberg, S. L. Fast gapped-read alignment with Bowtie 2. *Nat. Methods* **9**, 357-359 (2012).

17 Li, H. *et al.* The Sequence Alignment/Map format and SAMtools. *Bioinformatics* **25**, 2078-2079 (2009).

18 Quinlan, A. R. & Hall, I. M. BEDTools: a flexible suite of utilities for comparing genomic features. *Bioinformatics* **26**, 841-842 (2010).

19 Zhang, Y. *et al.* Model-based analysis of ChIP-Seq (MACS). *Genome Biol.* **9**, R137 (2008).

20 Anders, S., Pyl, P. T. & Huber, W. HTSeq--a Python framework to work with high-throughput sequencing

data. *Bioinformatics* **31**, 166-169 (2015).

21 McCarthy, D. J., Chen, Y. & Smyth, G. K. Differential expression analysis of multifactor RNA-Seq experiments with respect to biological variation. *Nucleic Acids Res.* **40**, 4288-4297 (2012).

22 Bailey, T. L. *et al.* MEME SUITE: tools for motif discovery and searching. *Nucleic Acids Res.* **37**, W202-208 (2009).

23 McLeay, R. C. & Bailey, T. L. Motif Enrichment Analysis: a unified framework and an evaluation on ChIP data. *BMC Bioinformatics* **11**, 165 (2010).

24 Li, H. & Durbin, R. Fast and accurate short read alignment with Burrows-Wheeler transform. *Bioinformatics* **25**, 1754-1760 (2009).

25 Zang, C. *et al.* A clustering approach for identification of enriched domains from histone modification ChIP-Seq data. *Bioinformatics* **25**, 1952-1958 (2009).

26 Durand, N. C. *et al.* Juicer Provides a One-Click System for Analyzing Loop-Resolution Hi-C Experiments. *Cell Syst.* **3**, 95-98 (2016).

27 Fletez-Brant, K., Qiu, Y., Gorkin, D. U., Hu, M. & Hansen, K. D. Removing unwanted variation between samples in Hi-C experiments. *bioRxiv*, 214361 (2021).

28 Yang, T. *et al.* HiCRep: assessing the reproducibility of Hi-C data using a stratum-adjusted correlation coefficient. *Genome Res.* **27**, 1939-1949 (2017).

29 Rieber, L. & Mahony, S. miniMDS: 3D structural inference from high-resolution Hi-C data. *Bioinformatics* **33**, i261-i266 (2017).

30 Tan, L., Xing, D., Chang, C. H., Li, H. & Xie, X. S. Three-dimensional genome structures of single diploid human cells. *Science* **361**, 924-928 (2018).

31 Lindsly, S. *et al.* 4DNvestigator: Time Series Hi-C and RNA-seq Data Analysis Toolbox. *bioRxiv*, 2020.2001.2008.898387 (2021).

32 Lieberman-Aiden, E. *et al.* Comprehensive mapping of long-range interactions reveals folding principles of the human genome. *Science* **326**, 289-293 (2009).

33 Rowley, M. J. *et al.* Evolutionarily Conserved Principles Predict 3D Chromatin Organization. *Mol. Cell* **67**, 837-852 e837 (2017).

34 Flyamer, I. M. *et al.* Single-nucleus Hi-C reveals unique chromatin reorganization at oocyte-to-zygote transition. *Nature* **544**, 110-114 (2017).

35 Dixon, J. R. *et al.* Topological domains in mammalian genomes identified by analysis of chromatin interactions. *Nature* **485**, 376-380 (2012).

36 Ke, Y. *et al.* 3D Chromatin Structures of Mature Gametes and Structural Reprogramming during Mammalian Embryogenesis. *Cell* **170**, 367-381 e320 (2017).

37 Crane, E. *et al.* Condensin-driven remodelling of X chromosome topology during dosage compensation. *Nature* **523**, 240-244 (2015).

38 Ron, G., Globerson, Y., Moran, D. & Kaplan, T. Promoter-enhancer interactions identified from Hi-C data using probabilistic models and hierarchical topological domains. *Nat. Commun.* **8**, 2237 (2017).

39 Whyte, W. A. *et al.* Master transcription factors and mediator establish super-enhancers at key cell identity genes. *Cell* **153**, 307-319 (2013).

40 Loven, J. *et al.* Selective inhibition of tumor oncogenes by disruption of super-enhancers. *Cell* **153**, 320-334 (2013).

41 Rao, S. S. *et al.* A 3D map of the human genome at kilobase resolution reveals principles of chromatin looping. *Cell* **159**, 1665-1680 (2014).

42 Lyu, X., Rowley, M. J. & Corces, V. G. Architectural Proteins and Pluripotency Factors Cooperate to Orchestrate the Transcriptional Response of hESCs to Temperature Stress. *Mol. Cell* **71**, 940-955 e947 (2018).

43 Dixon, J. R. *et al.* Chromatin architecture reorganization during stem cell differentiation. *Nature* **518**, 331-336 (2015).

44 Nir, G. *et al.* Walking along chromosomes with super-resolution imaging, contact maps, and integrative modeling. *PLoS Genet.* **14**, e1007872 (2018).

45 Bonev, B. *et al.* Multiscale 3D Genome Rewiring during Mouse Neural Development. *Cell* **171**, 557-572 e524 (2017).

46 Di Giammartino, D. C. *et al.* KLF4 is involved in the organization and regulation of pluripotency-associated three-dimensional enhancer networks. *Nat. Cell Biol.* **21**, 1179-1190 (2019).

47 Consortium, E. P. An integrated encyclopedia of DNA elements in the human genome. *Nature* **489**, 57-74 (2012).

48 Guo, G. *et al.* Epigenetic resetting of human pluripotency. *Development* **144**, 2748-2763 (2017).

49 Ji, X. *et al.* 3D Chromosome Regulatory Landscape of Human Pluripotent Cells. *Cell Stem Cell* **18**, 262-275 (2016).

50 Kinoshita, M. *et al.* Capture of Mouse and Human Stem Cells with Features of Formative Pluripotency. *Cell Stem Cell* **28**, 453-471 e458 (2021).

51 Shukla, R. *et al.* Activation of transcription factor circuity in 2i-induced ground state pluripotency is independent of repressive global epigenetic landscapes. *Nucleic Acids Res.* **48**, 7748-7766 (2020).

52 Zhou, Y. *et al.* Metascape provides a biologist-oriented resource for the analysis of systems-level datasets. *Nat. Commun.* **10**, 1523 (2019).

53 McKenna, A. *et al.* The Genome Analysis Toolkit: a MapReduce framework for analyzing next-generation DNA sequencing data. *Genome Res.* **20**, 1297-1303 (2010).

54 Wang, K., Li, M. & Hakonarson, H. ANNOVAR: functional annotation of genetic variants from high-throughput sequencing data. *Nucleic Acids Res.* **38**, e164 (2010).
