# Supplementary material for: Generation and characterization of stable pig pregastrulation epiblast stem cell lines
Source: Cell Res. 2021 Nov 30;32(4):383–400. doi: 10.1038/s41422-021-00592-9 (PMC8976023; doi:10.1038/s41422-021-00592-9)
Supplement: Supplementary file 19 — Supplementary information, Table S6 [file 41422_2021_592_MOESM19_ESM.pdf]

## Supplementary information, Table S6, Key Resources Table

| REAGENT or RESOURCE | SOURCE | IDENTIFIER |
|---|---|---|
| Antibodies | | |
| Rabbit polyclonal anti-neuron specific beta III Tubulin | Abcam | Cat# ab18207, RRID: AB_444319 |
| Rabbit polyclonal anti-alpha smooth muscle Actin | Abcam | Cat# ab5694, RRID: AB_2223021 |
| Rabbit monoclonal anti-GATA-6 (D61E4) | Cell Signaling Technology | Cat# 5851, RRID: AB_10705521 |
| Mouse monoclonal anti-SSEA1 (MC480) | Abcam | Cat# ab16285, RRID: AB_870663 |
| Mouse monoclonal anti-SSEA4 (MC813) | Abcam | Cat# ab16287, RRID: AB_778073 |
| Mouse monoclonal anti-human-TRA-1-60 | Cell Signaling Technology | Cat# 4746, RRID: AB_2119059 |
| Mouse monoclonal anti-human-TRA-1-81 | Cell Signaling Technology | Cat# 4745, RRID: AB_2119060 |
| Rabbit polyclonal anti-SOX1 | Abcam | Cat# ab87775, RRID: AB_2616563 |
| Rabbit polyclonal anti-Brachyury/Bry | Abcam | Cat# ab20680, RRID: AB_727024 |
| Rabbit polyclonal anti-H3K27ac | Abcam | Cat# ab4729; RRID: AB_2118291 |
| Rabbit polyclonal anti-TBR2 / Eomes | Abcam | Cat# ab23345, RRID: AB_778267 |
| Mouse monoclonal anti-E Cadherin | Abcam | Cat# ab76055, RRID: AB_1310159 |
| Mouse monoclonal anti-Oct-3/4 (C-10) | Santa Cruz Biotechnology | Cat# sc-5279, RRID: AB_628051 |
| Mouse monoclonal anti-Sox-2 (E-4) | Santa Cruz Biotechnology | Cat# sc-365823, RRID: AB_10842165 |
| Rabbit polyclonal anti-human Nanog | PeproTech | Cat# 500-P236, RRID: AB_1268274 |
| Rabbit monoclonal anti-Phospho-Stat3 (Tyr705) (D3A7) | Cell Signaling Technology | Cat# 52075, RRID: AB_2799407 |
| Rabbit monoclonal anti-Stat3 (D3Z2G) | Cell Signaling Technology | Cat# 12640S, RRID: AB_2629499 |
| Rabbit monoclonal anti-GAPDH (D16H11) | Cell Signaling Technology | Cat# 5174, RRID: AB_10622025 |
| Goat anti-Mouse IgG (H+L) Cross-Adsorbed Secondary Antibody, Alexa Fluor 488 | Thermo Fisher Scientific | Cat# A-11001, RRID: AB_2534069 |
| Goat anti-Mouse IgG (H+L) Cross-Adsorbed Secondary Antibody, Alexa Fluor 594 | Thermo Fisher Scientific | Cat# A-11005, RRID: AB_141372 |
| Donkey anti-Rabbit IgG (H+L) Highly Cross-Adsorbed Secondary Antibody, Alexa Fluor 594 | Thermo Fisher Scientific | Cat# A-21207, RRID: AB_141637 |

| | | |
|---|---|---|
| Donkey anti-Rabbit IgG (H+L) Highly Cross-Adsorbed Secondary Antibody, Alexa Fluor 488 | Thermo Fisher Scientific | Cat# A-21206, RRID: AB_2535792 |
| Chemicals, Peptides, and Recombinant Proteins | | |
| CHIR99021 | Selleckchem | Cat# S1263 |
| IWR-1-endo | Selleckchem | Cat# S7086 |
| Y-27632 | Selleckchem | Cat# S1049 |
| WH-4-023 | Selleckchem | Cat# S7565 |
| SB431542 | Selleckchem | Cat# S1067 |
| Ruxolitinib (INCB018424) | Selleckchem | Cat# S1378 |
| PD0325901 | Selleckchem | Cat# S1036 |
| Recombinant Murine BMP-4 | PeproTech | Cat# 315-27 |
| Human/Murine/Rat Activin A (E.Coli) | PeproTech | Cat# 120-14E |
| Recombinant Human LIF | PeproTech | Cat# 300-05 |
| Recombinant Human FGF-basic (154 a.a.) | PeproTech | Cat# 100-18B |
| All-Trans Retinoic Acid | PeproTech | Cat# 3027949 |
| Recombinant Human Noggin | PeproTech | Cat# 120-10C |
| Bovine Serum Albumin | Sigma-Aldrich | Cat# A1470 |
| Ascorbic Acid | Sigma-Aldrich | Cat# A4544 |
| KnockOut Serum Replacement | Thermo Fisher Scientific | Cat# A3181502 |
| Neurobasal™ Medium | Thermo Fisher Scientific | Cat# 21103-049 |
| DMEM/F-12, GlutaMAX™ supplement | Thermo Fisher Scientific | Cat# 10565-018 |
| N-2 Supplement (100 ×) | Thermo Fisher Scientific | Cat# 17502-048 |
| B-27™ Supplement (50 ×), minus vitamin A | Thermo Fisher Scientific | Cat# 12587-010 |
| GlutaMAX™ Supplement | Thermo Fisher Scientific | Cat# 35050-061 |
| MEM Non-Essential Amino Acids Solution (100 ×) | Thermo Fisher Scientific | Cat# 11140-050 |
| 2-Mercaptoethanol | Thermo Fisher Scientific | Cat# 21985-023 |
| Penicillin-Streptomycin (10,000 U/mL) | Thermo Fisher Scientific | Cat# 15140-122 |
| Gelatin (0.1% in water) | Stem Cell Technologies | Cat# 07903 |
| Trypsin-EDTA (0.05%), phenol red | Gibco | Cat# 25300120 |
| DMEM, high glucose, no glutamine | Gibco | Cat# 11960-044 |
| Fetal bovine serum (FBS) | Gibco | Cat# 16000-044 |
| Accutase cell dissociation reagent | Gibco | Cat# A11105-01 |
| TrypLE ™ Express | Gibco | Cat# 12605010 |
| Dulbecco's phosphate-buffered saline (DPBS) | Gibco | Cat# C14190500CP |
| Critical Commercial Assays | | |
| KAPA Hyper Prep Kits | KAPA Biosystems | Cat# KK8054 |
| RNeasy Mini Kit | QIAGEN | Cat# 74106 |
| Globin-Zero Gold rRNA Removal Kit | Illumina | Cat# GZG1224 |
| NEBNext® Ultra™ Directional RNA Library Prep Kit for Illumina® | New England Biolabs | Cat# E7760 |
| NEXTflex™ ChIP-Seq Kit | Bioo Scientific | Cat# NOVA-5143-02 |
| RNAprep pure Cell / Bacteria Kit | TIANGEN | Cat# DP430 |
| 5 × All-In-One RT Master Mix | ABM | Cat# G490 |
| TIANamp Genomic DNA Kit | TIANGEN | Cat# DP304 |
| Rapid Giemsa staining kit | BBI Life Sciences | Cat# E202FA0001 |
| Gel & PCR Clean Up Kit | OMEGA | Cat# D2000-02 |
| Lipofectamine ™ 3000 Transfection Kit | Invitrogen | Cat# 2173193 |

| | | |
|---|---|---|
| Lipofectamine ™ Stem Transfection Reagent | Invitrogen | Cat# STEM00008 |
| Endo-free Plasmid Mini Kit | OMEGA | Cat# D6950-02B |
| Bradford Protein Assay Kit | BIO-RED | Cat# 5000201 |
| 2 × RealStar Green Power Mixture | GenStar | Cat# A311-05 |
| Cells-to-cDNA™ II Kit | Invitrogen | Cat# AM8723 |
| Deposited Data | | |
| Pig preimplantation embryos and pgEpiSCs scRNA-seq data | This paper | CRA003960 |
| Whole genome resequencing of pgEpiSCs | This paper | CRA003960 |
| Raw and analyzed Hi-C data for 16 libraries in pgEpiSCs and 16 libraries in pEFs | This paper | CRA003960 |
| Raw RNA-seq data for 4 libraries in pgEpiSCs and 2 libraries in pEFs | This paper | CRA003960 |
| H3K27ac ChIP-seq for 2 libraries in pgEpiSCs and 2 libraries in pEFs | This paper | CRA003960 |
| ATAC-seq for 3 libraries in pgEpiSCs and 2 libraries in pEFs | This paper | CRA003960 |
| Human ESCs Hi-C data | [1,2] | GSE52457, GSE105028 |
| Human dermal fibroblasts Hi-C data | [3] | GSE123552 |
| Mouse ESCs Hi-C data | [4,5] | GSE96107, GSE124342 |
| Mouse embryonic fibroblasts Hi-C data | [6,7] | GSE113339 |
| Human ESCs RNA-seq data | [8] | GSE69692 |
| Human dermal fibroblasts RNA-seq data | [9] | GSE78670 |
| Mouse ESCs RNA-seq data | [10] | GSE121171 |
| Mouse embryonic fibroblasts RNA-seq data | [6] | GSE113431 |
| Human formative PSCs, mouse naive, formative and primed PSCs RNA-seq data | [11] | GSE131556 |
| Human naive and conventional PSCs RNA–seq data | [12] | E-MTAB-5674 |
| Human naive and conventional PSCs RNA–seq data | [8] | GSE69692 |
| pEPSC_Gao RNA-seq data | [13] | E-MTAB-7253 |
| pESC_Choi RNA-seq data | [14] | GSE120031 |
| piPSC_Secher RNA-seq data | [15] | GSE92889 |
| pESCLC_Yuan and piPSC_Yuan RNA-seq data | [16] | GSE126150 |
| piPSC_Shi RNA-seq data | [17] | GSE143484 |
| piPSC_Xu RNA-seq data | [18] | GSE141935 |
| Experimental Models: Cell Lines | | |
| 1-pgEpiSCs | This paper | N/A |
| 2-pgEpiSCs | This paper | N/A |
| 3-pgEpiSCs | This paper | N/A |
| 4-pgEpiSCs | This paper | N/A |
| PEF | This paper | N/A |
| MEF | This paper | N/A |
| Experimental Models: Organisms | | |
| CD-1® (ICR) IGS Mice | Vital River | 201 |
| BALB/c Nude Mice | Vital River | 401 |

| | | |
|---|---|---|
| BAMA pigs | Beijing Farm Animal Research Center | N/A |
| Nongda pigs | CAU Experimental Miniature Pig Farm | N/A |
| DLY pigs | YAU/NEAU Experimental Pig Farm | N/A |
| Oligonucleotides | | |
| TSO primer:<br>AAGCAGTGGTATCAACGCAGAGTACATrGrG+G | This paper | N/A |
| ISPCR:<br>AAGCAGTGGTATCAACGCAGAGT | This paper | N/A |
| 3'-P2:<br>GTGACTGGAGTTCAGACGTGTGCTCTTCCGATC | This paper | N/A |
| short universal primer:<br>AATGATACGGCGACCACCGAGATCTACACTCTTT<br>CCCTACACGAC | This paper | N/A |
| illunima-QP2:<br>CAAGCAGAAGACGGCATACGA | This paper | N/A |
| *POU5F1*:<br>Forword: CAAACTGAGGTGCCTGCCCTTC<br>Reverse: ATTGAACTTCACCTTCCCTCCAACC | This paper | N/A |
| *NANOG*:<br>Forword: CATCTGCTGAGACCCTCGAC<br>Reverse: GGGCTTGTGGAAGAATCAGG | This paper | N/A |
| *SOX2*:<br>Forword: CATCAACGGTACACTGCCTCTC<br>Reverse: ACTCTCCTCCCATTTCCCTCTTT | This paper | N/A |
| *REX1(ZFP42)*:<br>Forword: GGGATCACGTGTGTGCAGAA<br>Reverse: TTCCTGCGACAGCCTTCAAA | This paper | N/A |
| *CDH1*:<br>Forword: GACTTCTGCCAGAGGAACCC<br>Reverse: CACTGGCCCCATGTGTTAGT | This paper | N/A |
| *IGF2*:<br>Forword: CGTGCTGCTATGCTGCTTAC<br>Reverse: AAGCAGCACTCTTCCACGAT | This paper | N/A |
| *SNAI2*:<br>Forword: CCCACACCCTACCTTGTGTC<br>Reverse: TGACATCCGAGTGTGTCTGC | This paper | N/A |
| *SRC*:<br>Forword: GCCAACATCCTGGTTGGAGA<br>Reverse: ATCCCAAAGGACCACACGTC | This paper | N/A |
| *WNT5A*:<br>Forword: CGCGAAGACAGGCATCAAAG<br>Reverse: CCTATCTGCATGACCCTGCC | This paper | N/A |

| | | |
|---|---|---|
| *GATA6*:<br>Forword: CACTACTTGTGCAACCGCTG<br>Reverse: TTCTGCGGCTTTATGAGGGG | This paper | N/A |
| *HAND1*:<br>Forword: TGCGAGTGCATACCTTCTGT<br>Reverse: AGGCCCTGAGGGGAGTTTAT | This paper | N/A |
| *PAX6*:<br>Forword: TGTCCAACGGATGTGTGAGT<br>Reverse: TCTGTCTCGGATTTCCCAA | This paper | N/A |
| *CDH2*:<br>Forword: GCCTCAAGCCAACCTTACCT<br>Reverse: AGCTCTTGAGGAAAAGGCCC | This paper | N/A |
| *ID2*:<br>Forword: TCGCACCCCACTATTGTCAG<br>Reverse: TTCAGAAGCCTGCAAGGACA | This paper | N/A |
| *ID3*:<br>Forword: CATCTTCCCATCCAGACAGCC<br>Reverse: GTCAAGTGGGCACGACAAAG | This paper | N/A |
| *BMP2*:<br>Forword: GTCTTTCGGGAGCAGACACA<br>Reverse: GTCACCAACCTGGTGTCCAA | This paper | N/A |
| *EOMES*:<br>Forword: ACTCCCATGGACCTCCAGAA<br>Reverse: TCGCTTACAAGCACTGGTGT | This paper | N/A |
| *T*:<br>Forword: GAAGTACGTGAACGGGGAGT<br>Reverse: CACGATGTGGATTCGAGGCT | This paper | N/A |
| *LIN28A*:<br>Forword: TGCCGGCATCTGTAAATGGT<br>Reverse: ACTCTGGTGCACAAAGACGT | This paper | N/A |
| *C-MYC*:<br>Forword: ATCCAAGACCACCACCACTG<br>Reverse: GTTCACAGCAACATTCAGGTAGA | This paper | N/A |
| *ETV4*:<br>Forword: AGAACCGGCCAGCTATGAAC<br>Reverse: ATTGTCCGGGAAAGCCAGAG | This paper | N/A |
| *ETV5*:<br>Forword: TCAGCACATGGGTTCCAGTC<br>Reverse: CCTTCATGGCTGCTGGAGAA | This paper | N/A |
| *BMP4*:<br>Forword: TTCATTTTAGGAGCCATTCTGTAGT<br>Reverse: TCCTAGCAGGACTTGGCATAAT | This paper | N/A |
| *EF-1α*:<br>Forword: AATGCGGTGGGATCGACAAA<br>Reverse: CACGCTCACGTTCAGCCTTT | This paper | N/A |

| | | |
|---|---|---|
| *TYR* gene sgRNA targeting sequence:<br>Forword: ACCGACCTCAGTTCCCCTTCACCG<br>Reverse: AAACCGGTGAAGGGGAACTGAGGT | This paper | N/A |
| *TYR* gene mutant site test primer:<br>Forword: CTGGACTTTCCAGACTTCCG<br>Reverse: GTGCAGTTGGGTCCCTGAAA | This paper | N/A |
| *GFP* test primer<br>Forword: CAACACCCGCATCGAGAAGT<br>Reverse: ACCACGAAGCTGTAGTAGCC | This paper | N/A |
| *NANOG* knock-in 3' ARM test primer<br>Forword: GCAACCTCCCCTTCTACGAG<br>Reverse: GTGAAGCACACGGTAGGGTA | This paper | N/A |
| *NANOG* knock-in 5' ARM test primer<br>Forword: TGTCCATTGCTGAAGCATGTAAT<br>Reverse: GAAGTTAGTAGCTCCGCTTCCTG | This paper | N/A |
| Recombinant DNA | | |
| pGL3-U6-gRNA-EGFP | Xingxu, Huang's Lab | N/A |
| pCMV_AncBE4max | Xingxu, Huang's Lab | N/A |
| PB-CMV-EF1A-GFP-NLS | This paper | N/A |
| Nanog-tdTomato-donor vector | Our Laboratory | N/A |
| Software and Algorithms | | |
| Snap Gene software | Insightful Science | https://www.snapgene.com |
| CRISPR sgRNA Design Tool | [19] | https://www.genscript.com/gRNA-design-tool |
| STAR version 2.7.1a | [20] | https://github.com/alexdobin/STAR |
| Subread version 1.6.4 | [21] | https://sourceforge.net/projects/subread |
| SAMtools version 1.9 | [22] | http://samtools.sourceforge.net |
| UMI-tools | [23] | https://github.com/CGATOxford/UMI-tools |
| R version 3.6.0 | N/A | https://www.R-project.org |
| Rstudio | N/A | https://rstudio.com |
| Seurat (verison 3.0.0) | [24] | https://satijalab.org/seurat |
| STAR v2.6.0c | [20] | https://github.com/alexdobin/STAR/releases/tag/2.6.0c |
| Kallisto v0.44.0 | [25] | http://pachterlab.github.io/kallisto//releases/2018/01/29/v0.44.0 |

| | | |
|---|---|---|
| DEseq2 v1.28.1 | 26 | https://bioconductor.org/packages/release/bioc/html/DESeq2 |
| BWA v0.7.15 | 27 | https://github.com/lh3/bwa/releases/tag/v0.7.15 |
| SAMtools v0.1.19 | 22 | https://github.com/samtools/samtools/tree/0.1.19 |
| SICER v1.1 | 28 | https://github.com/dariober/SICERpy |
| Juicer | 29 | https://github.com/aidenlab/juicer |
| quantile algorithm | 30 | https://bioconductor.org/packages/release/bioc/html/bnbc |
| HiCRep | 31 | https://www.bioconductor.org/packages/release/bioc/html/hicrep |
| miniMDS | 32 | https://github.com/seqcode/miniMDS |
| PSYCHIC | 33 | https://github.com/dhkron/PSYCHIC |
| ROSE algorithm v0.1 | 34,35 | https://github.com/rakarnik/ROSE |
| HiCCUPS | 36 | https://github.com/aidenlab/juicer/wiki/HiCCUPS |
| Trim_galore | DOI:10.5281/zenodo.5127898 | https://github.com/FelixKrueger/TrimGalore |
| Bowtie2 | 37 | https://github.com/BenLangmead/bowtie2 |
| GATK | 38 | https://github.com/broadinstitute/gatk |
| ANNOVAR | 39 | https://github.com/WGLab/doc-ANNOVAR |
| MACS2 | 40 | https://pypi.org/project/MACS2/ |
| HTSeq | 41 | https://github.com/htseq/htseq |

| | | |
|---|---|---|
| edgeR | 42 | https://bioconductor.org/packages/release/bioc/html/edgeR.html |
| MEME | 43 | https://meme-suite.org/meme/ |
| PyMOL | DeLano, W.L. (2002) The PyMol molecular graphics system | https://pymol.org/2/ |
| BEDtools2 | 44 | https://github.com/arq5x/bedtools2 |
| IGV v2.6.0 | 45 | http://www.igv.org |
| Metascape | 46 | https://metascape.org |
| Pheatmap | N/A | https://cran.r-project.org/web/packages/pheatmap/index |
| ggplot2 | N/A | https://ggplot2.tidyverse.org |
| WGCNA v1.68 | 47 | https://horvath.genetics.ucla.edu/html/CoexpressionNetwork/Rpackages/WGCNA |
| Cytoscape | 48 | https://cytoscape.org |
| AUCell v1.5.2 | 49 | https://bioconductor.org/packages/AUCell |
| Cutadapt v3.0 | 50 | https://cutadapt.readthedocs.io |
| GraphPad Prism 8 | GraphPad Prism | https://www.graphpad.com |
| monocle3 v1.0.0 | 51 | https://cole-trapnell-lab.github.io/monocle3 |
| velocyto | 52 | http://velocyto.org |
| DomainCaller | 53 | https://github.com/XiaoTaoWang/domaincaller |

## Reference:


1 Dixon, J. R. *et al.* Chromatin architecture reorganization during stem cell differentiation. *Nature* **518**, 331-336 (2015).

2 Lyu, X., Rowley, M. J. & Corces, V. G. Architectural Proteins and Pluripotency Factors Cooperate to Orchestrate the Transcriptional Response of hESCs to Temperature Stress. *Mol. Cell* **71**, 940-955 e947 (2018).

3 Nir, G. *et al.* Walking along chromosomes with super-resolution imaging, contact maps, and integrative modeling. *PLoS Genet.* **14**, e1007872 (2018).

4 Bonev, B. *et al.* Multiscale 3D Genome Rewiring during Mouse Neural Development. *Cell* **171**, 557-572 e524 (2017).

5 McLaughlin, K. *et al.* DNA Methylation Directs Polycomb-Dependent 3D Genome Re-organization in Naive Pluripotency. *Cell Rep.* **29**, 1974-1985 e1976 (2019).

6 Di Giammartino, D. C. *et al.* KLF4 is involved in the organization and regulation of pluripotency-associated three-dimensional enhancer networks. *Nat. Cell Biol.* **21**, 1179-1190 (2019).

7 Colognori, D., Sunwoo, H., Kriz, A. J., Wang, C. Y. & Lee, J. T. Xist Deletional Analysis Reveals an Interdependency between Xist RNA and Polycomb Complexes for Spreading along the Inactive X. *Mol. Cell* **74**, 101-117 e110 (2019).

8 Ji, X. *et al.* 3D Chromosome Regulatory Landscape of Human Pluripotent Cells. *Cell Stem Cell* **18**, 262-275 (2016).

9 Consortium, E. P. An integrated encyclopedia of DNA elements in the human genome. *Nature* **489**, 57-74 (2012).

10 Shukla, R. *et al.* Activation of transcription factor circuity in 2i-induced ground state pluripotency is independent of repressive global epigenetic landscapes. *Nucleic Acids Res.* **48**, 7748-7766 (2020).

11 Kinoshita, M. *et al.* Capture of Mouse and Human Stem Cells with Features of Formative Pluripotency. *Cell Stem Cell* **28**, 453-471 e458 (2021).

12 Guo, G. *et al.* Epigenetic resetting of human pluripotency. *Development* **144**, 2748-2763 (2017).

13 Gao, X. *et al.* Establishment of porcine and human expanded potential stem cells. *Nat. Cell Biol.* **21**, 687-699 (2019).

14 Choi, K. H. *et al.* Chemically Defined Media Can Maintain Pig Pluripotency Network In Vitro. *Stem Cell Reports* **13**, 221-234 (2019).

15 Secher, J. O. *et al.* Systematic in vitro and in vivo characterization of Leukemia-inhibiting factor- and Fibroblast growth factor-derived porcine induced pluripotent stem cells. *Mol. Reprod. Dev.* **84**, 229-245 (2017).

16 Yuan, Y. *et al.* A six-inhibitor culture medium for improving naive-type pluripotency of porcine pluripotent stem cells. *Cell Death Discov.* **5**, 104 (2019).

17 Shi, B. *et al.* IRF-1 expressed in the inner cell mass of the porcine early blastocyst enhances the pluripotency of induced pluripotent stem cells. *Stem Cell. Res. Ther.* **11**, 505 (2020).

18 Xu, J. *et al.* A cytokine screen using CRISPR-Cas9 knock-in reporter pig iPS cells reveals that Activin A regulates NANOG. *Stem Cell. Res. Ther.* **11**, 67 (2020).

19 Labun, K. *et al.* CHOPCHOP v3: expanding the CRISPR web toolbox beyond genome editing. *Nucleic Acids Res.* **47**, W171-W174 (2019).

20 Dobin, A. *et al.* STAR: ultrafast universal RNA-seq aligner. *Bioinformatics* **29**, 15-21 (2013).

21 Liao, Y., Smyth, G. K. & Shi, W. The Subread aligner: fast, accurate and scalable read mapping by seed-and-vote. *Nucleic Acids Res.* **41**, e108 (2013).

22 Li, H. *et al.* The Sequence Alignment/Map format and SAMtools. *Bioinformatics* **25**, 2078-2079 (2009).

23 Smith, T., Heger, A. & Sudbery, I. UMI-tools: modeling sequencing errors in Unique Molecular Identifiers to improve quantification accuracy. *Genome Res.* **27**, 491-499 (2017).

24 Stuart, T. *et al.* Comprehensive Integration of Single-Cell Data. *Cell* **177**, 1888-1902 e1821 (2019).

25 Bray, N. L., Pimentel, H., Melsted, P. & Pachter, L. Near-optimal probabilistic RNA-seq quantification. *Nat. Biotechnol.* **34**, 525-527 (2016).

26 Love, M. I., Huber, W. & Anders, S. Moderated estimation of fold change and dispersion for RNA-seq data with DESeq2. *Genome Biol.* **15**, 550 (2014).

27 Li, H. & Durbin, R. Fast and accurate short read alignment with Burrows-Wheeler transform. *Bioinformatics* **25**, 1754-1760 (2009).

28 Zang, C. *et al.* A clustering approach for identification of enriched domains from histone modification ChIP-Seq data. *Bioinformatics* **25**, 1952-1958 (2009).

29 Durand, N. C. *et al.* Juicer Provides a One-Click System for Analyzing Loop-Resolution Hi-C Experiments. *Cell Syst.* **3**, 95-98 (2016).

30 Fletez-Brant, K., Qiu, Y., Gorkin, D. U., Hu, M. & Hansen, K. D. Removing unwanted variation between samples in Hi-C experiments. *bioRxiv*, 214361 (2021).

31 Yang, T. *et al.* HiCRep: assessing the reproducibility of Hi-C data using a stratum-adjusted correlation coefficient. *Genome Res.* **27**, 1939-1949 (2017).

32 Rieber, L. & Mahony, S. miniMDS: 3D structural inference from high-resolution Hi-C data. *Bioinformatics* **33**, i261-i266 (2017).

33 Ron, G., Globerson, Y., Moran, D. & Kaplan, T. Promoter-enhancer interactions identified from Hi-C data using probabilistic models and hierarchical topological domains. *Nat. Commun.* **8**, 2237 (2017).

34 Loven, J. *et al.* Selective inhibition of tumor oncogenes by disruption of super-enhancers. *Cell* **153**, 320-334 (2013).

35 Whyte, W. A. *et al.* Master transcription factors and mediator establish super-enhancers at key cell identity genes. *Cell* **153**, 307-319 (2013).

36 Rao, S. S. *et al.* A 3D map of the human genome at kilobase resolution reveals principles of chromatin looping. *Cell* **159**, 1665-1680 (2014).

37 Langmead, B. & Salzberg, S. L. Fast gapped-read alignment with Bowtie 2. *Nat. Methods* **9**, 357-359 (2012).

38 Van der Auwera, G. A. *et al.* From FastQ data to high confidence variant calls: the Genome Analysis Toolkit best practices pipeline. *Curr Protoc Bioinformatics* **43**, 11.10.11-11.10.33 (2013).

39 Wang, K., Li, M. & Hakonarson, H. ANNOVAR: functional annotation of genetic variants from high-throughput sequencing data. *Nucleic Acids Res.* **38**, e164 (2010).

40 Feng, J., Liu, T., Qin, B., Zhang, Y. & Liu, X. S. Identifying ChIP-seq enrichment using MACS. *Nat. Protoc.* **7**, 1728-1740 (2012).

41 Anders, S., Pyl, P. T. & Huber, W. HTSeq--a Python framework to work with high-throughput sequencing data. *Bioinformatics* **31**, 166-169 (2015).

42 Robinson, M. D., McCarthy, D. J. & Smyth, G. K. edgeR: a Bioconductor package for differential expression analysis of digital gene expression data. *Bioinformatics* **26**, 139-140 (2010).

43 Bailey, T. L., Johnson, J., Grant, C. E. & Noble, W. S. The MEME Suite. *Nucleic Acids Res.* **43**, W39-49 (2015).

44 Quinlan, A. R. BEDTools: The Swiss-Army Tool for Genome Feature Analysis. *Curr Protoc Bioinformatics* **47**, 11.12.11-34 (2014).

45 Thorvaldsdóttir, H., Robinson, J. T. & Mesirov, J. P. Integrative Genomics Viewer (IGV): high-performance genomics data visualization and exploration. *Brief Bioinform* **14**, 178-192 (2013).

46 Zhou, Y. *et al.* Metascape provides a biologist-oriented resource for the analysis of systems-level datasets. *Nat. Commun.* **10**, 1523 (2019).

47 Langfelder, P. & Horvath, S. WGCNA: an R package for weighted correlation network analysis. *BMC Bioinformatics* **9**, 559 (2008).

48 Shannon, P. *et al.* Cytoscape: a software environment for integrated models of biomolecular interaction networks. *Genome Res.* **13**, 2498-2504 (2003).

49 Aibar, S. *et al.* SCENIC: single-cell regulatory network inference and clustering. *Nat. Methods* **14**, 1083-1086 (2017).

50 Martin, M. Cutadapt removes adapter sequences from high-throughput sequencing reads. *EMBnet J.* **17**, 10 (2011).

51 Trapnell, C. *et al.* The dynamics and regulators of cell fate decisions are revealed by pseudotemporal ordering of single cells. *Nat. Biotechnol.* **32**, 381-386 (2014).

52 La Manno, G. *et al.* RNA velocity of single cells. *Nature* **560**, 494-498 (2018).

53 Dixon, J. R. *et al.* Topological domains in mammalian genomes identified by analysis of chromatin interactions. *Nature* **485**, 376-380 (2012).
